# Analyzing the Specificity of KAWLR Genetic Resources in Afghan Landrace Wheat for Ca-Rich High pH Soil Tolerance Using Proteomics

**DOI:** 10.3390/ijms27010239

**Published:** 2025-12-25

**Authors:** Emdadul Haque, Farid Niazi, Xiaojian Yin, Yuso Kobara, Setsuko Komatsu, Tomohiro Ban

**Affiliations:** 1 Kihara Institute for Biological Research, Yokohama City University, Maioka-cho 641-12, Totsuka-ku, Yokohama 244-0813, Japan; faridniazi30@gmail.com (F.N.); tban@yokohama-cu.ac.jp (T.B.); 2 Kyushu Okinawa Agricultural Research Center, NARO, 6651-2 Yokoichi, Miyakonojo 885-0091, Japan; 3 National Institute of Crop Science, National Agriculture and Food Research Organization, Tsukuba 305-8518, Japan; ajian.517@163.com; 4 Key Laboratory of Soybean Molecular Design Breeding, Northeast Institute of Geography and Agroecology, Chinese Academy of Sciences, Changchun 130102, China; 5 Institute for Agro-Environmental Science, National Agriculture and Food Research Organization, Tsukuba 305-8604, Japan; kobara.yuso873@naro.go.jp; 6 Faculty of Environmental and Information Sciences, Fukui University of Technology, Fukui 910-8505, Japan

**Keywords:** Kihara Afghan wheat landrace, root allocation, plant nutrients, Ca-rich high pH soil tolerance, wheat breeding

## Abstract

Breeding wheat varieties that are resilient to arid climates, which impart a complex combination of stresses, including excessive Ca, high pH, nutrient deficiency, and aridity, is important. Afghan landrace wheat is assumed to have evolved with a specific prototypical pattern of traits to adapt to its challenging, composite stress environment. Here, a useful semi-hydroponic double cup screen aiding proteomic analysis was exploited to reconstruct the combined excessive Ca^2+^ (100 ppm) and extreme pH (11.0) of the soils and to dissect specific morpho-physiological characteristics and adaptation strategies in Kihara Afghan wheat landrace (KAWLR). When compared to other cultivars and growth habits, several winter-type KAWLR showed lower unused N-K-P and greater rhizosphere pH stability in the bottom cup and higher tolerance in terms of greater root allocation shift, and most of their above ground traits (shoot biomass, chlorophyll content, and stomatal conductance) were strongly correlated with root length and biomass under stress conditions. Quantitative proteomics on the roots of a tolerant winter-type KAWLR, Herat-740 (KU-7449), showed a strong decreasing trend in changed proteins (12 increased/816 decreased). The proteins (such as mitochondrial phosphate carrier protein, cytoskeleton-related α-, and β-tubulin) that increased in abundance were associated with energy transport and cell growth. A metabolism overview revealed that most proteins that were mapped to glycolysis, fermentation, and the TCA cycle decreased in abundance. However, proteins related to cell wall and lipid metabolism pathways remained unchanged. Our results suggest that winter-type KAWLR adopts a homeostatic stress adaptation strategy that globally downshifts metabolic activity, while selectively maintaining root growth machinery. Root allocation shift, rhizosphere pH stabilization (nutrient solubilization), and a selective proteome response maintaining the root growth machinery in winter-type KAWLR could be breeding selection markers for early-stage screening in calcareous-alkaline arid land.

## 1. Introduction

Wheat (*Triticum aestivum* L.) is the staple food of Afghanistan [1] and the most widely grown food crop on the planet [2]. One of the issues that has not yet received enough attention from the modern scientific world is the challenging geographical distribution of wheat cultivation in this arid region. Global wheat-producing regions are dominated by calcareous/alkaline soil environments, i.e., major wheat-producing regions × calcareous/alkaline soils [3]. Extensive high-calcium (Ca), high-pH soils are prevalent in the Central Asian and the Afghanistan region due to their arid climate and calcareous parent material. For example, the soil in Afghanistan contains high CaCO_3_ (10–40% or more) and alkalinity (pH 8.0–9.5) [4,5]. Soil Ca is generally considered a nutrient that is representative of an environment where excess toxicity rarely occurs. However, through its antagonism with pH and essential macro- (particularly P fixation as the insoluble “Ca-P” compound) and micronutrients (such as Zn/Fe/Mn), a disruption of cellular processes like respiration, weaker plant standing due to poorly formed root systems, and growth disorders can occur [6,7], hindering global wheat production. Particularly in alkaline soils and calcareous soils (CaCO_3_ > 15%), indirect chlorosis and growth retardation occur. Breeding wheat for calcareous/alkaline tolerance has been generally considered as a means to solve this issue. However, most commercial breeding programs are performed through extensive field-based screening, which is time-consuming and laborious. Selecting specific calcareous/alkali-resilient wheat genotypes that thrive (i.e., evolved/adapted) in such an environment is a promising strategy. Approaches that accelerate this selection process would promote novel and smart strategies in modern wheat breeding.

To survive and adapt to nutrient-limited calcareous and/or alkaline environments, plants employ a crop performance optimization strategy at specific morpho-physiological and molecular levels [5,6,7,8,9]. These include (a) shifting root allocation to maintain a high root-to-shoot ratio; (b) root exudation and root zone pH stabilization; (c) modification of energy transport and the metabolic system; and (d) resistance gene and protein expression. Due to its diverse origins, wheat should have a well-developed strategy to manage calcareous/alkaline environments. However, cultivars that have had calcareous/alkaline-resilient potential might have been excluded due to genetic bottlenecks, and thus, modern wheat shows an average susceptibility. On the other hand, wheat landraces (also known as local varieties) are typically adapted to specific local environments and have developed resilience against a range of environmental challenges [10]. Previously, we studied Kihara Afghan Wheat Landraces (KAWLR) collected by Dr. Hitoshi Kihara and his colleagues during three Botanical Expeditions between 1955 and 1979, organized by the Kyoto University Scientific Expedition [11]. The KAWLR collections, having diverse genetic variations [1,11], showed adaptive responses against drought [12] and excessive CaCO_3_ [5] conditions. These indicate that KAWLR harbor specific genetic resources and traits within the KAWLR collections, making them a critical asset for wheat breeding. In a CaCO_3_ study (Ca^2+^ approximately 35 ppm and pH 8.5), we compared one spring-type KAWLR with Chinese spring and found that KAWLR had greater potential for nutrient acquisition and root growth. Since the KAWLR collections are characterized into spring-type (60%), winter-type (21%), and facultative-type (14%) [13], it is imperative to include greater numbers of them in an experiment. Since the mechanisms of plant response to combined high Ca and high pH are quite an unknown area of study, there are many interesting questions in this field that are still unanswered. Whether KAWLR has evolved specific adaptive morpho-physiological characteristics and environmental adaptation strategies to express effective resistance genes and robustness by adapting to Afghanistan’s soil environment is still unknown. The key players likely rely on a coordinated alteration in their genome functions for metabolic, morpho-physiological adjustment, which is a very complex process.

Proteins, along with their post-translational modifications, are the functional components of the genome, carrying out most cellular processes and playing a crucial role in the endurance and adaptation of plants to different environmental conditions. Proteomic technologies, including gel-based and gel-free methods, allow for the dissection and stress-specific quantification of proteins by providing a detailed picture of protein expression and modification in response to various stress conditions [14]. Because of the disadvantages of gel-based proteomics (labor intensiveness, low sensitivity and reproducibility, and the inability to characterize complete proteomes), gel-free proteomics has become a valuable tool for analyzing the intricate protein makeup of cells or tissues, and thereby understanding stress response mechanisms in wheat [15,16]. While Han et al. [15] reported on proteins associated with sodic-alkaline tolerance in common wheat, there is no readily available report detailing the specific proteins and pathways altered by calcareous-alkaline stress.

By exploiting the semi-hydroponic double cup culture system [5], we challenged ourselves to simulate Ca-rich alkaline soils of arid land, creating different levels of Ca and pH in the bottom cup solutions with Ca(OH)_2_. Wheat responses were evaluated by analyzing the combined effect of Ca and high pH stress on seedling-related traits such as root inhibition, shoot inhibition, chlorophyll content, stomatal conductance, and the pH and elemental status of the bottom-cup solutions. The proteome analysis was then carried out using a tolerant KAWLR, and the essential roles of the regulated proteins were examined to fine-tune the pathways that are involved in the adaptation to the combined high Ca and high pH. The aim of this study is to (i) dissect and understand the molecular basis underlying specific adaptive morpho-physiological characteristics of KAWLR’s genetic resources that allow them to thrive in calcareous-alkaline environments and (ii) thereby contribute to the efficient selection of resilient varieties in a wheat breeding program.

## 2. Results

### 2.1. Generation of Ca Coupled with High pH Screen to Discriminate KAWLR

**Figure 1:**
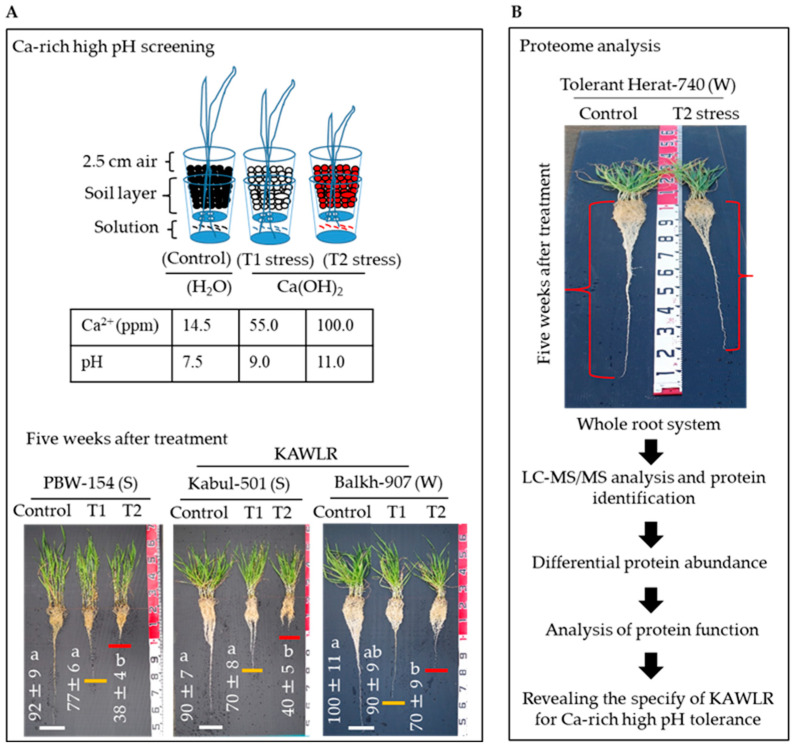
Overall scheme of the Ca-rich, high pH screen, and proteomics-based specific KAWLR selection program for the arid land in Afghanistan. (**A**) (***upper panel***) Ca(OH)_2_-imposed Ca^2+^ coupled with high pH 9.0 (T1 stress) and pH 11.0 (T2 stress) in double chambered cup system. Normal tap water (14.5 ppm Ca^2+^ coupled with pH 7.5) served as a control condition. (***lower panel***) Dose optimization of Ca^2+^ and high pH was determined by cultivating one spring-type modern check (PBW-154), one spring-type KAWLR (Kabul-501), and one winter-type KAWLR (Balkh-907). The whole plant view from one representative cup after harvesting at 5 weeks of T2 treatment, showing root length data (means ± SE; n = 4). Different lowercase letters above the SE indicate statistically significant differences based on one-way ANOVA followed by Tukey’s test (*p* < 0.05). (**B**) Scheme of root proteome study in the representative tolerant winter-type KAWLR genotype Herat-740 grown under control and T2 stress conditions. S, spring-type; W, winter-type; MS, mass spectrometry; LC, nanoliquid chromatography.

**Figure 2:**
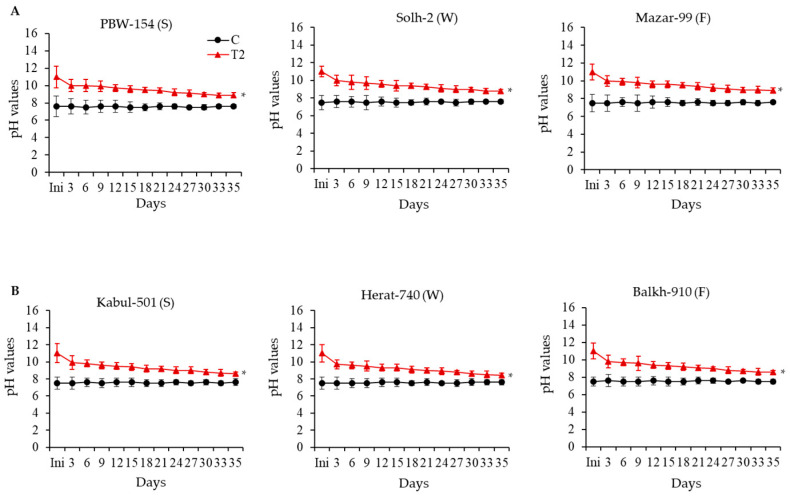
Comparison of pH stabilization among genotypes in response to T2 stress. (**A**) pH stabilization of modern checks PBW-154 (spring-type), Solh-2 (winter-type), and Mazar-99 (facultative-type). (**B**) pH stabilization of KAWLRs Kabul-501 (spring-type), Herat-740 (winter-type), and Balkh-910 (facultative-type). Data was taken every 3 days after T2 treatment for 5 weeks. For each treatment, 4 cups were used. Values represent the mean ± SE (n = 4). * *p* > 0.05. Ini, initial.

To address the wheat culture condition specific to the Ca-rich, high pH, dry soils of Afghanistan, the differences among the plant genotypes were compared between KAWLR and modern check cultivar by exposing their roots in the bottom cup solutions containing various combinations (control, T1 stress, and T2 stress) of Ca^2+^ concentrations (14.5–100 ppm) and pH levels (7.5–11.0) (Figure 1A *upper panel*). The combination of 100 ppm Ca^2+^ and pH 11.0 (T2 stress) was found to be a critical stress condition that significantly impacts the seedling growth of the wheat cultivars of the spring-type modern check PBW-154, spring-type KAWLR Kabul-501, and winter-type KAWLR Herat-907 (Figure 1A *lower panel*; Figure S1). However, the plants were still able to sustain some level of growth for a period of at least five weeks. The unused nutrient level in the bottom cup, root growth, shoot growth, and the physiological response of these three genotypes under the T2 stress condition were found to be clearly differentiable (Table 1; Figure S2 and Figure 2). T2 stress was therefore chosen as a key stress condition to reveal the greatest differences in stress tolerance among plant genotypes.

### 2.2. Status of Unused Nutrients Under T2 Stress

The level of unused Ca^2+^ in the substrate solution was significantly higher for the T2 treatment and markedly varied among genotypes (Table 1). The maximum level of Ca^2+^ was observed in the spring-type modern check cultivars CS and PBW-154, and the minimum in winter-type KAWLRs Baghlan-712 and Herat-740. The levels of unused N^+^ and K^+^ were higher in the T2 stress group compared to the control condition and greatly varied among genotypes and growth habit groups. The highest value of N^+^ was found in CS, and the lowest in the winter-type KAWLR Herat-740. While the K^+^ level in the solution was highest in CS, the lowest level was observed in winter-type KAWLR Balkh-907, followed by other winter-type KAWLRs. Although a relatively lower level of unused P^−^ was detected in the substrate solution of the T2 stress condition compared to the control condition, the P^−^ level was significantly varied among genotypes, with the lowest level found in winter-type KAWLR Herat-740 and the highest in PBW-154.

### 2.3. Root Growth and Plant Responses to T2 Stress

**Figure 3:**
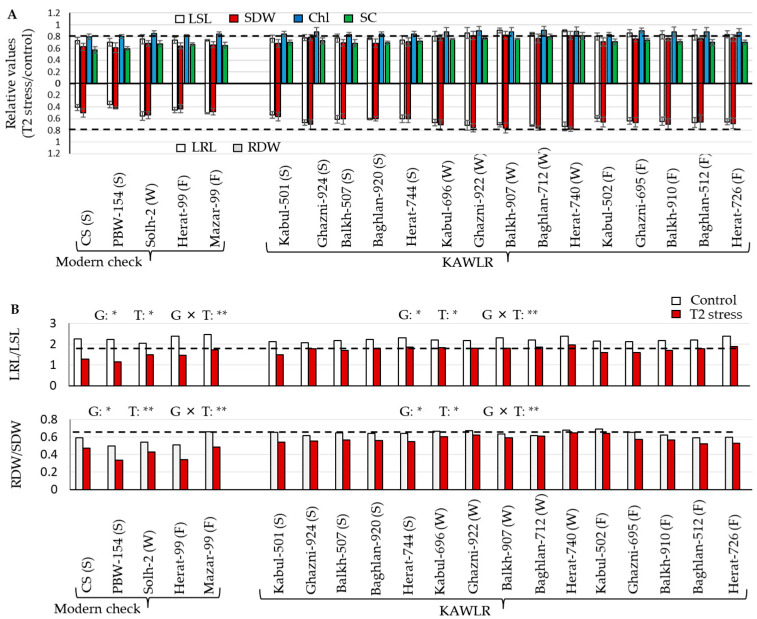
Comparison of root and above-ground trait response of wheat genotypes and growth habits to T2 condition. (**A**) Relative values (T2 stress/control). (***Above graph***), longest shoot length (LSL), shoot dry weight (SDW), chlorophyll (Chl) content, and stomatal conductance (SC); (***below graph***), longest root length (LRL) and root dry weight (RDW). (**B**) LRL/LSL ratio and RDW/SDW ratio. Values are means ± SE (n = 4 for root and shoot traits, n = 10 for Chl and SC). * *p* > 0.05, ** *p* > 0.01. G, genotypes; T, treatments.

Under the T2 stress condition, the growth of wheat seedlings was greatly affected (Figure S2). In order to assess the resilience/tolerance potentials of their root system characteristics, first, we calculated the relative values (T2 stress/control) of their root traits and above-ground traits and evaluated the differences among genotypes in response to T2 stress (Figure 3A). Although the status of the average relative values (T2 stress/control) of these traits was low in this study, there were wide variations across genotypes and growth habits. Overall, the responses in the relative values of the KAWLRs were greater than those of the relative values in the modern check cultivars. When compared to other genotypes and growth habits, only some winter-type KAWLRs showed relative values of ≤0.7, for longest root length (LRL), and ≤0.8, for root dry weight (RDW), under the T2 condition (Figure 3A *below panel*), indicating a relatively vigorous root system, i.e., their root system is large, extensive, and healthy, and spreads quickly and deeply into the soil (Figure S1). The lowest relative values (T2 stress/control) for the longest shoot length (LRL) (Figure 3A *lower panel*) and shoot dry weight (RDW) (Figure 3B *lower panel*) were observed in modern check PBW-154 (0.36 and 0.41), and the highest were observed in Herat-740 (0.73 and 0.78). With respect to the relative values (T2 stress/control) of LSL and SDW (Figure 3A *upper panel*), the overall, as well as the group-wise, response under the T2 condition was similar to that of the relative values observed in the roots. The lowest relative value of LSL was observed in PBW-154 (0.70), and the highest in Balkh-907 (0.91) in response to the T2 condition. The lowest relative value of SDW was found in PBW-154 (0.61), and the highest relative value in Balkh-907 (0.82) and Herat-740 (0.82). As expected, under the T2 stress, the LRL/LSL ratio (Figure 3B *upper panel*) and RDW/SDW ratio (Figure 3B *lower panel*) were markedly higher in winter-type KAWLR compared to modern check, as were other growth habits. The minimum LRL/LSL ratio in response to T2 stress was found in PBW-154 (1.14 and 0.34), and the maximum in Herat-740 (1.96 and 0.65). The lowest RDW/SDW ratio in response to T2 stress was found in CS (0.58), and the highest in Herat-740 (0.81). This indicates that the root systems of the winter-type KAWLR group are prioritized for better resource allocation to achieve specific functions and withstand T2 stress. In this study, chlorophyll (Chl) content did not reduce much under T2 stress compared to stomatal conductance (SC) (Figure S3). Lower relative values in Chl (Figure 3A *upper panel*) and SC (Figure 3A *upper panel*) contents in response to T2 stress were observed in modern check, but these were relatively higher in winter-type KAWLR. The lowest relative value of Chl was observed in CS (0.80), and the highest in Baghlan-712 (0.91). The minimum relative value in SC was found in CS (0.58) and the maximum in Herat-740 (0.81).

In order to reveal the trends of how root traits correlate with other traits, we then compared the correlation of root traits with the above-ground traits, as well as the unused nutrient levels under the control and T2 conditions. As shown in Table 2, LSL, SDW, Chl, and SC showed strong positive correlation with LRL and RDW, especially in response to T2 stress compared to the control condition. A strong negative correlation was found between root traits and the unused levels of Ca^2+^, N^+^, K^+,^ and P^−^ for both root traits; the association was much higher in response to T2 stress than in the control condition.

### 2.4. pH Stabilizing Capacity in Response to T2 Stress

For both modern check and KAWLR, pH stabilizing capacity was measured using one representative genotype from each of the spring, winter, and facultative groups. The pH stabilizing capacity of the wheat genotypes showed variations in response to T2 stress (pH 11.0) (Figure 2). After 5 weeks of T2 treatment initiation, the winter-type KAWLR genotype Herat-740 showed greater pH stabilizing capacity (pH 8.4) compared to that of the modern check PBW-154 (pH8.9).

### 2.5. Responses in Protein Expression of Winter-Type KAWLR Herat-740 to T2 Stress

To gain insight into the molecular-level traits of winter-type KAWLR, a gel-free proteomics analysis in the whole root system of a representative tolerant genotype, Herat-740, was performed. Comparing the protein abundance between the control and T2 plants, out of 828 in total, 12 proteins (approximately 2–3 groups) showed reproducible increases, and 816 (approximately 72–73 groups) proteins showed decreases (*p* < 0.05) (Table S2).

**Figure 4:**
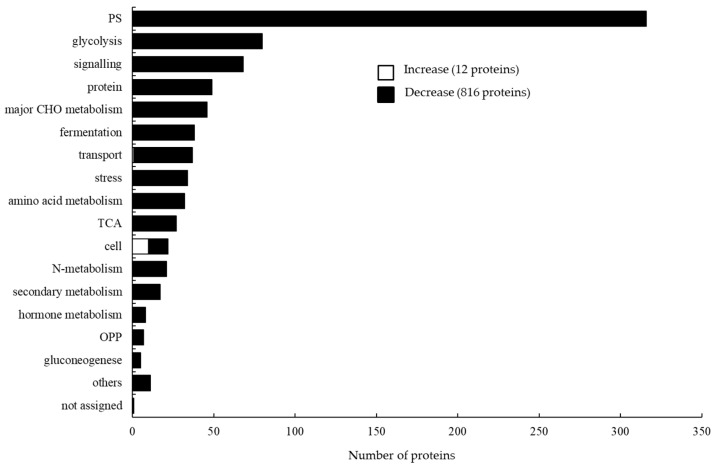
Functional categorization of proteins identified in the root system of the tolerant winter-type KAWLR genotype Herat-740. Eight-day-old wheat seedlings were treated without or with T2 stress for 5 weeks. Proteins were extracted from the whole root system, and significantly changed proteins (*p* < 0.05) were identified using a gel-free proteomic technique. A total of 828 identified proteins were functionally categorized using MapMan bin codes. PS, photosynthesis-related proteins; CHO, carbohydrate; TCA, tricarboxylic acid; OPP, oxidative pentose pathway.

To determine the role of the identified proteins, functional categorization was performed using MapMan bin codes (Figure 4). In response to T2 stress, all the 12 proteins that had increased in abundance were categorized as cell- and transport-related proteins. The proteins that were decreased in abundance were associated with photosynthesis (316 proteins), glycolysis (80 proteins), signaling (68 proteins), protein synthesis (49 proteins), major CHO metabolism (46 proteins), fermentation (38 proteins), transport (36 proteins), stress (34 proteins), amino acid metabolism (32 proteins), TCA (27 proteins), and N-metabolism (21 proteins).

**Figure 5:**
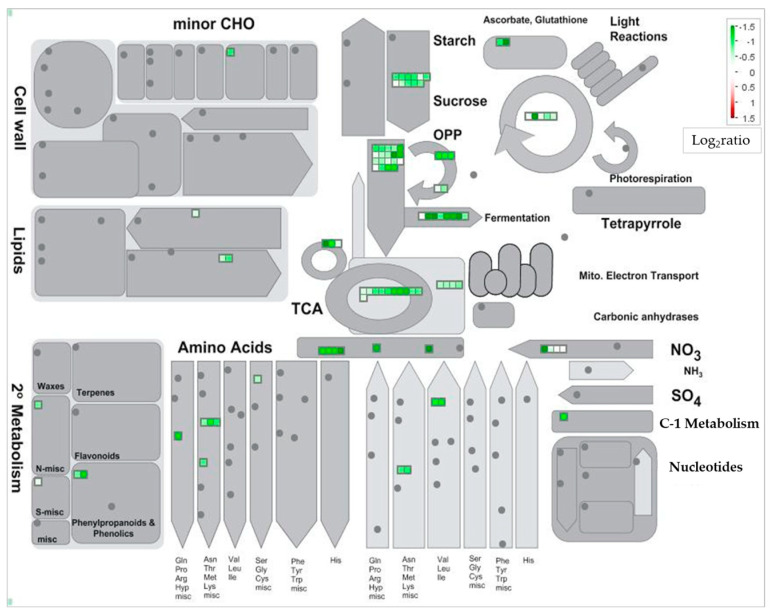
Metabolic pathway analysis of proteins identified in the root system of Herat-740 under T2 stress. The changes in abundance of proteins grouped into functional categories related to primary metabolism were visualized using MapMan software. Significantly changed categories were magnified. Each square and color indicates the log2 ratio value of differentially changed proteins. Red, green, and white colors indicate an increase, decrease, or no change, respectively, in the Log2 ratio values for the T2 treatment compared to the control roots.

To gain an overview of the identified proteins in the tolerant Herat-740 root in detail, significantly changed proteins were mapped on pathway bins in the MapMan software (version 3.6.0RC1). The metabolism overview revealed that most proteins were mapped to glycolysis-, fermentation-, and TCA cycle-related pathways, and most of these proteins had decreased in abundance after 5 weeks of treatment with T2 stress (Figure 5). However, proteins related to cell wall and lipid metabolism pathways remained unchanged against T2 stress.

## 3. Discussion

The co-existence of excessive Ca^2+^ and high pH is the most dominant soil-forming process in arid climates [4,5,6], and these factors, thus, cannot be separated. In the real agricultural world, Ca-rich, high-pH soil stress involves a complex group of stresses composed of excessive Ca, high pH, nutrient deficiency, and aridity. Reproducing this with a single factor is ecologically and agronomically unsound. Our screen-aided proteomic study enabled us to reconstruct this real-world composite-stress environment and comprehensively demonstrate the calcareous-alkalinity-adaptive stress response strategy observed in winter-type Afghan landrace wheat.

It is always challenging to reconstruct a complex stress condition with excessive Ca and high pH, as seen in the real wheat agricultural field, because calcification and alkalization are rarely uniform throughout the soil profile. Once deposited within the soil body, leaching of soluble Ca-rich minerals from the soil surface downwards is minimal because of the absence of percolating water (e.g., rainwater), which, in Afghanistan, is not more than 381 mm annually, with much of this accumulating as winter snow and occasional spring rains [4]. Therefore, in the later phase (February to June) of the wheat growing season (November to June), Ca-rich minerals are mostly accumulated at the soil surface. To cultivate wheat, Afghan farmers usually apply life-supporting commercial irrigation that might dissolve only a small amount of these Ca minerals. In the current study, we improvised a stable semi-hydroponic double-cup wheat culture system [5], with relatively high Ca^2+^ (approximately 100 pm) and high pH (pH 11.0) solutions added into the supplied tap water in the bottom cup. The great advantage of our screen is that it would simulate the natural movement of the Ca^2+^ and pH solution, moving from the bottom cup towards the upper cup containing soil (where germinated seeds were sown) through gradient and/or evaporation via the bottom cup’s opening (Figure 1A; Figure S4). This would mimic most field situations in Afghanistan, where Ca-rich minerals, which are present in the 62 cm~ below the ground [4], evaporate to accumulate over the wheat growing season at the surface. When the wheat seedlings were in a two-leaf stage, and the longest primary root had just begun to pass the upper cup towards the bottom cup through the cup’s holes, T2 stress treatment was applied. As root tips continued to grow, the lower parts of the roots, including the tips, were kept in the solution while the bulk of the upper part of the root system was exposed to pH and Ca-evaporated soil. The two parts of the root system were thus exposed to pH and Ca levels close to those encountered in field situations. In this system, roots encountered the high Ca and high pH solution in a gradual, incremental manner, and the bulk of the root system remained in contact with relatively low calcareous alkalinity. Although our screening system, in terms of its growth response and transport of minerals, differs from the usual hydroponic system, where solutions are continuously washed over entire root systems, this system was close to the real conditions in the field. On the other hand, at the high pH of this system, high Ca concentrations led to the precipitation of insoluble compounds like “Ca-P” in the bottom cup, and thereby reduced the solubility/availability of vital nutrients. We assumed that the high pH, together with Ca^2+^, would create nutritionally deficient conditions for wheat cultivation (Figure S4), and only those genotypes that have the potential to manage these imbalanced nutrient situations would be screened out. When grown in our T2 condition, considerable genetic variability was observed in the root growth of KAWLRs as well as the modern cultivars. The great merit of the T2 screen is that we were able to optimize a relatively higher intensity of Ca and pH in the soils and managed to cultivate KAWLR plants along with modern check on a long-term basis. Thus, the T2 screen allowed us to precisely investigate the distinct genetic variability in most morpho-physiological phases of wheat’s early growth and development. The comparative response of the three wheat genotypes—spring-type modern wheat PBW-154, spring-type KAWLR Kabul-501, and winter-type KAWLR Heart-907, under the T2 stress condition, particularly in relation to root and shoot growth, unused nutrient level in the bottom cup, and physiological traits (Figure 1A; Figure S1 and Haque et al. in this study), suggests a strong correlation between treatment intensity (i.e., control and T2 stress) and resulting changes in the above-ground traits of these three genotypes. This also supports the efficiency of the T2 screen to identify tolerant KAWLRs with specific traits that could perform well in the Ca-rich, high-pH dry soils characteristic of Afghan agricultural landscapes.

Greater root growth and allocation are important in calcareous/alkaline soil and play a pivotal role in early nutrient uptake in wheat grown under low fertility or high pH stress [8,17], thereby impacting plant physiology and development [7,9]. The relatively lower status of Ca^2+^, N^+^, K^+,^ and P^−^ observed in the substrate solution of the T2 stress condition (Table 1) suggests that winter-type KAWLR might have overall better access to these nutrients. Our observation also suggested relatively greater root growth (LRL and RDW) and root/shoot ratios in response to T2 stress, which in turn was reflected in shoot traits (LSL and SDW) and photosynthesis-related traits (Chl and SC) (Figure 3; Figure S2 and Figure 2). While the winter-type KAWR’s altered root system, characterized by its vigor and effective allocation, might be a typical example of a KAWLR under a stress environment [5,12], this winter wheat, which was resistant to high pH, showed higher root length, root dry weight, root/shoot ratio, and nitrogen absorption compared to another winter wheat [8]. The decrease in the Chl content occurred due to reduced absorption of essential nutrients under alkali stress [7,9]. Most studies claim that a shortage of nutrients such as P results in a decrease in net CO_2_ assimilation, which, in most instances, results in a decrease in SC [18]. SC in the leaf tissues is also an indirect measure of deep rooting and water use under dry conditions [12]. The lower reduction in photosynthetic-related traits, particularly Chl content (almost unchanged) in winter-type KAWLR compared to other wheat (Figure 3A *upper panel*), implies not only a higher efficiency in nutrient uptake but also a greater early capacity for photosynthesis and photo-assimilate accumulation under T2 stress. Under low P, unchanged photosynthesis in rice plants is suggested as an indirect indicator of greater carbohydrate allocation to the roots [19]. Root allocation is a key strategy for plants to balance the uptake of water and nutrients by the roots with the capture of energy by leaves [20]. This study found a greater positive correlation of root traits with the above-ground traits and a strong negative correlation with the unused levels of Ca^2+^, N^+^, K^+,^ and P^−^ (in other words, this suggests a greater positive correlation of nutrient absorption with root traits) in response to T2 stress (Table 2). Root traits of KAWLR were positively correlated with their above-ground traits in dry soil [12]. Shi et al. [8] reported that the root traits of winter wheat positively correlated with shoot dry weight and moderately with the N content of the roots and shoots in response to pH alteration. Therefore, the winter-type KAWR’s increased root growth/allocation is suggested as a marker for improved resilience in Ca-rich, high pH soil, potentially leading to robust above-ground (Figure 3 and Table 2), and enhanced acquisition of plant nutrients (Table 1).

Overall, a lower level of unused P^−^ was detected in the T2 solution compared to the control solution, both here and in our previous study [5]. One of the reasons is that Ca^2+^ may bind to P^−^ and precipitate, as we always saw whitish segregation in only the T2 cup (Figure S5). It was difficult to recover 100% P^−^ in our assay method, which does not mean that plants grown in the T2 cup had a higher absorption capacity than plants grown in the control cup. The lower status of unused P^−^ can rather be an indirect indication of the fact that the precipitation of Ca^2+^ with P^−^ might be less prevalent in the winter-type KAWLR (Figure S5). Our results showed that winter-type KAWLR genotype Herat-740 reduces pH better in the T2 cup condition (Figure 2). Winter wheat was reported to moderately decrease the pH of the root [8] in stress (Cd-treated) soil [21]. The pH stabilization can be caused by the higher root exudation potential of wheat plants under stress (alkali) conditions [22]. Some wheat cultivars are shown to acidify their rhizospheres by secreting protons (H^+^) or organic acids [23]. Cieslinski et al. [24] found that the release of organic acids in wheat was cultivar-dependent. Root exudation, especially organic acid release by plants, has been suggested to play a central role in some nutrient acquisition mechanisms operating in calcareous soils [25]. After entering the soil, cations can react with organic acids to form organo-metallic complexes. If these complexes are soluble, they increase the availability of the cation, protect it from precipitation, and also provide a direct route for cation uptake [25]. Therefore, it is speculated that winter-type KAWLR might have a higher capacity to exudate organic acid, regulating pH stabilization, which in turn solubilizes mineral nutrients like Ca-bound phosphorus (Ca–P), and chelates excess Ca to reduce its toxicity, thereby improving the availability of vital nutrients that are less available/soluble under T2 cup conditions.

The central focus of this study was to picture the whole scenario of responsive proteins/pathways and thereby delve into the underlying adaptive strategies that a tolerant winter-type KAWLR genotype applies to withstand a calcareous-alkaline environment. We tried to clarify the responsive proteins and pathways that are maintained (as a marker) and that are given up/sacrificed (as a balancer), especially under prolonged exposure (over 5 weeks) of winter-type KAWLR seedlings to combined excessive Ca and extreme pH (11.0). Continuous exposure of seedlings to calcareous-alkaline environments is an integral part of a wheat plant’s existence under real field conditions. Research dealing with this severe stress condition is rare in proteomic studies. Our results showed a strong decreasing trend in the changed proteins (12 increase/816 decrease) of Herat-740 roots grown under T2 stress (Table S2). Increased proteins were associated with cells and transport, and decreased proteins with pathways related to photosynthesis, N, and amino acid metabolism, protein synthesis, glycolysis, major CHO metabolism, TCA cycle, fermentation, signaling, transport, stress, etc. (Figure 4 and Figure 5). A metabolism overview revealed that the majority of the proteins that were mapped to the central energy metabolism pathways (glycolysis, fermentation, and the TCA cycle) decreased in abundance. Interestingly, proteins related to cell wall and lipid metabolism pathways remained unchanged against T2 stress. This suggests a trend that favors metabolic downshifting to maintain targeted cellular function under T2 stress. The downshifting of genes and proteins is a complex regulatory mechanism where some members might temporarily be inhibited or reduced to redirect resources and fine-tune the plant’s adaptive responses under stress conditions [26]. The photosynthetic rate was reported to decrease in the leaves of wheat under alkali stress (pH 9.08) [27]. The authors postulate that this reduction in photosynthesis can cause reduced production, reducing force and limiting N metabolism, which in turn reduces the production of amino acids and inhibits glycolysis. However, the reason why many of the decreased proteins were related to photosynthesis in the T2 stress-treated root is unknown at present. Roots are capable of developing chloroplasts under light induction [28]. Our double cup system, during culturing plant over 5 weeks of treatment, might have permitted a minimal amount of light, which may have been too low to develop chloroplasts and thereby photosynthesis. However, at present, we do not have data on photosynthetic activity in the system or the induction of chloroplast structures in the roots. We aim to include these valuable points in future studies to clarify the origin of these proteins. In wheat roots, proteins related to carbohydrate metabolism pathways such as glycolysis and the TCA cycle were reported to be decreased in response to sodic-alkaline stress (pH 9.7) [15]. While direct evidence for wheat roots is limited regarding fermentation-related proteins, general principles show that high pH levels reduce glycolysis, affect energy metabolism, and can inhibit key fermentation-related enzymes. Among the 12 proteins, the highest increased protein was MPCP3_ARatH, mitochondrial phosphate carrier protein 3 (MPT3) (Table S2). MPT, which exchanges phosphates between the mitochondrial matrix and cytosol, can be initiated in the nucleus under stressful conditions or changes in nutrient availability [29]. The MPT3 gene was induced by low phosphorus. Transgenic rice overexpressing *McMPT3* or *SlMPT3;1* accumulated more phosphate in shoots under low phosphorus and showed increased tiller numbers and grain yield [30,31]. The induction of the MPT3 protein under certain nutrient conditions, including phosphorus-limited T2 stress (Table 1), is supported by these observations. In this study, several α- and β-tubulin proteins were increased under T2 stress-treated roots (Table S2). The cytosolic proteins α- and β-tubulin assemble into dynamic structures known as microtubules, which are key elements of a plant’s cytoskeleton and have been recognized as important structural elements in cell growth and morphogenesis, and, recently, for their role in regulation and signal transduction for stress adaptation [32]. α- and β-tubulins were reported to be induced at gene and/or protein levels in cold-acclimated wheat crown tissues [33] and salt-adapted *Arabidopsis* root cells [34], respectively. The OST48 (also known as dolichyl-diphosphooligosaccharide-protein glycosyltransferase 48 kDa subunit) is a key, non-catalytic subunit of the endoplasmic reticulum’s oligosaccharyltransferase (OST) complex that facilitates the N-linked glycosylation of proteins, a process vital for their correct folding, quality control, and cellular trafficking. Mutations in the genes encoding the OST48 protein were found to lead to severely compromised phenotypes, including a short root system in rice [35]. Further study on the biological characterization of the above increased protein orthologue(s) in winter-type KAWLR genotype Herat-740 would be helpful to gain insight into the tolerance mechanism of these proteins for T2 stress. Under alkali stress, the plant cell wall is a first line of defense that undergoes remodeling to maintain structural integrity and barrier function (to regulate ion balance) [9,36], while lipid metabolism alters to maintain cell membrane repair/stability, signaling, and overall stress adaptation [37,38]. Environmental stress leads to the loosening of the cell wall, which in turn hinders cell elongation [9]. On the other hand, studies have shown the activation of specific phospholipid and galactolipid metabolism pathways in plants under saline-alkali stress, involving changes in gene expression, to adjust the membrane lipid profile for enhanced tolerance [38]. Thus, Herat-740’s ability to maintain cell wall and lipid metabolism integrity against T2 stress (Figure 5) is a sign of successful homeostasis, with the root organ actively repairing damage rather than being static. Nevertheless, the above results suggest that the winter-type KAWLR adopts a homeostatic stress adaptation strategy that globally downshifts metabolic activity, while selectively maintaining root growth machinery. However, in this study, proteomic analysis was performed using only one representative winter-type KAWLR, and thus could not be evaluated using quantitative experiments. Therefore, the authors aim to pursue future studies using different winter-type genotypes, along with quantitative experiments, to validate and extend the application of this study.

**Figure 6:**
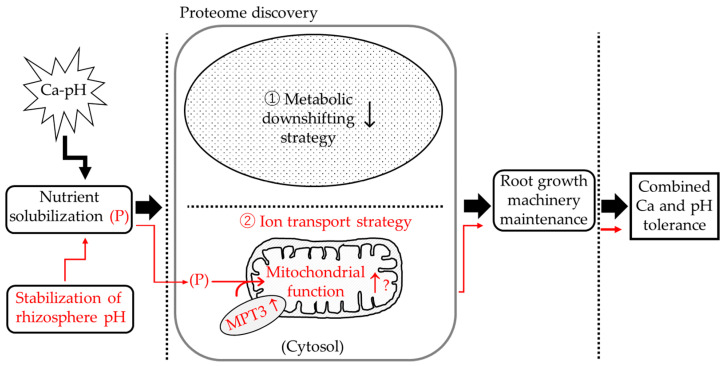
One of the physiological pathways underlying combined excess Ca and extreme pH tolerance in winter-type-KAWLR. The central physiological pathway is shown with solid arrows. The specific P transport pathway is highlighted in red. “↑” and “↓” indicate up- and down-regulation of the relative genes/pathways. For further details, see Discussion.

One of the comprehensive understandings of the underlying physiological pathways of wheat is that greater pH stabilization, which can enhance the solubility of nutrients like the less-available P, and higher expression of MPT3, which can shuttle P into and out of organelles, can thereby facilitate cellular P transport and activate mitochondrial function (Figure 6). The metabolic downshifting strategy and P transport strategy collectively can repair cell damage and maintain root growth to survive combined excess Ca and extreme pH (Figure 6). The P acquisition response, which includes mitochondrial phosphate carrier proteins, is a key physiological strategy of plants in response to P-limiting abiotic stresses [19]. P is also an essential nutrient for diverse physiological processes encompassing cell division, DNA synthesis, phospholipid biosynthesis, and root biomass allocation. Further study is necessary to clarify the above specific hypothesis and to disclose the precise underlying mechanisms in winter-type KAWLR. Overall, our proteomics study not only confirmed the marker proteins and pathways present in these wheat varieties but also enabled us to reveal the strategy of these plants for coping with combined excess Ca and extreme pH, which has not been reported for wheat to the best of our knowledge.

## 4. Materials and Methods

### 4.1. Plant Materials

The current study used a total of 20 distinct wheat varieties (Table S1). Fifteen varieties, comprising 5 spring-, 5 winter-, and 5 facultative-types, were sourced from KAWLRs collected by Dr. Hitoshi Kihara in 1955 [13]. Five were modern check cultivars, consisting of 2 spring, 1 winter, and 2 facultative types. Afghanistan lacks its own locally developed modern wheat varieties; instead, cultivars are sourced from outside the country and are currently being utilized in Afghanistan’s provinces [39]. PBW-154 is a modern spring-type wheat cultivar introduced from India. The modern winter wheat Solh-2 and facultative cultivars Mazar-99 and Herat-99 are from CIMMYT (International Maize and Wheat Improvement Center). Another specific modern wheat variety is Chinese Spring, which is globally used in wheat research and was used here as a modern check. The seeds of all of these wheat varieties were obtained from the Department of Plant Genetic Resource Science, Kihara Institute for Biological Research, Yokohama City University, Japan.

### 4.2. Growing Conditions and Stress Treatment

The semi-hydroponic, double Crimp Crystal cup (PS; LOHACO Co., Ltd., Tokyo, Japan) culture system used here was described previously [5]. Briefly, the upper cup (height 11 cm × upper diameter 8.5 cm) was filled with Volcanic soil (Kaneko Seeds, Maebashi, Japan), then placed into the bottom cup (height 12 cm × upper diameter 8.0 cm and bottom diameter 5.2 cm), which contained water and different levels of Ca and high pH solutions. The bottom of the upper cup contained four holes (about 4 mm in diameter) so that plants growing in the upper cup could absorb the gradient water and nutrients through the bottom cup. Based on the FAO Soils bulletin [4], which conducted a survey on several provinces but not a detailed survey over the different phases of the wheat growing season, the upper limit of alkaline soils of Afghanistan is pH 9.5. The real upper limit of the pH of some alkaline soils may be slightly higher, depending on the crop growing season. To simulate alkaline and Ca-rich soil conditions in Afghanistan, in our previous report [5], we used saturated CaCO_3,_ which gave rise to approximately 35 ppm of Ca^2+^ and 8.5 pH levels at maximum. To improve the condition, in the current study, we have generated two stress conditions with 55 ppm Ca^2+^ coupled with pH 9.0 (T1 stress) and 100 ppm Ca^2+^ coupled with pH 11.0 (T2 stress) using Ca(OH)_2_ in the bottom cup solutions (Figure 1A). Tap water (14.5 ppm Ca^2+^ coupled with pH 7.5) served as the control condition. Ca and pH measurements were performed with an ion/pH meter iM-32P (TOADKK, Tokyo, Japan). Our ultimate goal was to document the genotypic differences in KAWLR. However, although the seedlings of modern checks and some of the spring-type Afghan landrace were much affected by pH 8.5 in our previous study [5] and by pH 9.0 here, the effect of pH 9.0 (T1 stress) was slight and not particularly differentiable in some winter-type KAWLR genotypes. This is also supported by a previous report [8] that found that the inhibition of tolerant winter wheat growth under higher pH (9.0) was not obvious compared to a low-pH (4.0) treatment. Therefore, to evaluate the genetic variations explicitly within KAWLR and between modern cultivars and KAWLR, T2 stress was used for further study.

Plants were cultured as described previously [5]. Briefly, seeds were treated with fungicide GF Benate (Sumitomo Chemical Garden, Tokyo, Japan) for 5–7 h, washed thrice, and then kept under water flow for 30 min. Thirteen healthy seeds per genotype were grown on the upper cup at 2.5 cm and allowed to germinate for 2 nights at room temperature. Plants were cultivated in a growth chamber maintained at a temperature of approximately 22 °C during the day/natural temperature at night, with natural light conditions from October to April at the Kihara Institute for Biological Research, Yokohama city, Kanagawa prefecture, Japan. Seedlings were thinned to 10 per cup, and the cups containing boxes were rotated every day to minimize the position effects on plant growth. T2 stress treatment was imposed on 8-day-old seedlings. Firstly, the bottom solution was removed from each cup and allowed to sit overnight. The next day, 150 mL of each treatment solution was added to the bottom cup. The bottom solution was changed every 3 days. Liquid fertilizer (HYPONeX, Osaka, Japan) was added to the treatment solutions, first to the initial solution, followed by once every 9 days. The culture ball volcanic soil contained pH 6.8–7.1, 55–65% silica (Silicon oxide), 14–20% aluminum oxide, and 5–9% iron oxide (Kaneko Seeds, Japan), but none of the major nutrients. The Hyponex fertilizers contain a pH in tap water of 7.0–7.5, 5% N (NH4 1.25, NO_3_ 1.45), 2% soluble P, 4% soluble K, 0.02% soluble Mg, 0.01% soluble Mn, 0.005% soluble B, a small amount of detergent (like Tween 20) and color. For each treatment (control or stress), 4 cups per genotype were used as replicates. Experiments were repeated twice within the mentioned period.

### 4.3. Measurement of Root Growth and Seedling Related Traits

LRL, RDW, LSL, and SDW were measured at 5 weeks after treatment initiation. To measure the control and T2 stress group samples, all the double-chambered cups for each genotype were first taken out of their boxes. After removing the lower cup, the roots were then carefully separated. The plants were then removed softly from the upper cup, followed by washing with tap water. LRL was measured only for root axes, i.e., not including branch roots. The LSL was measured from the base of the stem to the top of the highest leaf. Shoot and root were oven-dried for 2 d at 80 °C, and then the RDW and SDW were measured. Data were from 2 independent experiments, each with 4 replicated cups per treatment. The Chl concentration and SC were measured in the last leaf at 5 weeks after treatment initiation. The Chl measurements were taken at approximately 4.5 cm from the base of leaves by using a SPAD 502 chlorophyll meter (Konica Minolta, Osaka, Japan). The SC was measured on the abaxial side of the last leaf at 5 weeks after treatment initiation in the noon light period using an SC-1 Leaf Porometer (DECAGON DEVICES, Pullman, WA, USA). For each treatment and cultivar, 10 plants per 1 cup, i.e., 40 plants, were considered.

### 4.4. Elemental and pH Status in the Bottom Cup Solution

The elemental analysis of the substrate solution after 5 weeks from treatment initiation was performed as described previously [5]. The pH stabilization capacity among wheat genotypes was measured in the bottom cup’s solution every 3 days after treatment initiation. Before adding fresh solutions every 3 days from treatment initiation, the bottom cup solution was collected in a blank cup and measured using a pH meter.

### 4.5. Extraction and Preparation of Protein for Mass Spectrometry (MS) Analysis

Whole root systems of three plants grown in four individual cups after 5 weeks of treatment in the T2 and control groups were collected from the winter-type KAWLR genotype Herat-740. For each treatment, protein extraction was performed in biological and technical triplicate. Using 0.5 g of frozen roots, protein extraction was carried out as described previously [40]. Preparation of protein for mass spectrometry (MS) analysis follows the procedure described in our previous report [16]. Briefly, after determining the protein concentration, the detergent was removed from the extracted proteins (100 μg) by chloroform–methanol extraction as follows. Samples (adjusted to 100 μL) were mixed consecutively with methanol (400 μL), chloroform (100 μL), and water (300 μL) and centrifuged at 20,000× *g* for 5 min for phase separation. The upper (aqueous) phase was discarded, and methanol (300 μL) was added to the organic phase. The samples were centrifuged again at 20,000× *g* for 5 min; the supernatants were discarded, and the pellets were dried. Proteins were reduced with 50 mM dithiothreitol for 1 h at 56 °C, alkylated with 50 mM iodoacetamide for 1 h at 37 °C in the dark, and digested with trypsin and lysyl endopeptidase at a 1:100 enzyme/protein ratio for 16 h at 37 °C. The resulting peptides were acidified with formic acid to pH < 3, desalted with a C18-pipette tip, and analyzed by MS.

### 4.6. Nanoliquid Chromatography (LC)-MS/MS Analysis and Protein Identification

LC-MS/MS analysis and protein identification were performed according to the previous report [16]. In brief, peptides were analyzed on a nanospray LTQ XL Orbitrap mass spectrometer (Thermo Fisher Scientific, San Jose, CA, USA) operated in data-dependent acquisition mode with Xcalibur software (version 2.0.7, Thermo Fisher Scientific). Using an Ultimate 3000 nanoLC system (Dionex, Germering, Gemany), peptides in 0.1% formic acid were loaded onto a C18 PepMap trap column (300 µm ID × 5 mm, Dionex), eluted, and separated on a C18 Tip column (75 µm ID × 120 mm nano-HPLC capillary column NTTC-360/75-3; Nikkyo Technos, Tokyo, Japan) in a linear acetonitrile gradient (8–30% in 120 min) in 0.1% formic acid at a flow rate of 200 nL/min. A spray voltage of 1.8 kV was used. Full-scan mass spectra were acquired over a mass range of 400–1500 *m*/*z* with a resolution of 30,000. The lock mass function was used to obtain high mass accuracy. The ten most intense precursor ions were selected for collision-induced fragmentation in a linear ion trap at a normalized collision energy of 35%. Dynamic exclusion was used within 90 s to prevent repetitive selection of the same peptides.

Proteins were identified by the Mascot search engine (version 2.3.0.2, Matrix Science, London, UK) through Mascot Daemon client software (version 2.3.2, Matrix Science) using the protein sequences of green plants, which were downloaded from UniProt (http://www.uniprot.org/uniprot/?query=viridiplantae&fil=reviewed (accessed on 2 December 2016)). The parameters used in the Mascot searches were as follows: cysteine carbamidomethylation was set as a fixed modification, and methionine oxidation was set as a variable modification. Trypsin was specified as the proteolytic enzyme, and one missed cleavage was allowed. Peptide mass tolerance was set at 5 ppm. Fragment mass tolerance was set at 0.5 Da, and peptide charge was set at +2, +3, or +4. An automatic decoy database search was performed as part of the search. Mascot results were filtered with Mascot Percolator to improve the accuracy and sensitivity of peptide identification. The overall reliability of the peptide identifications across all searches was rigorously controlled. Protein hits were considered valid if they were identified by a minimum of two top-ranking peptides, each with a false discovery rate of <1.0%. The Mascot results were exported in XML format for SIEVE (version 2.0, Thermo Fisher Scientific) analysis.

### 4.7. Analysis of Differential Protein Abundance and Function

Analysis of protein abundance was performed by using a label-free quantification package, SIEVE (Thermo Fisher Scientific), to compare the relative abundance of peptides and proteins in the control and treatment groups, as described previously [41]. The thresholds for fold changes in protein quantities in T2 stress vs. control samples were set at a significant difference (*p* < 0.05).

To determine the function of identified proteins, protein ID was transformed into *Arabidopsis* ID, and functional categorization was analyzed using MapMan bin codes [42]. Visualization of protein abundance was performed using MapMan software [42,43].

### 4.8. Statistical Analysis

For each trait, data were summarized by calculating means and standard errors. All the comparisons used analysis of variance (ANOVA) and Tukey’s HSD test as performed with RStudio (ver. 2024.04.1+748). Pearson’s coefficients of correlation among different traits were calculated using SPSS 19.0 statistical software (SPSS, Chicago, IL, USA).

## 5. Conclusions

This study demonstrated that winter-type KAWLR, in a Ca-rich, high pH environment, (1) enhances root allocation shift, (2) stabilizes rhizosphere pH (nutrient solubilization), and (3) maintains a selective proteome response, maintaining root growth machinery. These characteristics proved that winter-type KAWLR displays a prototypical pattern for calcareous-alkaline arid-land-adapted wheat and could potentially be utilized to develop breeding selection markers in early-stage screening for calcareous-alkaline arid-land.

## Figures and Tables

**Table 1:** Comparison of the unused Ca^2+^, N^+^, P^−,^ and K^+^ levels among genotypes in the substrate solutions of control and T2 stress. Values represent mean ± SE (n = 4). Single and double asterisks indicate significant differences at *p* > 0.05 and *p* > 0.01 levels by analysis of variance, respectively. ns, non-significant; S, spring-type; W, winter-type; F, facultative-type.

Genotypes	Treatment	Ca^2+^ (mg/mL)	N^+^ (mg/mL)	K^+^ (mg/mL)	P^−^ (mg/mL)
Chinese spring (S)	Control	27.0 ± 2	4.8 ± 0.3	96.0 ± 8	84.0 ± 9
	T2 stress	67.0 ± 6	47.0 ± 6	125.0 ± 12	74.0 ± 6
PBW-154 (S)	Control	27.0 ± 3	3.8 ± 0.1	97.0 ± 9	84.0 ± 7
	T2 stress	67.0 ± 6	45.0 ± 4	123.0 ± 10	77.0 ± 5
Solh-2 (W)	Control	26.0 ± 4	3.7 ± 0.2	93.0 ± 9	83.0 ± 9
	T2 stress	63.5 ± 8	39.0 ± 6	115.0 ± 10	68.0 ± 7
Mazar-99 (F)	Control	26.5 ± 3	3.8 ± 0.1	95.0 ± 8	83.0 ± 6
	T2 stress	65.0 ± 7	41.0 ± 5	124.0 ± 11	70.0 ± 6
Herat-99 (F)	Control	27.0 ± 4	4.0 ± 0.2	94.0 ± 7	84.0 ± 9
	T2 stress	65.0 ± 6	41.0 ± 7	120.0 ± 10	70.0 ± 6
ANOVA Genotype (G)		ns	ns	ns	ns
Treatment (T)		ns	*	*	*
G × T		**	**	**	**
Kabul-501 (S)	Control	27.0 ± 3	3.0 ± 0.2	93.0 ± 9	82.0 ± 7
	T2 stress	62.0 ± 7	35.0 ± 4	113.0 ± 9	63.0 ± 5
Ghazni-924 (S)	Control	27.0 ± 4	2.4 ± 0.2	93.0 ± 7	81.0 ± 9
	T2 stress	62.0 ± 8	32.0 ± 5	113.0 ± 11	64.0 ± 4
Balkh-507 (S)	Control	27.0 ± 3	2.8 ± 0.3	92.0 ± 8	80.0 ± 8
	T2 stress	62.5 ± 7	33.0 ± 6	111.0 ± 11	63.0 ± 5
Baglan-920 (S)	Control	26.5 ± 4	2.8 ± 0.2	93.0 ± 9	81.0 ± 9
	T2 stress	62.5 ± 6	34.0 ± 7	110.0 ± 11	60.0 ± 6
Herat-744 (S)	Control	27.0 ± 3	2.5 ± 0.2	92.0 ± 9	80.0 ± 6
	T2 stress	62.0 ± 7	35.0 ± 5	109.0 ± 11	60.0 ± 6
Kabul-696 (W)	Control	26.5 ± 2	3.0 ± 0.1	87.0 ± 9	79.0 ± 9
	T2 stress	58.0 ± 5	30.0 ± 6	104.0 ± 8	56.0 ± 6
Ghazni-922 (W)	Control	27.0 ± 2	2.4 ± 0.2	86.0 ± 8	77.0 ± 7
	T2 stress	58.0 ± 8	27.0 ± 5	102.0 ± 8	54.0 ± 3
Balkh-907 (W)	Control	26.5 ± 3	2.8 ± 0.2	84.0 ± 6	79.0 ± 6
	T2 stress	57.5 ± 6	28.0 ± 4	100.0 ± 11	57.0 ± 5
Baghlan-712 (W)	Control	27.0 ± 3	2.9 ± 0.3	86.0 ± 9	80.0 ± 5
	T2 stress	57.0 ± 7	29.0 ± 6	103.0 ± 9	57.0 ± 4
Herat-740 (W)	Control	26.0 ± 2	2.6 ± 0.1	84.0 ± 8	79.0 ± 7
	T2 stress	57.0 ± 6	25.0 ± 4	101.0 ± 7	53.0 ± 6
Kabul-502 (F)	Control	26.5 ± 3	2.6 ± 0.3	94.0 ± 10	83.0 ± 6
	T2 stress	61.0 ± 6	34.0 ± 4	107.0 ± 8	60.0 ± 6
Ghazni-695 (F)	Control	27.0 ± 3	2.9 ± 0.2	94.0 ± 8	83.0 ± 7
	T2 stress	61.5 ± 7	35.0 ± 5	108.0 ± 11	59.0 ± 4
Balkh-910 (F)	Control	26.5 ± 4	2.7 ± 0.3	93.0 ± 9	82.0 ± 8
	T2 stress	60.0 ± 5	34.0 ± 6	105.0 ± 9	58.0 ± 5
Baghlan-512 (F)	Control	27.0 ± 2	3.2 ± 0.1	92.0 ± 9	81.0 ± 9
	T2 stress	61.5 ± 7	33.0 ± 6	107.0 ± 9	57.0 ± 6
Herat-726 (F)	Control	26.5 ± 3	2.8 ± 0.2	92.0 ± 8	82.0 ± 8
	T2 stress	61.0 ± 6	32.0 ± 5	107.0 ± 7	59.0 ± 4
ANOVA Genotype (G)		ns	ns	ns	ns
Treatment (T)		ns	ns	ns	ns
G × T		**	**	**	**

**Table 2:** Correlation of root traits with above-ground traits and substrate solution’s nutrient levels among 20 genotypes under control and T2 conditions. * *p* > 0.05, ** *p* > 0.01 (2-tailed).

Trait ^a^	Treatment	LSL	SDW	Chl	SC	Ca^2+^	N^+^	K^+^	P^−^
LRL	Control	0.64 **	0.43	−0.06	−0.38	−0.18	−0.05	−0.41	−0.33
	T2 stress	0.81 **	0.82 **	0.79 **	0.78 **	−0.94 **	−0.95 **	−0.94 **	−0.93 **
RDW	Control	0.32	0.06	0.34	0.44 *	−0.30	−0.64 **	−0.82 **	−0.76 **
	T2 stress	0.84 **	0.80 **	0.69 **	0.79 **	−0.96 **	−0.93 **	−0.95 **	−0.93 **

^a^ LRL, longest root length; RDW, root dry weight; LSL, longest shoot length; SDW, shoot dry weight; Chl, chlorophyll content; SC, stomatal conductance.

## Data Availability

The original contributions presented in this study are included in the article/Supplementary Materials. Further inquiries can be directed to the corresponding authors.

## Supplementary Materials

The following supporting information can be downloaded at: https://www.mdpi.com/article/10.3390/ijms27010239/s1.
