# Supplementary material for: Analyzing the Specificity of KAWLR Genetic Resources in Afghan Landrace Wheat for Ca-Rich High pH Soil Tolerance Using Proteomics
Source: Int J Mol Sci. 2025 Dec 25;27(1):239. doi: 10.3390/ijms27010239 (PMC12785328; doi:10.3390/ijms27010239)
Supplement: Supplementary file 1 [file ijms-27-00239-s001.zip › ijms-4049440-supplementary.pdf]

**Table S1.** List of the 20 wheat genotypes, with their accessions number, origin/sources, donor and storage year, growth habits.

| Genotypes | Gene bank acc. no. | Origin/Sources | Donor and storage year | Growth habits |
|---|---|---|---|---|
| Chinese spring | | China | NBRB | S |
| PBW-154 | | India | India | S |
| Solh-2 | | CYMMIT | CYMMIT | W |
| Mazar-99 | | CYMMIT | CYMMIT | F |
| Herat-99 | | CYMMIT | CYMMIT | F |
| Kabul-501 | KU-11201 | Kabul | SGK1979 | S |
| Ghazni-924 | KU-7666 | Ghazni | Thomas1967 | S |
| Balkh-507 | KU-11205 | Kunduz | SGK1979 | S |
| Baghlan-920 | KU-7662 | Baghlan | Thomas1967 | S |
| Herat-744 | KU-7453 | Herat | Thomas1967 | W |
| Kabul-696 | KU-3062 | Kabul | KUSE1956 | W |
| Ghazni-922 | KU-7664 | Ghazni | Thomas1967 | W |
| Balkh-907 | KU-7649 | Balkh | Thomas1967 | W |
| Baghlan-712 | KU-3083 | Baghlan | KUSE1956 | W |
| Herat-740 | KU-7449 | Herat | Thomas1967 | W |
| Kabul-502 | KU-11202A | Kabul | SGK1979 | F |
| Ghazni-695 | KU-3060 | Ghazni | KUSE1956 | F |
| Balkh-910 | KU-7652 | Balkh | Thomas1967 | F |
| Baghlan-512 | KU-11209 | Takhar | SGK1979 | F |
| Herat-726 | KU-7434B | Herat | Thomas1967 | F |

The passport data of Kihara Afghan wheat landrace (KAWLR) were taken from NBRP, Komugi (http://shigen.nig.ac.jp/wheat/komugi/strains/queryNbrpAllCategoryAction.do?nbrpStrainGroupCategoryId=5&countryFlg=true&remarksFlg=true) and previous report [13]. The passport data of modern check cultivars was from NBRP, Komugi and previous report [35]. S, spring-type; W, winter-type; F, facultative-type.

**Table S2.** The list of differentially expressed proteins in tolerant winter-type KAWLR genotype Herat-740 root in response to T2 stress condition.

| No. | Protein ID[a] | *Arabidopsis* ID | Description | MP[b] | Ratio[c] | SD | P-value | Function[d] |
|---|---|---|---|---|---|---|---|---|
| | Increase | | | | | | | |
| 1 | SP://Q9FMU6 | at5g14040 | MPCP3_ARatH Mitochondrial phosphate carrier protein 3_ mitochondrial OS=*Arabidopsis thaliana* GN=MPT3 PE=2 SV=1 | 2 | 1.686 | 0.261 | 0.033 | transport |
| 2 | SP://Q9ZRJ4 | at1g50010 | TBA_CHLVU Tubulin alpha chain OS=*Chlorella vulgaris* GN=TUBA PE=3 SV=1 | 11 | 1.487 | 0.104 | 0.041 | cell |
| 3 | SP://P84549 | at5g19780 | TBA_POPEU Tubulin alpha chain (Fragments) OS=*Populus euphratica* PE=1 SV=1 | 3 | 1.477 | 0.114 | 0.009 | cell |
| 4 | SP://B9DGT7 | at1g50010 | TBA2_ARatH Tubulin alpha_2 chain OS=*Arabidopsis thaliana* GN=TUBA2 PE=2 SV=2 | 12 | 1.418 | 0.100 | 0.023 | cell |
| 5 | SP://P29511 | at4g14960 | TBA6_ARatH Tubulin alpha_6 chain OS=*Arabidopsis thaliana* GN=TUBA6 PE=1 SV=1 | 13 | 1.417 | 0.100 | 0.040 | cell |
| 6 | SP://Q6VAG0 | at1g50010 | TBA2_GOSHI Tubulin alpha_2 chain OS=*Gossypium hirsutum* PE=2 SV=1 | 13 | 1.401 | 0.094 | 0.022 | cell |
| 7 | SP://Q6VAF9 | at1g50010 | TBA4_GOSHI Tubulin alpha_4 chain OS=*Gossypium hirsutum* PE=2 SV=1 | 14 | 1.394 | 0.093 | 0.033 | cell |
| 8 | SP://Q9FT36 | at1g50010 | TBA_DAUCA Tubulin alpha chain OS=*Daucus carota* GN=TBA PE=2 SV=1 | 12 | 1.382 | 0.094 | 0.044 | cell |
| 9 | SP://Q9ZRB2 | at5g12250 | TBB1_WHEat Tubulin beta_1 chain OS=*Triticum aestivum* GN=TUBB1 PE=2 SV=1 | 21 | 1.250 | 0.092 | 0.038 | cell |
| 10 | SP://P18026 | at5g12250 | TBB2_MAIZE Tubulin beta_2 chain OS=*Zea mays* GN=TUBB2 PE=2 SV=1 | 18 | 1.247 | 0.094 | 0.030 | cell |
| 11 | SP://Q41785 | at5g12250 | TBB8_MAIZE Tubulin beta_8 chain OS=*Zea mays* GN=TUBB8 PE=2 SV=1 | 21 | 1.243 | 0.091 | 0.041 | cell |
| 12 | SP://Q6ZLK0 | at5g66680 | OST48_ORYSJ Dolichyl_diphosphooligosaccharide__protein glycosyltransferase 48 kDa subunit | 3 | 1.014 | 0.137 | 0.006 | misc |
| | Decrease | | | | | | | |
| 13 | SP://P22200 | at5g08570 | KPYC_SOLTU Pyruvate kinase_ cytosolic isozyme OS=Solanum tuberosum PE=2 SV=1 | 2 | 0.983 | 0.081 | 0.004 | glycolysis |
| 14 | SP://O65595 | at4g26390 | KPYC_ARatH Probable pyruvate kinase_ cytosolic isozyme OS=Arabidopsis thaliana GN=at4g26390 PE=3 SV=1 | 2 | 0.959 | 0.080 | 0.000 | glycolysis |
| 15 | SP://Q42806 | at5g08570 | KPYC_SOYBN Pyruvate kinase_ cytosolic isozyme OS=Glycine max PE=2 SV=1 | 4 | 0.952 | 0.079 | 0.000 | glycolysis |
| 16 | SP://P04078 | at5g37600 | GLNA1_MEDSA Glutamine synthetase cytosolic isozyme OS=Medicago sativa PE=2 SV=1 | 3 | 0.926 | 0.146 | 0.008 | N-metabolism |
| 17 | SP://P14654 | at5g37600 | GLN12_ORYSJ Glutamine synthetase cytosolic isozyme 1_2 OS=Oryza sativa subsp. japonica GN=GLN1_2 PE=1 SV=1 | 3 | 0.926 | 0.146 | 0.008 | N-metabolism |
| 18 | SP://P32289 | at5g37600 | GLNA_VIGAC Glutamine synthetase nodule isozyme OS=Vigna aconitifolia PE=2 SV=1 | 3 | 0.926 | 0.146 | 0.008 | N-metabolism |
| 19 | SP://P38559 | at5g37600 | GLNA1_MAIZE Glutamine synthetase root isozyme 1 OS=Zea mays GN=GLN6 PE=2 SV=1 | 3 | 0.926 | 0.146 | 0.008 | N-metabolism |
| 20 | SP://P38563 | at5g37600 | GLNA5_MAIZE Glutamine synthetase root isozyme 5 OS=Zea mays GN=GS1_5 PE=2 SV=2 | 3 | 0.926 | 0.146 | 0.008 | N-metabolism |
| 21 | SP://P51118 | at5g37600 | GLNA1_VITVI Glutamine synthetase cytosolic isozyme 1 OS=Vitis vinifera GN=GS1_1 PE=2 SV=1 | 3 | 0.926 | 0.146 | 0.008 | N-metabolism |
| 22 | SP://Q56WN1 | at5g37600 | GLN11_ARatH Glutamine synthetase cytosolic isozyme 1_1 OS=Arabidopsis thaliana GN=GLN1_1 PE=1 SV=2 | 3 | 0.926 | 0.146 | 0.008 | N-metabolism |
| 23 | SP://P12424 | at1g66200 | GLNA_NICPL Glutamine synthetase OS=Nicotiana plumbaginifolia PE=2 SV=1 | 4 | 0.914 | 0.143 | 0.022 | N-metabolism |
| 24 | SP://P23712 | at5g37600 | GLNA_LACSA Glutamine synthetase OS=Lactuca sativa PE=2 SV=2 | 4 | 0.914 | 0.143 | 0.022 | N-metabolism |
| 25 | SP://P51119 | at5g37600 | GLNA2_VITVI Glutamine synthetase cytosolic isozyme 2 OS=Vitis vinifera GN=GS1_2 PE=2 SV=1 | 4 | 0.914 | 0.143 | 0.022 | N-metabolism |
| 26 | SP://P38562 | at5g37600 | GLNA4_MAIZE Glutamine synthetase root isozyme 4 OS=Zea mays GN=GLN5 PE=2 SV=1 | 6 | 0.879 | 0.131 | 0.017 | N-metabolism |
| 27 | SP://P05698 | atCg00490 | RBL_HORVU Ribulose bisphosphate carboxylase large chain OS=Hordeum vulgare GN=rbcL PE=3 SV=2 | 6 | 0.828 | 0.148 | 0.027 | PS |
| 28 | SP://Q6K973 | at1g73370 | SUS6_ORYSJ Sucrose synthase 6 OS=Oryza sativa subsp. japonica GN=SUS6 PE=2 SV=1 | 3 | 0.826 | 0.186 | 0.004 | major CHO metabolism |

| | | | | | | | | |
|---|---|---|---|---|---|---|---|---|
| 29 | SP://Q38681 | at1g20260 | VatB1_ACEat V_type proton atPase subunit B 1 OS=Acetabularia acetabulum PE=2 SV=1 | 6 | 0.816 | 0.070 | 0.049 | transport |
| 30 | SP://P22989 | at4g17260 | LDHB_HORVU L_lactate dehydrogenase B (Fragment) OS=Hordeum vulgare PE=1 SV=1 | 7 | 0.815 | 0.112 | 0.016 | fermentation |
| 31 | SP://P48494 | at3g55440 | TPIS_ORYSJ Triosephosphate isomerase_ cytosolic OS=Oryza sativa subsp. japonica GN=TPI PE=1 SV=3 | 4 | 0.812 | 0.131 | 0.000 | glycolysis |
| 32 | SP://Q43432 | at4g38510 | VatB1_GOSHI V_type proton atPase subunit B 1 OS=Gossypium hirsutum PE=2 SV=1 | 15 | 0.801 | 0.060 | 0.038 | transport |
| 33 | SP://P31753 | at2g47110 | RS27A_ASPOF Ubiquitin_40S ribosomal protein S27a (Fragment) OS=Asparagus officinalis PE=2 SV=2 | 3 | 0.801 | 0.257 | 0.039 | protein |
| 34 | SP://P09189 | at5g02500 | HSP7C_PETHY Heat shock cognate 70 kDa protein OS=Petunia hybrida GN=HSP70 PE=2 SV=1 | 19 | 0.801 | 0.092 | 0.033 | stress |
| 35 | SP://Q9SZN1 | at4g38510 | VatB2_ARatH V_type proton atPase subunit B2 OS=Arabidopsis thaliana GN=VHA_B2 PE=1 SV=1 | 16 | 0.801 | 0.060 | 0.050 | transport |
| 36 | SP://Q40078 | at4g38510 | VatB1_HORVU V_type proton atPase subunit B 1 OS=Hordeum vulgare PE=2 SV=1 | 18 | 0.800 | 0.057 | 0.044 | transport |
| 37 | SP://Q40079 | at4g38510 | VatB2_HORVU V_type proton atPase subunit B 2 OS=Hordeum vulgare PE=2 SV=1 | 17 | 0.799 | 0.059 | 0.037 | transport |
| 38 | SP://P11574 | at1g76030 | VatB1_ARatH V_type proton atPase subunit B1 OS=Arabidopsis thaliana GN=VHA_B1 PE=2 SV=2 | 15 | 0.799 | 0.061 | 0.042 | transport |
| 39 | SP://Q8W4E2 | at1g20260 | VatB3_ARatH V_type proton atPase subunit B3 OS=Arabidopsis thaliana GN=VHA_B3 PE=2 SV=1 | 15 | 0.799 | 0.061 | 0.042 | transport |
| 40 | SP://P22954 | at5g02490 | MD37D_ARatH Probable mediator of RNA polymerase II transcription subunit 37c OS=Arabidopsis thaliana GN=MED37D | 17 | 0.796 | 0.086 | 0.029 | protein |
| 41 | SP://Q8RWV0 | at3g60750 | TKTC1_ARatH Transketolase_1_ chloroplastic OS=Arabidopsis thaliana GN=TKL_1 PE=1 SV=1 | 4 | 0.785 | 0.122 | 0.005 | PS |
| 42 | SP://Q9C9C4 | at1g74030 | ENO1_ARatH Enolase 1_ chloroplastic OS=Arabidopsis thaliana GN=ENO1 PE=1 SV=1 | 4 | 0.780 | 0.104 | 0.006 | glycolysis |
| 43 | SP://P35683 | at3g13920 | IF4A1_ORYSJ Eukaryotic initiation factor 4A_1 OS=Oryza sativa subsp. japonica GN=Os06g0701100 PE=2 SV=2 | 13 | 0.777 | 0.085 | 0.045 | protein |
| 44 | SP://Q41741 | at3g13920 | IF4A_MAIZE Eukaryotic initiation factor 4A OS=Zea mays PE=2 SV=1 | 13 | 0.777 | 0.085 | 0.045 | protein |
| 45 | SP://Q6H849 | at5g22300 | NRL4_ORYSJ Bifunctional nitrilase/nitrile hydratase NIT4 OS=Oryza sativa subsp. japonica GN=NIT4 PE=2 SV=1 | 4 | 0.773 | 0.116 | 0.033 | secondary metabolism |
| 46 | SP://P22988 | at4g17260 | LDHA_HORVU L_lactate dehydrogenase A OS=Hordeum vulgare PE=1 SV=1 | 9 | 0.771 | 0.098 | 0.000 | fermentation |
| 47 | SP://P46011 | at5g22300 | NRL4_ARatH Bifunctional nitrilase/nitrile hydratase NIT4 OS=Arabidopsis thaliana GN=NIT4 PE=1 SV=1 | 2 | 0.767 | 0.119 | 0.006 | secondary metabolism |
| 48 | SP://O65719 | at3g09440 | HSP7C_ARatH Heat shock 70 kDa protein 3 OS=Arabidopsis thaliana GN=HSP70_3 PE=1 SV=1 | 15 | 0.767 | 0.093 | 0.022 | stress |
| 49 | SP://P29114 | at1g55020 | LOX1_HORVU Linoleate 9S_lipoxygenase 1 OS=Hordeum vulgare GN=LOX1.1 PE=1 SV=2 | 9 | 0.763 | 0.158 | 0.040 | hormone metabolism |
| 50 | SP://Q9C7X7 | at1g56410 | HSP7N_ARatH Heat shock 70 kDa protein 18 OS=Arabidopsis thaliana GN=HSP70_18 PE=2 SV=1 | 14 | 0.762 | 0.099 | 0.038 | stress |
| 51 | SP://P46226 | at3g55440 | TPIS_SECCE Triosephosphate isomerase_ cytosolic OS=Secale cereale PE=2 SV=3 | 10 | 0.750 | 0.100 | 0.000 | glycolysis |
| 52 | SP://P29357 | at3g12580 | HSP7E_SPIOL Chloroplast envelope membrane 70 kDa heat shock_related protein OS=Spinacia oleracea GN=SCE70 PE=1 SV=3 | 14 | 0.750 | 0.091 | 0.007 | stress |
| 53 | SP://P27322 | at5g02500 | HSP72_SOLLC Heat shock cognate 70 kDa protein 2 OS=Solanum lycopersicum GN=HSC_2 PE=2 SV=1 | 21 | 0.747 | 0.092 | 0.028 | stress |
| 54 | SP://O82399 | at2g22780 | MDHG2_ARatH Probable malate dehydrogenase_ glyoxysomal OS=Arabidopsis thaliana GN=at2g22780 PE=2 SV=1 | 2 | 0.746 | 0.154 | 0.041 | gluconeogenese |
| 55 | SP://Q9FLH8 | at5g51830 | SCRK7_ARatH Probable fructokinase_7 OS=Arabidopsis thaliana GN=at5g51830 PE=1 SV=1 | 2 | 0.742 | 0.248 | 0.032 | major CHO metabolism |
| 56 | SP://Q42965 | at5g22300 | NRL4A_TOBAC Bifunctional nitrilase/nitrile hydratase NIT4A OS=Nicotiana tabacum GN=NIT4A PE=2 SV=1 | 2 | 0.742 | 0.146 | 0.030 | secondary metabolism |
| 57 | SP://Q42966 | at5g22300 | NRL4B_TOBAC Bifunctional nitrilase/nitrile hydratase NIT4B OS=Nicotiana tabacum GN=NIT4B PE=2 SV=1 | 2 | 0.742 | 0.146 | 0.030 | secondary metabolism |
| 58 | SP://P83373 | at1g53240 | MDHM_FRAAN Malate dehydrogenase_ mitochondrial OS=Fragaria ananassa GN=MMDHI PE=1 SV=1 | 3 | 0.742 | 0.121 | 0.022 | TCA |
| 59 | SP://P12863 | at3g55440 | TPIS_MAIZE Triosephosphate isomerase_ cytosolic OS=Zea mays PE=3 SV=3 | 2 | 0.742 | 0.183 | 0.042 | glycolysis |
| 60 | SP://P22953 | at5g02500 | MD37E_ARatH Probable mediator of RNA polymerase II transcription subunit 37e OS=Arabidopsis thaliana GN=MED37E | 17 | 0.741 | 0.093 | 0.048 | stress |
| 61 | SP://P52904 | at5g50850 | ODPB_PEA Pyruvate dehydrogenase E1 component subunit beta_ mitochondrial OS=Pisum sativum PE=2 SV=1 | 2 | 0.740 | 0.126 | 0.018 | TCA |
| 62 | SP://P13564 | at5g35630 | GLNA2_HORVU Glutamine synthetase leaf isozyme_ chloroplastic OS=Hordeum vulgare PE=2 SV=2 | 4 | 0.740 | 0.147 | 0.023 | N-metabolism |

| | | | | | | | | |
|---|---|---|---|---|---|---|---|---|
| 63 | SP://Q0J0H4 | at5g50850 | ODPB2_ORYSJ Pyruvate dehydrogenase E1 component subunit beta_2_ mitochondrial OS=Oryza sativa subsp. japonica | 3 | 0.739 | 0.125 | 0.045 | TCA |
| 64 | SP://Q42972 | at2g22780 | MDHG_ORYSJ Malate dehydrogenase_ glyoxysomal OS=Oryza sativa subsp. japonica GN=Os12g0632700 PE=1 SV=3 | 2 | 0.733 | 0.133 | 0.009 | gluconeogenese |
| 65 | SP://Q949P2 | at5g10540 | COPDA_ARatH Probable cytosolic oligopeptidase A OS=Arabidopsis thaliana GN=CYOP PE=1 SV=1 | 2 | 0.731 | 0.182 | 0.036 | protein |
| 66 | SP://Q94AM1 | at5g65620 | OOPDA_ARatH Organellar oligopeptidase A_ chloroplastic/mitochondrial OS=Arabidopsis thaliana GN=OOP PE=1 SV=1 | 2 | 0.731 | 0.182 | 0.036 | protein |
| 67 | SP://Q9LTX9 | at5g49910 | HSP7G_ARatH Heat shock 70 kDa protein 7_ chloroplastic OS=Arabidopsis thaliana GN=HSP70_7 PE=2 SV=1 | 7 | 0.730 | 0.111 | 0.003 | stress |
| 68 | SP://P28769 | at3g20050 | TCPA_ARatH T_complex protein 1 subunit alpha OS=Arabidopsis thaliana GN=CCT1 PE=1 SV=1 | 2 | 0.725 | 0.242 | 0.046 | protein |
| 69 | SP://Q32223 | atCg00490 | RBL_EPHTW Ribulose bisphosphate carboxylase large chain (Fragment) OS=Ephedra tweediana GN=rbcL PE=3 SV=1 | 3 | 0.724 | 0.186 | 0.005 | PS |
| 70 | SP://B9DGD6 | at5g36880 | ACS_ARatH Acetyl_coenzyme A synthetase_ chloroplastic/glyoxysomal OS=Arabidopsis thaliana GN=ACS PE=1 SV=1 | 3 | 0.719 | 0.173 | 0.046 | lipid metabolism |
| 71 | SP://Q08080 | at4g24280 | HSP7S_SPIOL Stromal 70 kDa heat shock_related protein_ chloroplastic (Fragment) OS=Spinacia oleracea | 5 | 0.719 | 0.094 | 0.000 | stress |
| 72 | SP://P08281 | at5g35630 | GLNA2_PEA Glutamine synthetase leaf isozyme_ chloroplastic OS=Pisum sativum GN=GS2 PE=2 SV=2 | 3 | 0.718 | 0.151 | 0.009 | N-metabolism |
| 73 | SP://P14655 | at5g35630 | GLNA2_ORYSJ Glutamine synthetase_ chloroplastic OS=Oryza sativa subsp. japonica GN=GLN2 PE=1 SV=1 | 3 | 0.718 | 0.151 | 0.009 | N-metabolism |
| 74 | SP://P15102 | at5g35630 | GLNA4_PHAVU Glutamine synthetase leaf isozyme_ chloroplastic OS=Phaseolus vulgaris PE=2 SV=1 | 3 | 0.718 | 0.151 | 0.009 | N-metabolism |
| 75 | SP://P25462 | at5g35630 | GLNAC_MAIZE Glutamine synthetase_ chloroplastic OS=Zea mays GN=GLN2 PE=2 SV=1 | 3 | 0.718 | 0.151 | 0.009 | N-metabolism |
| 76 | SP://Q9XQ94 | at5g35630 | GLNA2_MEDSA Glutamine synthetase leaf isozyme_ chloroplastic OS=Medicago sativa GN=GS2 PE=2 SV=1 | 3 | 0.718 | 0.151 | 0.009 | N-metabolism |
| 77 | SP://Q0J035 | at4g32180 | PANK2_ORYSJ Pantothenate kinase 2 OS=Oryza sativa subsp. japonica GN=Os09g0533100 PE=2 SV=2 | 4 | 0.718 | 0.135 | 0.018 | vitamine metabolism |
| 78 | SP://P46524 | at1g20440 | CO410_WHEat Dehydrin COR410 OS=Triticum aestivum GN=COR410 PE=2 SV=1 | 5 | 0.718 | 0.281 | 0.032 | stress |
| 79 | SP://P31180 | atCg00490 | RBL_BUXSE Ribulose bisphosphate carboxylase large chain (Fragment) OS=Buxus sempervirens GN=rbcL PE=3 SV=1 | 2 | 0.716 | 0.197 | 0.031 | PS |
| 80 | SP://P31195 | atCg00490 | RBL_MERAN Ribulose bisphosphate carboxylase large chain (Fragment) OS=Mercurialis annua GN=rbcL PE=3 SV=1 | 2 | 0.716 | 0.197 | 0.031 | PS |
| 81 | SP://P31200 | atCg00490 | RBL_ROSDA Ribulose bisphosphate carboxylase large chain (Fragment) OS=Rosa damascena GN=rbcL PE=3 SV=1 | 2 | 0.716 | 0.197 | 0.031 | PS |
| 82 | SP://P36489 | atCg00490 | RBL_STRNX Ribulose bisphosphate carboxylase large chain (Fragment) OS=Strychnos nux_vomica GN=rbcL PE=3 SV=1 | 2 | 0.716 | 0.197 | 0.031 | PS |
| 83 | SP://P51994 | atCg00490 | RBL_BAMML Ribulose bisphosphate carboxylase large chain (Fragment) OS=Bambusa multiplex GN=rbcL PE=3 SV=1 | 2 | 0.716 | 0.197 | 0.031 | PS |
| 84 | SP://P69567 | atCg00490 | RBL_EUPCH Ribulose bisphosphate carboxylase large chain (Fragment) OS=Euphorbia characias GN=rbcL PE=3 SV=1 | 2 | 0.716 | 0.197 | 0.031 | PS |
| 85 | SP://Q31750 | atCg00490 | RBL_CALCA Ribulose bisphosphate carboxylase large chain (Fragment) OS=Calycophyllum candidissimum GN=rbcL | 2 | 0.716 | 0.197 | 0.031 | PS |
| 86 | SP://Q31886 | atCg00490 | RBL_BOUTR Ribulose bisphosphate carboxylase large chain OS=Bouvardia ternifolia GN=rbcL PE=3 SV=2 | 2 | 0.716 | 0.197 | 0.031 | PS |
| 87 | SP://A0ZZ43 | atCg00490 | RBL_GOSBA Ribulose bisphosphate carboxylase large chain OS=Gossypium barbadense GN=rbcL PE=3 SV=1 | 2 | 0.710 | 0.175 | 0.032 | PS |
| 88 | SP://A4GYR8 | atCg00490 | RBL_POPTR Ribulose bisphosphate carboxylase large chain OS=Populus trichocarpa GN=rbcL PE=3 SV=1 | 2 | 0.710 | 0.175 | 0.032 | PS |
| 89 | SP://A6H5I5 | atCg00490 | RBL_CYCTA Ribulose bisphosphate carboxylase large chain OS=Cycas taitungensis GN=rbcL PE=3 SV=1 | 2 | 0.710 | 0.175 | 0.032 | PS |
| 90 | SP://A6MML5 | atCg00490 | RBL_DIOEL Ribulose bisphosphate carboxylase large chain OS=Dioscorea elephantipes GN=rbcL PE=3 SV=1 | 2 | 0.710 | 0.175 | 0.032 | PS |
| 91 | SP://A9L9A4 | atCg00490 | RBL_LEMMI Ribulose bisphosphate carboxylase large chain OS=Lemna minor GN=rbcL PE=3 SV=1 | 2 | 0.710 | 0.175 | 0.032 | PS |
| 92 | SP://B1NWF8 | atCg00490 | RBL_MANES Ribulose bisphosphate carboxylase large chain OS=Manihot esculenta GN=rbcL PE=3 SV=1 | 2 | 0.710 | 0.175 | 0.032 | PS |
| 93 | SP://B2X1Y2 | atCg00490 | RBL_OEDCA Ribulose bisphosphate carboxylase large chain OS=Oedogonium cardiacum GN=rbcL PE=3 SV=1 | 2 | 0.710 | 0.175 | 0.032 | PS |
| 94 | SP://O19872 | atCg00490 | RBL_BIXOR Ribulose bisphosphate carboxylase large chain (Fragment) OS=Bixa orellana GN=rbcL PE=3 SV=2 | 2 | 0.710 | 0.175 | 0.032 | PS |
| 95 | SP://O62964 | atCg00490 | RBL_VIGUN Ribulose bisphosphate carboxylase large chain OS=Vigna unguiculata GN=rbcL PE=3 SV=1 | 2 | 0.710 | 0.175 | 0.032 | PS |
| 96 | SP://O78258 | atCg00490 | RBL_ABIFI Ribulose bisphosphate carboxylase large chain (Fragment) OS=Abies firma GN=rbcL PE=3 SV=1 | 2 | 0.710 | 0.175 | 0.032 | PS |

| | | | | | | | | |
|---|---|---|---|---|---|---|---|---|
| 97 | SP://O78259 | atCg00490 | RBL_ABIHO Ribulose bisphosphate carboxylase large chain (Fragment) OS=Abies homolepis GN=rbcL PE=3 SV=1 | 2 | 0.710 | 0.175 | 0.032 | PS |
| 98 | SP://O78260 | atCg00490 | RBL_ABIVE Ribulose bisphosphate carboxylase large chain (Fragment) OS=Abies veitchii GN=rbcL PE=3 SV=1 | 2 | 0.710 | 0.175 | 0.032 | PS |
| 99 | SP://O99001 | atCg00490 | RBL_BEAGR Ribulose bisphosphate carboxylase large chain (Fragment) OS=Beaumontia grandiflora GN=rbcL PE=3 SV=1 | 2 | 0.710 | 0.175 | 0.032 | PS |
| 100 | SP://P04992 | atCg00490 | RBL_PETHY Ribulose bisphosphate carboxylase large chain OS=Petunia hybrida GN=rbcL PE=1 SV=1 | 2 | 0.710 | 0.175 | 0.032 | PS |
| 101 | SP://P06292 | atCg00490 | RBL_MARPO Ribulose bisphosphate carboxylase large chain OS=Marchantia polymorpha GN=rbcL PE=1 SV=1 | 2 | 0.710 | 0.175 | 0.032 | PS |
| 102 | SP://P11421 | atCg00490 | RBL_NICAC Ribulose bisphosphate carboxylase large chain OS=Nicotiana acuminata GN=rbcL PE=3 SV=1 | 2 | 0.710 | 0.175 | 0.032 | PS |
| 103 | SP://P11422 | atCg00490 | RBL_NICOT Ribulose bisphosphate carboxylase large chain OS=Nicotiana otophora GN=rbcL PE=3 SV=1 | 2 | 0.710 | 0.175 | 0.032 | PS |
| 104 | SP://P14958 | atCg00490 | RBL_GOSHI Ribulose bisphosphate carboxylase large chain OS=Gossypium hirsutum GN=rbcL PE=3 SV=2 | 2 | 0.710 | 0.175 | 0.032 | PS |
| 105 | SP://P24675 | atCg00490 | RBL_PINED Ribulose bisphosphate carboxylase large chain OS=Pinus edulis GN=rbcL PE=3 SV=1 | 2 | 0.710 | 0.175 | 0.032 | PS |
| 106 | SP://P24678 | atCg00490 | RBL_PINPI Ribulose bisphosphate carboxylase large chain OS=Pinus pinea GN=rbcL PE=3 SV=1 | 2 | 0.710 | 0.175 | 0.032 | PS |
| 107 | SP://P24680 | atCg00490 | RBL_AFRGR Ribulose bisphosphate carboxylase large chain OS=Afrocarpus gracilior GN=rbcL PE=3 SV=1 | 2 | 0.710 | 0.175 | 0.032 | PS |
| 108 | SP://P25079 | atCg00490 | RBL_SOLTU Ribulose bisphosphate carboxylase large chain OS=Solanum tuberosum GN=rbcL PE=1 SV=2 | 2 | 0.710 | 0.175 | 0.032 | PS |
| 109 | SP://P25829 | atCg00490 | RBL_CALUS Ribulose bisphosphate carboxylase large chain (Fragment) OS=Calamus usitatus GN=rbcL PE=3 SV=1 | 2 | 0.710 | 0.175 | 0.032 | PS |
| 110 | SP://P25836 | atCg00490 | RBL_SERRE Ribulose bisphosphate carboxylase large chain (Fragment) OS=Serenoa repens GN=rbcL PE=3 SV=1 | 2 | 0.710 | 0.175 | 0.032 | PS |
| 111 | SP://P26960 | atCg00490 | RBL_KETDA Ribulose bisphosphate carboxylase large chain OS=Keteleeria davidiana GN=rbcL PE=3 SV=2 | 2 | 0.710 | 0.175 | 0.032 | PS |
| 112 | SP://P26962 | atCg00490 | RBL_PINBA Ribulose bisphosphate carboxylase large chain OS=Pinus balfouriana GN=rbcL PE=3 SV=1 | 2 | 0.710 | 0.175 | 0.032 | PS |
| 113 | SP://P26963 | atCg00490 | RBL_PINKR Ribulose bisphosphate carboxylase large chain OS=Pinus krempfii GN=rbcL PE=3 SV=1 | 2 | 0.710 | 0.175 | 0.032 | PS |
| 114 | SP://P27065 | atCg00490 | RBL_SOLLC Ribulose bisphosphate carboxylase large chain OS=Solanum lycopersicum GN=rbcL PE=1 SV=4 | 2 | 0.710 | 0.175 | 0.032 | PS |
| 115 | SP://P28259 | atCg00490 | RBL_DRYSU Ribulose bisphosphate carboxylase large chain (Fragment) OS=Drymophloeus subdistichus GN=rbcL PE=3 SV=1 | 2 | 0.710 | 0.175 | 0.032 | PS |
| 116 | SP://P28261 | atCg00490 | RBL_NYPFR Ribulose bisphosphate carboxylase large chain (Fragment) OS=Nypa fruticans GN=rbcL PE=3 SV=1 | 2 | 0.710 | 0.175 | 0.032 | PS |
| 117 | SP://P28262 | atCg00490 | RBL_PHORE Ribulose bisphosphate carboxylase large chain (Fragment) OS=Phoenix reclinata GN=rbcL PE=3 SV=1 | 2 | 0.710 | 0.175 | 0.032 | PS |
| 118 | SP://P28376 | atCg00490 | RBL_ACESA Ribulose bisphosphate carboxylase large chain (Fragment) OS=Acer saccharum GN=rbcL PE=3 SV=1 | 2 | 0.710 | 0.175 | 0.032 | PS |
| 119 | SP://P28377 | atCg00490 | RBL_ACTCH Ribulose bisphosphate carboxylase large chain (Fragment) OS=Actinidia chinensis GN=rbcL PE=3 SV=1 | 2 | 0.710 | 0.175 | 0.032 | PS |
| 120 | SP://P28380 | atCg00490 | RBL_APIGR Ribulose bisphosphate carboxylase large chain (Fragment) OS=Apium graveolens GN=rbcL PE=3 SV=1 | 2 | 0.710 | 0.175 | 0.032 | PS |
| 121 | SP://P28385 | atCg00490 | RBL_BURIN Ribulose bisphosphate carboxylase large chain (Fragment) OS=Bursera inaguensis GN=rbcL PE=3 SV=1 | 2 | 0.710 | 0.175 | 0.032 | PS |
| 122 | SP://P28389 | atCg00490 | RBL_CASEQ Ribulose bisphosphate carboxylase large chain (Fragment) OS=Casuarina equisetifolia GN=rbcL PE=3 SV=1 | 2 | 0.710 | 0.175 | 0.032 | PS |
| 123 | SP://P28393 | atCg00490 | RBL_CORCA Ribulose bisphosphate carboxylase large chain (Fragment) OS=Cornus canadensis GN=rbcL PE=3 SV=3 | 2 | 0.710 | 0.175 | 0.032 | PS |
| 124 | SP://P28397 | atCg00490 | RBL_DAPSP Ribulose bisphosphate carboxylase large chain (Fragment) OS=Daphniphyllum sp. GN=rbcL PE=3 SV=1 | 2 | 0.710 | 0.175 | 0.032 | PS |
| 125 | SP://P28398 | atCg00490 | RBL_DARCA Ribulose bisphosphate carboxylase large chain (Fragment) OS=Darlingtonia californica GN=rbcL PE=3 SV=2 | 2 | 0.710 | 0.175 | 0.032 | PS |
| 126 | SP://P28402 | atCg00490 | RBL_DRIWI Ribulose bisphosphate carboxylase large chain (Fragment) OS=Drimys winteri GN=rbcL PE=3 SV=2 | 2 | 0.710 | 0.175 | 0.032 | PS |
| 127 | SP://P28415 | atCg00490 | RBL_EUCUL Ribulose bisphosphate carboxylase large chain (Fragment) OS=Eucommia ulmoides GN=rbcL PE=3 SV=2 | 2 | 0.710 | 0.175 | 0.032 | PS |
| 128 | SP://P28417 | atCg00490 | RBL_GERGR Ribulose bisphosphate carboxylase large chain (Fragment) OS=Geranium grandiflorum GN=rbcL PE=3 SV=1 | 2 | 0.710 | 0.175 | 0.032 | PS |
| 129 | SP://P28421 | atCg00490 | RBL_HEDHE Ribulose bisphosphate carboxylase large chain (Fragment) OS=Hedera helix GN=rbcL PE=3 SV=1 | 2 | 0.710 | 0.175 | 0.032 | PS |
| 130 | SP://P28424 | atCg00490 | RBL_HUMBA Ribulose bisphosphate carboxylase large chain (Fragment) OS=Humiria balsamifera GN=rbcL PE=3 SV=1 | 2 | 0.710 | 0.175 | 0.032 | PS |

| | | | | | | | | |
|---|---|---|---|---|---|---|---|---|
| 131 | SP://P28427 | atCg00490 | RBL_JASSS Ribulose bisphosphate carboxylase large chain (Fragment) OS=Jasminum simplicifolium subsp. | 2 | 0.710 | 0.175 | 0.032 | PS |
| 132 | SP://P28437 | atCg00490 | RBL_PARFI Ribulose bisphosphate carboxylase large chain (Fragment) OS=Parnassia fimbriata GN=rbcL PE=3 SV=2 | 2 | 0.710 | 0.175 | 0.032 | PS |
| 133 | SP://P28438 | atCg00490 | RBL_PASQU Ribulose bisphosphate carboxylase large chain (Fragment) OS=Passiflora quadrangularis GN=rbcL PE=3 SV=1 | 2 | 0.710 | 0.175 | 0.032 | PS |
| 134 | SP://P28439 | atCg00490 | RBL_PELHO Ribulose bisphosphate carboxylase large chain OS=Pelargonium hortorum GN=rbcL_A PE=3 SV=2 | 2 | 0.710 | 0.175 | 0.032 | PS |
| 135 | SP://P28443 | atCg00490 | RBL_POLCR Ribulose bisphosphate carboxylase large chain (Fragment) OS=Polygala cruciata GN=rbcL PE=3 SV=2 | 2 | 0.710 | 0.175 | 0.032 | PS |
| 136 | SP://P28448 | atCg00490 | RBL_RORGO Ribulose bisphosphate carboxylase large chain (Fragment) OS=Roridula gorgonias GN=rbcL PE=3 SV=1 | 2 | 0.710 | 0.175 | 0.032 | PS |
| 137 | SP://P28455 | atCg00490 | RBL_SEDRU Ribulose bisphosphate carboxylase large chain (Fragment) OS=Sedum rubrotinctum GN=rbcL PE=3 SV=1 | 2 | 0.710 | 0.175 | 0.032 | PS |
| 138 | SP://P28458 | atCg00490 | RBL_TROAR Ribulose bisphosphate carboxylase large chain (Fragment) OS=Trochodendron aralioides GN=rbcL PE=3 SV=1 | 2 | 0.710 | 0.175 | 0.032 | PS |
| 139 | SP://P31189 | atCg00490 | RBL_HIPRI Ribulose bisphosphate carboxylase large chain (Fragment) OS=Hippocratea richardiana GN=rbcL PE=3 SV=2 | 2 | 0.710 | 0.175 | 0.032 | PS |
| 140 | SP://P31202 | atCg00490 | RBL_SALPL Ribulose bisphosphate carboxylase large chain (Fragment) OS=Salacia pallescens GN=rbcL PE=3 SV=2 | 2 | 0.710 | 0.175 | 0.032 | PS |
| 141 | SP://P34767 | atCg00490 | RBL_ALIPL Ribulose bisphosphate carboxylase large chain (Fragment) OS=Alisma plantago_aquatica GN=rbcL PE=3 SV=1 | 2 | 0.710 | 0.175 | 0.032 | PS |
| 142 | SP://P41621 | atCg00490 | RBL_PINTH Ribulose bisphosphate carboxylase large chain OS=Pinus thunbergii GN=rbcL PE=3 SV=1 | 2 | 0.710 | 0.175 | 0.032 | PS |
| 143 | SP://P43228 | atCg00490 | RBL_CIBBA Ribulose bisphosphate carboxylase large chain (Fragment) OS=Cibotium barometz GN=rbcL PE=3 SV=1 | 2 | 0.710 | 0.175 | 0.032 | PS |
| 144 | SP://P48688 | atCg00490 | RBL_CARPA Ribulose bisphosphate carboxylase large chain OS=Carica papaya GN=rbcL PE=3 SV=2 | 2 | 0.710 | 0.175 | 0.032 | PS |
| 145 | SP://P48696 | atCg00490 | RBL_CRYJA Ribulose bisphosphate carboxylase large chain OS=Cryptomeria japonica GN=rbcL PE=3 SV=2 | 2 | 0.710 | 0.175 | 0.032 | PS |
| 146 | SP://P48698 | atCg00490 | RBL_DatST Ribulose bisphosphate carboxylase large chain (Fragment) OS=Datura stramonium GN=rbcL PE=3 SV=1 | 2 | 0.710 | 0.175 | 0.032 | PS |
| 147 | SP://P48704 | atCg00490 | RBL_GINBI Ribulose bisphosphate carboxylase large chain (Fragment) OS=Ginkgo biloba GN=rbcL PE=3 SV=1 | 2 | 0.710 | 0.175 | 0.032 | PS |
| 148 | SP://P48709 | atCg00490 | RBL_NICDE Ribulose bisphosphate carboxylase large chain OS=Nicotiana debneyi GN=rbcL PE=3 SV=1 | 2 | 0.710 | 0.175 | 0.032 | PS |
| 149 | SP://P48713 | atCg00490 | RBL_POPTM Ribulose bisphosphate carboxylase large chain OS=Populus tremuloides GN=rbcL PE=3 SV=1 | 2 | 0.710 | 0.175 | 0.032 | PS |
| 150 | SP://P48717 | atCg00490 | RBL_TROMA Ribulose bisphosphate carboxylase large chain (Fragment) OS=Tropaeolum majus GN=rbcL PE=3 SV=2 | 2 | 0.710 | 0.175 | 0.032 | PS |
| 151 | SP://P92463 | atCg00490 | RBL_ISOTA Ribulose bisphosphate carboxylase large chain (Fragment) OS=Isophysis tasmanica GN=rbcL PE=3 SV=1 | 2 | 0.710 | 0.175 | 0.032 | PS |
| 152 | SP://P93890 | atCg00490 | RBL_ARIGL Ribulose bisphosphate carboxylase large chain (Fragment) OS=Aristea glauca GN=rbcL PE=3 SV=1 | 2 | 0.710 | 0.175 | 0.032 | PS |
| 153 | SP://P93936 | atCg00490 | RBL_WatAN Ribulose bisphosphate carboxylase large chain (Fragment) OS=Watsonia angusta GN=rbcL PE=3 SV=1 | 2 | 0.710 | 0.175 | 0.032 | PS |
| 154 | SP://Q06GY9 | atCg00490 | RBL_DRIGR Ribulose bisphosphate carboxylase large chain OS=Drimys granadensis GN=rbcL PE=3 SV=1 | 2 | 0.710 | 0.175 | 0.032 | PS |
| 155 | SP://Q07210 | atCg00490 | RBL_COLPU Ribulose bisphosphate carboxylase large chain (Fragment) OS=Coleonema pulchellum GN=rbcL PE=3 SV=1 | 2 | 0.710 | 0.175 | 0.032 | PS |
| 156 | SP://Q09MH0 | atCg00490 | RBL_CITSI Ribulose bisphosphate carboxylase large chain OS=Citrus sinensis GN=rbcL PE=3 SV=1 | 2 | 0.710 | 0.175 | 0.032 | PS |
| 157 | SP://Q0G9V4 | atCg00490 | RBL_DAUCA Ribulose bisphosphate carboxylase large chain OS=Daucus carota GN=rbcL PE=3 SV=1 | 2 | 0.710 | 0.175 | 0.032 | PS |
| 158 | SP://Q1ACK0 | atCg00490 | RBL_CHAVU Ribulose bisphosphate carboxylase large chain OS=Chara vulgaris GN=rbcL PE=3 SV=1 | 2 | 0.710 | 0.175 | 0.032 | PS |
| 159 | SP://Q2MIH9 | atCg00490 | RBL_SOLBU Ribulose bisphosphate carboxylase large chain OS=Solanum bulbocastanum GN=rbcL PE=3 SV=1 | 2 | 0.710 | 0.175 | 0.032 | PS |
| 160 | SP://Q31672 | atCg00490 | RBL_ANTLU Ribulose bisphosphate carboxylase large chain (Fragment) OS=Antirhea lucida GN=rbcL PE=3 SV=1 | 2 | 0.710 | 0.175 | 0.032 | PS |
| 161 | SP://Q31730 | atCg00490 | RBL_BAURU Ribulose bisphosphate carboxylase large chain (Fragment) OS=Bauera rubioides GN=rbcL PE=3 SV=2 | 2 | 0.710 | 0.175 | 0.032 | PS |
| 162 | SP://Q31738 | atCg00490 | RBL_BREMA Ribulose bisphosphate carboxylase large chain (Fragment) OS=Brexia madagascariensis GN=rbcL PE=3 SV=1 | 2 | 0.710 | 0.175 | 0.032 | PS |
| 163 | SP://Q31795 | atCg00490 | RBL_ANTFO Ribulose bisphosphate carboxylase large chain OS=Anthoceros formosae GN=rbcL PE=2 SV=2 | 2 | 0.710 | 0.175 | 0.032 | PS |
| 164 | SP://Q31827 | atCg00490 | RBL_AESPA Ribulose bisphosphate carboxylase large chain (Fragment) OS=Aesculus pavia GN=rbcL PE=3 SV=2 | 2 | 0.710 | 0.175 | 0.032 | PS |

| | | | | | | | | |
|---|---|---|---|---|---|---|---|---|
| 165 | SP://Q31859 | atCg00490 | RBL_ANTVS Ribulose bisphosphate carboxylase large chain (Fragment) OS=Anthocercis viscosa GN=rbcL PE=3 SV=1 | 2 | 0.710 | 0.175 | 0.032 | PS |
| 166 | SP://Q31948 | atCg00490 | RBL_CORat Ribulose bisphosphate carboxylase large chain (Fragment) OS=Cornus alternifolia GN=rbcL PE=3 SV=3 | 2 | 0.710 | 0.175 | 0.032 | PS |
| 167 | SP://Q31951 | atCg00490 | RBL_CAPBA Ribulose bisphosphate carboxylase large chain (Fragment) OS=Capsicum baccatum GN=rbcL PE=3 SV=1 | 2 | 0.710 | 0.175 | 0.032 | PS |
| 168 | SP://Q32026 | atCg00490 | RBL_CUNLA Ribulose bisphosphate carboxylase large chain (Fragment) OS=Cunninghamia lanceolata GN=rbcL PE=3 SV=2 | 2 | 0.710 | 0.175 | 0.032 | PS |
| 169 | SP://Q32397 | atCg00490 | RBL_HYDFO Ribulose bisphosphate carboxylase large chain (Fragment) OS=Hydnophytum formicarum GN=rbcL PE=3 SV=1 | 2 | 0.710 | 0.175 | 0.032 | PS |
| 170 | SP://Q32686 | atCg00490 | RBL_NICPH Ribulose bisphosphate carboxylase large chain (Fragment) OS=Nicandra physalodes GN=rbcL PE=3 SV=1 | 2 | 0.710 | 0.175 | 0.032 | PS |
| 171 | SP://Q32699 | atCg00490 | RBL_NOLSP Ribulose bisphosphate carboxylase large chain (Fragment) OS=Nolana spathulata GN=rbcL PE=3 SV=1 | 2 | 0.710 | 0.175 | 0.032 | PS |
| 172 | SP://Q32701 | atCg00490 | RBL_NOTSU Ribulose bisphosphate carboxylase large chain OS=Notothixos subaureus GN=rbcL PE=3 SV=1 | 2 | 0.710 | 0.175 | 0.032 | PS |
| 173 | SP://Q33101 | atCg00490 | RBL_SOLGR Ribulose bisphosphate carboxylase large chain (Fragment) OS=Solandra grandiflora GN=rbcL PE=3 SV=1 | 2 | 0.710 | 0.175 | 0.032 | PS |
| 174 | SP://Q33369 | atCg00490 | RBL_CORKO Ribulose bisphosphate carboxylase large chain (Fragment) OS=Cornus kousa GN=rbcL PE=3 SV=1 | 2 | 0.710 | 0.175 | 0.032 | PS |
| 175 | SP://Q33443 | atCg00490 | RBL_EREMA Ribulose bisphosphate carboxylase large chain (Fragment) OS=Eremothamnus marlothianus GN=rbcL PE=3 SV=1 | 2 | 0.710 | 0.175 | 0.032 | PS |
| 176 | SP://Q33584 | atCg00490 | RBL_LatCL Ribulose bisphosphate carboxylase large chain OS=Lathraea clandestina GN=rbcL PE=3 SV=1 | 2 | 0.710 | 0.175 | 0.032 | PS |
| 177 | SP://Q33C26 | atCg00490 | RBL_NICTO Ribulose bisphosphate carboxylase large chain OS=Nicotiana tomentosiformis GN=rbcL PE=3 SV=1 | 2 | 0.710 | 0.175 | 0.032 | PS |
| 178 | SP://Q36800 | atCg00490 | RBL_STRLC Ribulose bisphosphate carboxylase large chain (Fragment) OS=Streptopus lanceolatus GN=rbcL PE=3 SV=1 | 2 | 0.710 | 0.175 | 0.032 | PS |
| 179 | SP://Q3BAN4 | atCg00490 | RBL_PHAAO Ribulose bisphosphate carboxylase large chain OS=Phalaenopsis aphrodite subsp. formosana GN=rbcL PE=3 SV=2 | 2 | 0.710 | 0.175 | 0.032 | PS |
| 180 | SP://Q3C1J4 | atCg00490 | RBL_NICSY Ribulose bisphosphate carboxylase large chain OS=Nicotiana sylvestris GN=rbcL PE=3 SV=1 | 2 | 0.710 | 0.175 | 0.032 | PS |
| 181 | SP://Q49KZ0 | atCg00490 | RBL_EUCGG Ribulose bisphosphate carboxylase large chain OS=Eucalyptus globulus subsp. globulus GN=rbcL PE=3 SV=1 | 2 | 0.710 | 0.175 | 0.032 | PS |
| 182 | SP://Q68RZ8 | atCg00490 | RBL_PANGI Ribulose bisphosphate carboxylase large chain OS=Panax ginseng GN=rbcL PE=3 SV=1 | 2 | 0.710 | 0.175 | 0.032 | PS |
| 183 | SP://Q7GUD0 | atCg00490 | RBL_PINKO Ribulose bisphosphate carboxylase large chain OS=Pinus koraiensis GN=rbcL PE=3 SV=1 | 2 | 0.710 | 0.175 | 0.032 | PS |
| 184 | SP://Q8RU60 | atCg00490 | RBL_atRBE Ribulose bisphosphate carboxylase large chain OS=atropa belladonna GN=rbcL PE=3 SV=1 | 2 | 0.710 | 0.175 | 0.032 | PS |
| 185 | SP://Q95694 | atCg00490 | RBL_CAMLE Ribulose bisphosphate carboxylase large chain (Fragment) OS=Camassia leichtlinii GN=rbcL PE=3 SV=1 | 2 | 0.710 | 0.175 | 0.032 | PS |
| 186 | SP://Q9BA49 | atCg00490 | RBL_HYOLA Ribulose bisphosphate carboxylase large chain OS=Hyophorbe lagenicaulis GN=rbcL PE=3 SV=1 | 2 | 0.710 | 0.175 | 0.032 | PS |
| 187 | SP://Q9GHP8 | atCg00490 | RBL_POLSI Ribulose bisphosphate carboxylase large chain (Fragment) OS=Poliothyrsis sinensis GN=rbcL PE=3 SV=1 | 2 | 0.710 | 0.175 | 0.032 | PS |
| 188 | SP://Q9MVE5 | atCg00490 | RBL_TAXDI Ribulose bisphosphate carboxylase large chain OS=Taxodium distichum GN=rbcL PE=3 SV=1 | 2 | 0.710 | 0.175 | 0.032 | PS |
| 189 | SP://Q9XPR5 | atCg00490 | RBL_IDEPO Ribulose bisphosphate carboxylase large chain (Fragment) OS=Idesia polycarpa GN=rbcL PE=3 SV=1 | 2 | 0.710 | 0.175 | 0.032 | PS |
| 190 | SP://O98681 | atCg00490 | RBL_ZAMZA Ribulose bisphosphate carboxylase large chain (Fragment) OS=Zamioculcas zamiifolia GN=rbcL PE=3 SV=1 | 4 | 0.709 | 0.160 | 0.007 | PS |
| 191 | SP://P28383 | atCg00490 | RBL_BEGMS Ribulose bisphosphate carboxylase large chain (Fragment) OS=Begonia metallica x Begonia sanguinea | 4 | 0.709 | 0.160 | 0.007 | PS |
| 192 | SP://P48711 | atCg00490 | RBL_PICAB Ribulose bisphosphate carboxylase large chain OS=Picea abies GN=rbcL PE=2 SV=2 | 4 | 0.709 | 0.160 | 0.007 | PS |
| 193 | SP://P48719 | atCg00490 | RBL_WELMI Ribulose bisphosphate carboxylase large chain OS=Welwitschia mirabilis GN=rbcL PE=3 SV=2 | 4 | 0.709 | 0.160 | 0.007 | PS |
| 194 | SP://P93913 | atCg00490 | RBL_LIRMU Ribulose bisphosphate carboxylase large chain (Fragment) OS=Liriope muscari GN=rbcL PE=3 SV=1 | 4 | 0.709 | 0.160 | 0.007 | PS |
| 195 | SP://Q01874 | atCg00490 | RBL_QUERU Ribulose bisphosphate carboxylase large chain OS=Quercus rubra GN=rbcL PE=3 SV=1 | 4 | 0.709 | 0.160 | 0.007 | PS |
| 196 | SP://Q39024 | at4g01370 | MPK4_ARatH Mitogen_activated protein kinase 4 OS=Arabidopsis thaliana GN=MPK4 PE=1 SV=2 | 2 | 0.706 | 0.117 | 0.001 | signalling |
| 197 | SP://Q9STW6 | at4g24280 | HSP7F_ARatH Heat shock 70 kDa protein 6_ chloroplastic OS=Arabidopsis thaliana GN=HSP70_6 PE=1 SV=1 | 6 | 0.705 | 0.118 | 0.001 | stress |
| 198 | SP://Q9S9N1 | at1g16030 | HSP7E_ARatH Heat shock 70 kDa protein 5 OS=Arabidopsis thaliana GN=HSP70_5 PE=2 SV=1 | 7 | 0.704 | 0.141 | 0.036 | stress |

| | | | | | | | | |
|---|---|---|---|---|---|---|---|---|
| 199 | SP://Q69QQ6 | at5g56000 | HSP82_ORYSJ Heat shock protein 81_2 OS=Oryza sativa subsp. japonica GN=HSP81_2 PE=2 SV=1 | 13 | 0.703 | 0.112 | 0.009 | stress |
| 200 | SP://B3TN59 | atCg00490 | RBL_BRADI Ribulose bisphosphate carboxylase large chain OS=Brachypodium distachyon GN=rbcL PE=3 SV=1 | 5 | 0.703 | 0.153 | 0.007 | PS |
| 201 | SP://P00874 | atCg00490 | RBL_MAIZE Ribulose bisphosphate carboxylase large chain OS=Zea mays GN=rbcL PE=3 SV=2 | 5 | 0.703 | 0.153 | 0.007 | PS |
| 202 | SP://P19163 | atCg00490 | RBL_NEUMU Ribulose bisphosphate carboxylase large chain OS=Neurachne munroi GN=rbcL PE=3 SV=1 | 5 | 0.703 | 0.153 | 0.007 | PS |
| 203 | SP://P19164 | atCg00490 | RBL_NEUTE Ribulose bisphosphate carboxylase large chain OS=Neurachne tenuifolia GN=rbcL PE=3 SV=1 | 5 | 0.703 | 0.153 | 0.007 | PS |
| 204 | SP://P56647 | atCg00490 | RBL_SETIT Ribulose bisphosphate carboxylase large chain OS=Setaria italica GN=rbcL PE=3 SV=1 | 5 | 0.703 | 0.153 | 0.007 | PS |
| 205 | SP://Q6L391 | atCg00490 | RBL_SACHY Ribulose bisphosphate carboxylase large chain OS=Saccharum hybrid GN=rbcL PE=3 SV=1 | 5 | 0.703 | 0.153 | 0.007 | PS |
| 206 | SP://A4QJC3 | atCg00490 | RBL_AETCO Ribulose bisphosphate carboxylase large chain OS=Aethionema cordifolium GN=rbcL PE=3 SV=1 | 3 | 0.702 | 0.166 | 0.036 | PS |
| 207 | SP://A4QJK7 | atCg00490 | RBL_AETGR Ribulose bisphosphate carboxylase large chain OS=Aethionema grandiflorum GN=rbcL PE=3 SV=1 | 3 | 0.702 | 0.166 | 0.036 | PS |
| 208 | SP://A4QKB3 | atCg00490 | RBL_BARVE Ribulose bisphosphate carboxylase large chain OS=Barbarea verna GN=rbcL PE=3 SV=1 | 3 | 0.702 | 0.166 | 0.036 | PS |
| 209 | SP://A4QKK0 | atCg00490 | RBL_CAPBU Ribulose bisphosphate carboxylase large chain OS=Capsella bursa_pastoris GN=rbcL PE=3 SV=1 | 3 | 0.702 | 0.166 | 0.036 | PS |
| 210 | SP://A4QKT9 | atCg00490 | RBL_CRUWA Ribulose bisphosphate carboxylase large chain OS=Crucihimalaya wallichii GN=rbcL PE=3 SV=1 | 3 | 0.702 | 0.166 | 0.036 | PS |
| 211 | SP://A4QL27 | atCg00490 | RBL_DRANE Ribulose bisphosphate carboxylase large chain OS=Draba nemorosa GN=rbcL PE=3 SV=1 | 3 | 0.702 | 0.166 | 0.036 | PS |
| 212 | SP://A4QLB4 | atCg00490 | RBL_LEPVR Ribulose bisphosphate carboxylase large chain OS=Lepidium virginicum GN=rbcL PE=3 SV=1 | 3 | 0.702 | 0.166 | 0.036 | PS |
| 213 | SP://A4QLK2 | atCg00490 | RBL_LOBMA Ribulose bisphosphate carboxylase large chain OS=Lobularia maritima GN=rbcL PE=3 SV=1 | 3 | 0.702 | 0.166 | 0.036 | PS |
| 214 | SP://B0Z4K9 | atCg00490 | RBL_OENAR Ribulose bisphosphate carboxylase large chain OS=Oenothera argillicola GN=rbcL PE=3 SV=1 | 3 | 0.702 | 0.166 | 0.036 | PS |
| 215 | SP://O03042 | atCg00490 | RBL_ARatH Ribulose bisphosphate carboxylase large chain OS=Arabidopsis thaliana GN=rbcL PE=1 SV=1 | 3 | 0.702 | 0.166 | 0.036 | PS |
| 216 | SP://O98664 | atCg00490 | RBL_KIGAF Ribulose bisphosphate carboxylase large chain (Fragment) OS=Kigelia africana GN=rbcL PE=3 SV=1 | 3 | 0.702 | 0.166 | 0.036 | PS |
| 217 | SP://O98668 | atCg00490 | RBL_PANJS Ribulose bisphosphate carboxylase large chain (Fragment) OS=Pandorea jasminoides GN=rbcL PE=3 SV=1 | 3 | 0.702 | 0.166 | 0.036 | PS |
| 218 | SP://O98671 | atCg00490 | RBL_TECST Ribulose bisphosphate carboxylase large chain (Fragment) OS=Tecoma stans GN=rbcL PE=3 SV=1 | 3 | 0.702 | 0.166 | 0.036 | PS |
| 219 | SP://O98890 | atCg00490 | RBL_HALCL Ribulose bisphosphate carboxylase large chain (Fragment) OS=Halesia carolina GN=rbcL PE=3 SV=1 | 3 | 0.702 | 0.166 | 0.036 | PS |
| 220 | SP://O99000 | atCg00490 | RBL_ADEOB Ribulose bisphosphate carboxylase large chain (Fragment) OS=Adenium obesum GN=rbcL PE=3 SV=1 | 3 | 0.702 | 0.166 | 0.036 | PS |
| 221 | SP://P28379 | atCg00490 | RBL_APHSI Ribulose bisphosphate carboxylase large chain (Fragment) OS=Aphelandra sinclairiana GN=rbcL PE=3 SV=1 | 3 | 0.702 | 0.166 | 0.036 | PS |
| 222 | SP://P28382 | atCg00490 | RBL_BARPR Ribulose bisphosphate carboxylase large chain (Fragment) OS=Barleria prionitis GN=rbcL PE=3 SV=2 | 3 | 0.702 | 0.166 | 0.036 | PS |
| 223 | SP://P28386 | atCg00490 | RBL_BYBLI Ribulose bisphosphate carboxylase large chain (Fragment) OS=Byblis liniflora GN=rbcL PE=3 SV=3 | 3 | 0.702 | 0.166 | 0.036 | PS |
| 224 | SP://P28392 | atCg00490 | RBL_CLAXA Ribulose bisphosphate carboxylase large chain OS=Clarkia xantiana GN=rbcL PE=2 SV=1 | 3 | 0.702 | 0.166 | 0.036 | PS |
| 225 | SP://P28395 | atCg00490 | RBL_CRAMA Ribulose bisphosphate carboxylase large chain (Fragment) OS=Crassula marnierana GN=rbcL PE=3 SV=1 | 3 | 0.702 | 0.166 | 0.036 | PS |
| 226 | SP://P28413 | atCg00490 | RBL_ERICA Ribulose bisphosphate carboxylase large chain (Fragment) OS=Eriodictyon californicum GN=rbcL PE=3 SV=1 | 3 | 0.702 | 0.166 | 0.036 | PS |
| 227 | SP://P28420 | atCg00490 | RBL_UNCGR Ribulose bisphosphate carboxylase large chain (Fragment) OS=Uncarina grandidieri GN=rbcL PE=3 SV=1 | 3 | 0.702 | 0.166 | 0.036 | PS |
| 228 | SP://P28425 | atCg00490 | RBL_HYDVI Ribulose bisphosphate carboxylase large chain (Fragment) OS=Hydrophyllum virginianum GN=rbcL PE=3 SV=1 | 3 | 0.702 | 0.166 | 0.036 | PS |
| 229 | SP://P28432 | atCg00490 | RBL_MORCE Ribulose bisphosphate carboxylase large chain (Fragment) OS=Morella cerifera GN=rbcL PE=3 SV=1 | 3 | 0.702 | 0.166 | 0.036 | PS |
| 230 | SP://P28433 | atCg00490 | RBL_NELCA Ribulose bisphosphate carboxylase large chain (Fragment) OS=Nelsonia canescens GN=rbcL PE=3 SV=1 | 3 | 0.702 | 0.166 | 0.036 | PS |
| 231 | SP://P28440 | atCg00490 | RBL_PINCA Ribulose bisphosphate carboxylase large chain (Fragment) OS=Pinguicula caerulea GN=rbcL PE=3 SV=1 | 3 | 0.702 | 0.166 | 0.036 | PS |
| 232 | SP://P28444 | atCg00490 | RBL_PROLU Ribulose bisphosphate carboxylase large chain (Fragment) OS=Proboscidea lousianica GN=rbcL PE=3 SV=1 | 3 | 0.702 | 0.166 | 0.036 | PS |

| | | | | | | | | |
|---|---|---|---|---|---|---|---|---|
| 233 | SP://P30732 | atCg00490 | RBL_MAGAC Ribulose bisphosphate carboxylase large chain OS=Magnolia acuminata GN=rbcL PE=3 SV=1 | 3 | 0.702 | 0.166 | 0.036 | PS |
| 234 | SP://P30827 | atCg00490 | RBL_LIRTU Ribulose bisphosphate carboxylase large chain OS=Liriodendron tulipifera GN=rbcL PE=3 SV=1 | 3 | 0.702 | 0.166 | 0.036 | PS |
| 235 | SP://P30829 | atCg00490 | RBL_MAGMA Ribulose bisphosphate carboxylase large chain OS=Magnolia macrophylla GN=rbcL PE=3 SV=1 | 3 | 0.702 | 0.166 | 0.036 | PS |
| 236 | SP://P31183 | atCg00490 | RBL_CORLA Ribulose bisphosphate carboxylase large chain (Fragment) OS=Corynocarpus laevigatus GN=rbcL PE=3 SV=2 | 3 | 0.702 | 0.166 | 0.036 | PS |
| 237 | SP://P36478 | atCg00490 | RBL_ANTGA Ribulose bisphosphate carboxylase large chain (Fragment) OS=Anthocleista grandiflora GN=rbcL PE=3 SV=1 | 3 | 0.702 | 0.166 | 0.036 | PS |
| 238 | SP://P36481 | atCg00490 | RBL_BERLA Ribulose bisphosphate carboxylase large chain (Fragment) OS=Berzelia lanuginosa GN=rbcL PE=3 SV=1 | 3 | 0.702 | 0.166 | 0.036 | PS |
| 239 | SP://P36485 | atCg00490 | RBL_SALDI Ribulose bisphosphate carboxylase large chain (Fragment) OS=Salvia divinorum GN=rbcL PE=3 SV=2 | 3 | 0.702 | 0.166 | 0.036 | PS |
| 240 | SP://P36488 | atCg00490 | RBL_SPIMR Ribulose bisphosphate carboxylase large chain (Fragment) OS=Spigelia marilandica GN=rbcL PE=3 SV=2 | 3 | 0.702 | 0.166 | 0.036 | PS |
| 241 | SP://P48694 | atCg00490 | RBL_COFAR Ribulose bisphosphate carboxylase large chain OS=Coffea arabica GN=rbcL PE=3 SV=2 | 3 | 0.702 | 0.166 | 0.036 | PS |
| 242 | SP://P48695 | atCg00490 | RBL_COLOB Ribulose bisphosphate carboxylase large chain OS=Coleochaete orbicularis GN=rbcL PE=3 SV=1 | 3 | 0.702 | 0.166 | 0.036 | PS |
| 243 | SP://P48697 | atCg00490 | RBL_CUCPE Ribulose bisphosphate carboxylase large chain (Fragment) OS=Cucurbita pepo GN=rbcL PE=3 SV=1 | 3 | 0.702 | 0.166 | 0.036 | PS |
| 244 | SP://Q06GQ2 | atCg00490 | RBL_PIPCE Ribulose bisphosphate carboxylase large chain OS=Piper cenocladum GN=rbcL PE=3 SV=1 | 3 | 0.702 | 0.166 | 0.036 | PS |
| 245 | SP://Q06RC1 | atCg00490 | RBL_JASNU Ribulose bisphosphate carboxylase large chain OS=Jasminum nudiflorum GN=rbcL PE=3 SV=1 | 3 | 0.702 | 0.166 | 0.036 | PS |
| 246 | SP://Q31715 | atCg00490 | RBL_BERBR Ribulose bisphosphate carboxylase large chain (Fragment) OS=Bertiera breviflora GN=rbcL PE=3 SV=1 | 3 | 0.702 | 0.166 | 0.036 | PS |
| 247 | SP://Q31809 | atCg00490 | RBL_ASPLA Ribulose bisphosphate carboxylase large chain (Fragment) OS=Asperula laevigata GN=rbcL PE=3 SV=1 | 3 | 0.702 | 0.166 | 0.036 | PS |
| 248 | SP://Q31946 | atCg00490 | RBL_CRUAN Ribulose bisphosphate carboxylase large chain (Fragment) OS=Crucianella angustifolia GN=rbcL PE=3 SV=1 | 3 | 0.702 | 0.166 | 0.036 | PS |
| 249 | SP://Q31992 | atCg00490 | RBL_CRUGL Ribulose bisphosphate carboxylase large chain (Fragment) OS=Cruciata glabra GN=rbcL PE=3 SV=1 | 3 | 0.702 | 0.166 | 0.036 | PS |
| 250 | SP://Q32255 | atCg00490 | RBL_GALAL Ribulose bisphosphate carboxylase large chain (Fragment) OS=Galium album GN=rbcL PE=3 SV=1 | 3 | 0.702 | 0.166 | 0.036 | PS |
| 251 | SP://Q32256 | atCg00490 | RBL_GALAP Ribulose bisphosphate carboxylase large chain (Fragment) OS=Galium aparine GN=rbcL PE=3 SV=1 | 3 | 0.702 | 0.166 | 0.036 | PS |
| 252 | SP://Q32271 | atCg00490 | RBL_GALCO Ribulose bisphosphate carboxylase large chain (Fragment) OS=Galium corsicum GN=rbcL PE=3 SV=1 | 3 | 0.702 | 0.166 | 0.036 | PS |
| 253 | SP://Q32283 | atCg00490 | RBL_GALEL Ribulose bisphosphate carboxylase large chain (Fragment) OS=Galium elongatum GN=rbcL PE=3 SV=1 | 3 | 0.702 | 0.166 | 0.036 | PS |
| 254 | SP://Q32303 | atCg00490 | RBL_GALLU Ribulose bisphosphate carboxylase large chain (Fragment) OS=Galium lucidum GN=rbcL PE=3 SV=1 | 3 | 0.702 | 0.166 | 0.036 | PS |
| 255 | SP://Q32344 | atCg00490 | RBL_GALPR Ribulose bisphosphate carboxylase large chain (Fragment) OS=Galium parisiense GN=rbcL PE=3 SV=1 | 3 | 0.702 | 0.166 | 0.036 | PS |
| 256 | SP://Q32345 | atCg00490 | RBL_GALPA Ribulose bisphosphate carboxylase large chain (Fragment) OS=Galium palustre GN=rbcL PE=3 SV=1 | 3 | 0.702 | 0.166 | 0.036 | PS |
| 257 | SP://Q32360 | atCg00490 | RBL_GALSC Ribulose bisphosphate carboxylase large chain (Fragment) OS=Galium scabrum GN=rbcL PE=3 SV=1 | 3 | 0.702 | 0.166 | 0.036 | PS |
| 258 | SP://Q32908 | atCg00490 | RBL_PHUST Ribulose bisphosphate carboxylase large chain (Fragment) OS=Phuopsis stylosa GN=rbcL PE=3 SV=1 | 3 | 0.702 | 0.166 | 0.036 | PS |
| 259 | SP://Q33050 | atCg00490 | RBL_RUBTI Ribulose bisphosphate carboxylase large chain (Fragment) OS=Rubia tinctorum GN=rbcL PE=3 SV=1 | 3 | 0.702 | 0.166 | 0.036 | PS |
| 260 | SP://Q33062 | atCg00490 | RBL_SHEAR Ribulose bisphosphate carboxylase large chain (Fragment) OS=Sherardia arvensis GN=rbcL PE=3 SV=1 | 3 | 0.702 | 0.166 | 0.036 | PS |
| 261 | SP://Q33274 | atCg00490 | RBL_VALMU Ribulose bisphosphate carboxylase large chain (Fragment) OS=Valantia muralis GN=rbcL PE=3 SV=1 | 3 | 0.702 | 0.166 | 0.036 | PS |
| 262 | SP://Q37282 | atCg00490 | RBL_TABHE Ribulose bisphosphate carboxylase large chain (Fragment) OS=Tabebuia heterophylla GN=rbcL PE=3 SV=1 | 3 | 0.702 | 0.166 | 0.036 | PS |
| 263 | SP://Q3V526 | atCg00490 | RBL_ACOCL Ribulose bisphosphate carboxylase large chain OS=Acorus calamus GN=rbcL PE=3 SV=2 | 3 | 0.702 | 0.166 | 0.036 | PS |
| 264 | SP://Q8SN66 | atCg00490 | RBL_CHAGL Ribulose bisphosphate carboxylase large chain OS=Chaetosphaeridium globosum GN=rbcL PE=3 SV=2 | 3 | 0.702 | 0.166 | 0.036 | PS |
| 265 | SP://Q07078 | at5g56000 | HSP83_ORYSJ Heat shock protein 81_3 OS=Oryza sativa subsp. japonica GN=HSP81_3 PE=2 SV=2 | 15 | 0.701 | 0.111 | 0.020 | stress |
| 266 | SP://Q02028 | at5g49910 | HSP7S_PEA Stromal 70 kDa heat shock_related protein_ chloroplastic OS=Pisum sativum GN=HSP70 PE=2 SV=1 | 3 | 0.698 | 0.119 | 0.001 | stress |

| | | | | | | | | |
|---|---|---|---|---|---|---|---|---|
| 267 | SP://P31922 | at3g43190 | SUS1_HORVU Sucrose synthase 1 OS=Hordeum vulgare GN=SS1 PE=1 SV=1 | 31 | 0.695 | 0.056 | 0.000 | major CHO metabolism |
| 268 | SP://A2YWQ1 | at5g56000 | HSP81_ORYSI Heat shock protein 81_1 OS=Oryza sativa subsp. indica GN=HSP81_1 PE=2 SV=1 | 15 | 0.694 | 0.108 | 0.009 | stress |
| 269 | SP://P28430 | atCg00490 | RBL_MANZA Ribulose bisphosphate carboxylase large chain (Fragment) OS=Manilkara zapota GN=rbcL PE=3 SV=2 | 5 | 0.692 | 0.156 | 0.007 | PS |
| 270 | SP://P36484 | atCg00490 | RBL_CHIAL Ribulose bisphosphate carboxylase large chain (Fragment) OS=Chiococca alba GN=rbcL PE=3 SV=1 | 3 | 0.691 | 0.190 | 0.034 | PS |
| 271 | SP://P48686 | atCg00490 | RBL_BRAOL Ribulose bisphosphate carboxylase large chain OS=Brassica oleracea GN=rbcL PE=3 SV=1 | 3 | 0.690 | 0.170 | 0.035 | PS |
| 272 | SP://Q33383 | atCg00490 | RBL_CatSP Ribulose bisphosphate carboxylase large chain (Fragment) OS=Catesbaea spinosa GN=rbcL PE=3 SV=1 | 3 | 0.690 | 0.170 | 0.035 | PS |
| 273 | SP://Q9LY32 | at3g60330 | PMA7_ARatH atPase 7_ plasma membrane_type OS=Arabidopsis thaliana GN=AHA7 PE=2 SV=1 | 3 | 0.688 | 0.140 | 0.001 | transport |
| 274 | SP://Q336X9 | at4g01370 | MPK6_ORYSJ Mitogen_activated protein kinase 6 OS=Oryza sativa subsp. japonica GN=MPK6 PE=2 SV=1 | 3 | 0.687 | 0.109 | 0.001 | signalling |
| 275 | SP://A4QJU1 | atCg00490 | RBL_OLIPU Ribulose bisphosphate carboxylase large chain OS=Olimarabidopsis pumila GN=rbcL PE=3 SV=1 | 4 | 0.685 | 0.162 | 0.035 | PS |
| 276 | SP://A4QK26 | atCg00490 | RBL_ARAHI Ribulose bisphosphate carboxylase large chain OS=Arabis hirsuta GN=rbcL PE=3 SV=1 | 4 | 0.685 | 0.162 | 0.035 | PS |
| 277 | SP://A4QLU1 | atCg00490 | RBL_NASOF Ribulose bisphosphate carboxylase large chain OS=Nasturtium officinale GN=rbcL PE=3 SV=1 | 4 | 0.685 | 0.162 | 0.035 | PS |
| 278 | SP://Q33406 | atCg00490 | RBL_DEPGR Ribulose bisphosphate carboxylase large chain (Fragment) OS=Deppea grandiflora GN=rbcL PE=3 SV=1 | 4 | 0.685 | 0.162 | 0.035 | PS |
| 279 | SP://O98883 | atCg00490 | RBL_COUGU Ribulose bisphosphate carboxylase large chain (Fragment) OS=Couroupita guianensis GN=rbcL PE=3 SV=1 | 3 | 0.683 | 0.245 | 0.035 | PS |
| 280 | SP://Q37328 | atCg00490 | RBL_EPHSI Ribulose bisphosphate carboxylase large chain (Fragment) OS=Ephedra sinica GN=rbcL PE=3 SV=1 | 3 | 0.683 | 0.245 | 0.035 | PS |
| 281 | SP://Q08435 | at5g62670 | PMA1_NICPL Plasma membrane atPase 1 OS=Nicotiana plumbaginifolia GN=PMA1 PE=2 SV=1 | 4 | 0.682 | 0.130 | 0.001 | transport |
| 282 | SP://P08823 | at2g28000 | RUBA_WHEat RuBisCO large subunit_binding protein subunit alpha_ chloroplastic (Fragment) OS=Triticum aestivum | 3 | 0.682 | 0.166 | 0.017 | PS |
| 283 | SP://P48495 | at3g55440 | TPIS_PETHY Triosephosphate isomerase_ cytosolic OS=Petunia hybrida GN=TPIP1 PE=2 SV=1 | 3 | 0.682 | 0.176 | 0.005 | glycolysis |
| 284 | SP://P23980 | at5g62670 | PMA2_SOLLC Plasma membrane atPase 2 (Fragment) OS=Solanum lycopersicum GN=LHA2 PE=3 SV=1 | 5 | 0.680 | 0.130 | 0.001 | transport |
| 285 | SP://P22180 | at5g62670 | PMA1_SOLLC Plasma membrane atPase 1 OS=Solanum lycopersicum GN=LHA1 PE=2 SV=1 | 6 | 0.680 | 0.125 | 0.001 | transport |
| 286 | SP://Q08436 | at5g62670 | PMA3_NICPL Plasma membrane atPase 3 OS=Nicotiana plumbaginifolia GN=PMA3 PE=1 SV=1 | 6 | 0.680 | 0.125 | 0.001 | transport |
| 287 | SP://Q9SPB1 | at1g48030 | LEGRE_VIGUN Leghemoglobin reductase OS=Vigna unguiculata GN=FLBR PE=1 SV=1 | 4 | 0.679 | 0.182 | 0.016 | TCA |
| 288 | SP://P31252 | at5g06460 | UBE13_WHEat Ubiquitin_activating enzyme E1 3 OS=Triticum aestivum GN=UBA3 PE=2 SV=1 | 2 | 0.679 | 0.147 | 0.034 | protein |
| 289 | SP://P28381 | atCg00490 | RBL_BARCA Ribulose bisphosphate carboxylase large chain OS=Barnadesia caryophylla GN=rbcL PE=3 SV=1 | 2 | 0.679 | 0.197 | 0.001 | PS |
| 290 | SP://P51101 | atCg00490 | RBL_GERJA Ribulose bisphosphate carboxylase large chain OS=Gerbera jamesonii GN=rbcL PE=3 SV=1 | 2 | 0.679 | 0.197 | 0.001 | PS |
| 291 | SP://Q05987 | atCg00490 | RBL_CERJA Ribulose bisphosphate carboxylase large chain (Fragment) OS=Cercidiphyllum japonicum GN=rbcL PE=3 SV=1 | 2 | 0.679 | 0.197 | 0.001 | PS |
| 292 | SP://Q7YJW4 | atCg00490 | RBL_CALFG Ribulose bisphosphate carboxylase large chain OS=Calycanthus floridus var. glaucus GN=rbcL PE=3 SV=1 | 2 | 0.679 | 0.197 | 0.001 | PS |
| 293 | SP://A4GG89 | atCg00490 | RBL_PHAVU Ribulose bisphosphate carboxylase large chain OS=Phaseolus vulgaris GN=rbcL PE=3 SV=1 | 3 | 0.676 | 0.167 | 0.002 | PS |
| 294 | SP://A6BM42 | atCg00490 | RBL_GNEPA Ribulose bisphosphate carboxylase large chain OS=Gnetum parvifolium GN=rbcL PE=3 SV=1 | 3 | 0.676 | 0.167 | 0.002 | PS |
| 295 | SP://O20241 | atCg00490 | RBL_NANDO Ribulose bisphosphate carboxylase large chain OS=Nandina domestica GN=rbcL PE=3 SV=3 | 3 | 0.676 | 0.167 | 0.002 | PS |
| 296 | SP://O20304 | atCg00490 | RBL_CASFS Ribulose bisphosphate carboxylase large chain (Fragment) OS=Cassia fistula GN=rbcL PE=3 SV=1 | 3 | 0.676 | 0.167 | 0.002 | PS |
| 297 | SP://O62943 | atCg00490 | RBL_SESSE Ribulose bisphosphate carboxylase large chain OS=Sesbania sesban GN=rbcL PE=3 SV=1 | 3 | 0.676 | 0.167 | 0.002 | PS |
| 298 | SP://O63094 | atCg00490 | RBL_CAJCA Ribulose bisphosphate carboxylase large chain OS=Cajanus cajan GN=rbcL PE=3 SV=1 | 3 | 0.676 | 0.167 | 0.002 | PS |
| 299 | SP://P04717 | atCg00490 | RBL_PEA Ribulose bisphosphate carboxylase large chain OS=Pisum sativum GN=rbcL PE=1 SV=3 | 3 | 0.676 | 0.167 | 0.002 | PS |
| 300 | SP://P28260 | atCg00490 | RBL_IPOPU Ribulose bisphosphate carboxylase large chain OS=Ipomoea purpurea GN=rbcL PE=3 SV=1 | 3 | 0.676 | 0.167 | 0.002 | PS |

| | | | | | | | | |
|---|---|---|---|---|---|---|---|---|
| 301 | SP://P28378 | atCg00490 | RBL_ADOMO Ribulose bisphosphate carboxylase large chain (Fragment) OS=Adoxa moschatellina GN=rbcL PE=3 SV=1 | 3 | 0.676 | 0.167 | 0.002 | PS |
| 302 | SP://P28384 | atCg00490 | RBL_BETNI Ribulose bisphosphate carboxylase large chain (Fragment) OS=Betula nigra GN=rbcL PE=3 SV=1 | 3 | 0.676 | 0.167 | 0.002 | PS |
| 303 | SP://P28387 | atCg00490 | RBL_BYRCR Ribulose bisphosphate carboxylase large chain (Fragment) OS=Byrsonima crassifolia GN=rbcL PE=3 SV=1 | 3 | 0.676 | 0.167 | 0.002 | PS |
| 304 | SP://P28390 | atCg00490 | RBL_CEPFO Ribulose bisphosphate carboxylase large chain (Fragment) OS=Cephalotus follicularis GN=rbcL PE=3 SV=2 | 3 | 0.676 | 0.167 | 0.002 | PS |
| 305 | SP://P28412 | atCg00490 | RBL_EPASP Ribulose bisphosphate carboxylase large chain (Fragment) OS=Epacris sp. GN=rbcL PE=3 SV=1 | 3 | 0.676 | 0.167 | 0.002 | PS |
| 306 | SP://P28414 | atCg00490 | RBL_EUCLU Ribulose bisphosphate carboxylase large chain (Fragment) OS=Eucryphia lucida GN=rbcL PE=3 SV=2 | 3 | 0.676 | 0.167 | 0.002 | PS |
| 307 | SP://P28416 | atCg00490 | RBL_GAREL Ribulose bisphosphate carboxylase large chain (Fragment) OS=Garrya elliptica GN=rbcL PE=3 SV=1 | 3 | 0.676 | 0.167 | 0.002 | PS |
| 308 | SP://P28418 | atCg00490 | RBL_GEUQU Ribulose bisphosphate carboxylase large chain (Fragment) OS=Geum quellyon GN=rbcL PE=3 SV=1 | 3 | 0.676 | 0.167 | 0.002 | PS |
| 309 | SP://P28423 | atCg00490 | RBL_HEUMI Ribulose bisphosphate carboxylase large chain (Fragment) OS=Heuchera micrantha GN=rbcL PE=3 SV=1 | 3 | 0.676 | 0.167 | 0.002 | PS |
| 310 | SP://P28426 | atCg00490 | RBL_ILECR Ribulose bisphosphate carboxylase large chain (Fragment) OS=Ilex crenata GN=rbcL PE=3 SV=1 | 3 | 0.676 | 0.167 | 0.002 | PS |
| 311 | SP://P28431 | atCg00490 | RBL_MORAL Ribulose bisphosphate carboxylase large chain (Fragment) OS=Morus alba GN=rbcL PE=3 SV=1 | 3 | 0.676 | 0.167 | 0.002 | PS |
| 312 | SP://P28435 | atCg00490 | RBL_NYSOG Ribulose bisphosphate carboxylase large chain (Fragment) OS=Nyssa ogeche GN=rbcL PE=3 SV=2 | 3 | 0.676 | 0.167 | 0.002 | PS |
| 313 | SP://P28441 | atCg00490 | RBL_PLAOC Ribulose bisphosphate carboxylase large chain OS=Platanus occidentalis GN=rbcL PE=3 SV=2 | 3 | 0.676 | 0.167 | 0.002 | PS |
| 314 | SP://P28442 | atCg00490 | RBL_PLAVR Ribulose bisphosphate carboxylase large chain (Fragment) OS=Platytheca verticillata GN=rbcL PE=3 SV=2 | 3 | 0.676 | 0.167 | 0.002 | PS |
| 315 | SP://P28446 | atCg00490 | RBL_COMID Ribulose bisphosphate carboxylase large chain (Fragment) OS=Combretum indicum GN=rbcL PE=3 SV=1 | 3 | 0.676 | 0.167 | 0.002 | PS |
| 316 | SP://P28450 | atCg00490 | RBL_SANCA Ribulose bisphosphate carboxylase large chain (Fragment) OS=Sanguinaria canadensis GN=rbcL PE=3 SV=1 | 3 | 0.676 | 0.167 | 0.002 | PS |
| 317 | SP://P28452 | atCg00490 | RBL_MICIN Ribulose bisphosphate carboxylase large chain (Fragment) OS=Micranthes integrifolia GN=rbcL PE=3 SV=1 | 3 | 0.676 | 0.167 | 0.002 | PS |
| 318 | SP://P31184 | atCg00490 | RBL_DICCR Ribulose bisphosphate carboxylase large chain (Fragment) OS=Dichapetalum crassifolium GN=rbcL PE=3 SV=2 | 3 | 0.676 | 0.167 | 0.002 | PS |
| 319 | SP://P36486 | atCg00490 | RBL_SAUCE Ribulose bisphosphate carboxylase large chain (Fragment) OS=Saururus cernuus GN=rbcL PE=3 SV=1 | 3 | 0.676 | 0.167 | 0.002 | PS |
| 320 | SP://P48689 | atCg00490 | RBL_CARTI Ribulose bisphosphate carboxylase large chain OS=Carthamus tinctorius GN=rbcL PE=3 SV=1 | 3 | 0.676 | 0.167 | 0.002 | PS |
| 321 | SP://P48690 | atCg00490 | RBL_CASSA Ribulose bisphosphate carboxylase large chain OS=Castanea sativa GN=rbcL PE=3 SV=1 | 3 | 0.676 | 0.167 | 0.002 | PS |
| 322 | SP://P48703 | atCg00490 | RBL_FRAAN Ribulose bisphosphate carboxylase large chain (Fragment) OS=Fragaria ananassa GN=rbcL PE=3 SV=1 | 3 | 0.676 | 0.167 | 0.002 | PS |
| 323 | SP://P48708 | atCg00490 | RBL_MOROL Ribulose bisphosphate carboxylase large chain (Fragment) OS=Moringa oleifera GN=rbcL PE=3 SV=1 | 3 | 0.676 | 0.167 | 0.002 | PS |
| 324 | SP://P48715 | atCg00490 | RBL_SINAL Ribulose bisphosphate carboxylase large chain (Fragment) OS=Sinapis alba GN=rbcL PE=3 SV=1 | 3 | 0.676 | 0.167 | 0.002 | PS |
| 325 | SP://P93869 | atCg00490 | RBL_TAMIN Ribulose bisphosphate carboxylase large chain (Fragment) OS=Tamarindus indica GN=rbcL PE=3 SV=1 | 3 | 0.676 | 0.167 | 0.002 | PS |
| 326 | SP://P93880 | atCg00490 | RBL_SENDI Ribulose bisphosphate carboxylase large chain (Fragment) OS=Senna didymobotrya GN=rbcL PE=3 SV=1 | 3 | 0.676 | 0.167 | 0.002 | PS |
| 327 | SP://P93988 | atCg00490 | RBL_BROCO Ribulose bisphosphate carboxylase large chain (Fragment) OS=Brownea coccinea GN=rbcL PE=3 SV=1 | 3 | 0.676 | 0.167 | 0.002 | PS |
| 328 | SP://P93998 | atCg00490 | RBL_VACFA Ribulose bisphosphate carboxylase large chain (Fragment) OS=Vachellia farnesiana GN=rbcL PE=3 SV=1 | 3 | 0.676 | 0.167 | 0.002 | PS |
| 329 | SP://Q06021 | atCg00490 | RBL_ALNIN Ribulose bisphosphate carboxylase large chain OS=Alnus incana GN=rbcL PE=3 SV=1 | 3 | 0.676 | 0.167 | 0.002 | PS |
| 330 | SP://Q06022 | atCg00490 | RBL_BETPA Ribulose bisphosphate carboxylase large chain OS=Betula papyrifera GN=rbcL PE=3 SV=1 | 3 | 0.676 | 0.167 | 0.002 | PS |
| 331 | SP://Q06023 | atCg00490 | RBL_CARCO Ribulose bisphosphate carboxylase large chain OS=Carpinus caroliniana GN=rbcL PE=3 SV=1 | 3 | 0.676 | 0.167 | 0.002 | PS |
| 332 | SP://Q06024 | atCg00490 | RBL_CORCO Ribulose bisphosphate carboxylase large chain OS=Corylus cornuta GN=rbcL PE=3 SV=1 | 3 | 0.676 | 0.167 | 0.002 | PS |
| 333 | SP://Q06025 | atCg00490 | RBL_OSTVI Ribulose bisphosphate carboxylase large chain OS=Ostrya virginiana GN=rbcL PE=3 SV=1 | 3 | 0.676 | 0.167 | 0.002 | PS |
| 334 | SP://Q09X09 | atCg00490 | RBL_MORIN Ribulose bisphosphate carboxylase large chain OS=Morus indica GN=rbcL PE=3 SV=1 | 3 | 0.676 | 0.167 | 0.002 | PS |

| | | | | | | | | |
|---|---|---|---|---|---|---|---|---|
| 335 | SP://Q31983 | atCg00490 | RBL_CORFO Ribulose bisphosphate carboxylase large chain (Fragment) OS=Cornus florida GN=rbcL PE=3 SV=2 | 3 | 0.676 | 0.167 | 0.002 | PS |
| 336 | SP://Q32040 | atCg00490 | RBL_COROB Ribulose bisphosphate carboxylase large chain (Fragment) OS=Cornus obliqua GN=rbcL PE=3 SV=2 | 3 | 0.676 | 0.167 | 0.002 | PS |
| 337 | SP://Q32041 | atCg00490 | RBL_COROL Ribulose bisphosphate carboxylase large chain (Fragment) OS=Cornus oblonga GN=rbcL PE=3 SV=2 | 3 | 0.676 | 0.167 | 0.002 | PS |
| 338 | SP://Q32042 | atCg00490 | RBL_COROF Ribulose bisphosphate carboxylase large chain (Fragment) OS=Cornus officinalis GN=rbcL PE=3 SV=2 | 3 | 0.676 | 0.167 | 0.002 | PS |
| 339 | SP://Q32625 | atCg00490 | RBL_MORRU Ribulose bisphosphate carboxylase large chain (Fragment) OS=Morus rubra GN=rbcL PE=3 SV=1 | 3 | 0.676 | 0.167 | 0.002 | PS |
| 340 | SP://Q9BBU1 | atCg00490 | RBL_LOTJA Ribulose bisphosphate carboxylase large chain OS=Lotus japonicus GN=rbcL PE=3 SV=1 | 3 | 0.676 | 0.167 | 0.002 | PS |
| 341 | SP://Q9MTE7 | atCg00490 | RBL_AVECA Ribulose bisphosphate carboxylase large chain (Fragment) OS=Averrhoa carambola GN=rbcL PE=3 SV=1 | 3 | 0.676 | 0.167 | 0.002 | PS |
| 342 | SP://P48491 | at3g55440 | TPIS_ARatH Triosephosphate isomerase_ cytosolic OS=Arabidopsis thaliana GN=CTIMC PE=1 SV=2 | 2 | 0.675 | 0.298 | 0.037 | glycolysis |
| 343 | SP://B2LMK1 | atCg00490 | RBL_GUIAB Ribulose bisphosphate carboxylase large chain OS=Guizotia abyssinica GN=rbcL PE=3 SV=1 | 3 | 0.673 | 0.185 | 0.002 | PS |
| 344 | SP://O19989 | atCg00490 | RBL_BULAR Ribulose bisphosphate carboxylase large chain (Fragment) OS=Bulnesia arborea GN=rbcL PE=3 SV=1 | 3 | 0.673 | 0.185 | 0.002 | PS |
| 345 | SP://P19161 | atCg00490 | RBL_FLABI Ribulose bisphosphate carboxylase large chain OS=Flaveria bidentis GN=rbcL PE=3 SV=1 | 3 | 0.673 | 0.185 | 0.002 | PS |
| 346 | SP://P19162 | atCg00490 | RBL_FLAPR Ribulose bisphosphate carboxylase large chain OS=Flaveria pringlei GN=rbcL PE=3 SV=1 | 3 | 0.673 | 0.185 | 0.002 | PS |
| 347 | SP://P45738 | atCg00490 | RBL_HELAN Ribulose bisphosphate carboxylase large chain OS=Helianthus annuus GN=rbcL PE=3 SV=2 | 3 | 0.673 | 0.185 | 0.002 | PS |
| 348 | SP://P48693 | atCg00490 | RBL_CICIN Ribulose bisphosphate carboxylase large chain OS=Cichorium intybus GN=rbcL PE=3 SV=1 | 3 | 0.673 | 0.185 | 0.002 | PS |
| 349 | SP://Q37192 | atCg00490 | RBL_BARSO Ribulose bisphosphate carboxylase large chain OS=Bartlettina sordida GN=rbcL PE=3 SV=1 | 3 | 0.673 | 0.185 | 0.002 | PS |
| 350 | SP://O20346 | atCg00490 | RBL_CLITE Ribulose bisphosphate carboxylase large chain (Fragment) OS=Clitoria ternatea GN=rbcL PE=3 SV=1 | 4 | 0.672 | 0.159 | 0.003 | PS |
| 351 | SP://O62970 | atCg00490 | RBL_GLYEC Ribulose bisphosphate carboxylase large chain (Fragment) OS=Glycyrrhiza echinata GN=rbcL PE=3 SV=1 | 4 | 0.672 | 0.159 | 0.003 | PS |
| 352 | SP://P27064 | atCg00490 | RBL_CUCSA Ribulose bisphosphate carboxylase large chain OS=Cucumis sativus GN=rbcL PE=1 SV=5 | 4 | 0.672 | 0.159 | 0.003 | PS |
| 353 | SP://P28394 | atCg00490 | RBL_CORMY Ribulose bisphosphate carboxylase large chain (Fragment) OS=Coriaria myrtifolia GN=rbcL PE=3 SV=1 | 4 | 0.672 | 0.159 | 0.003 | PS |
| 354 | SP://P28399 | atCg00490 | RBL_DIGPU Ribulose bisphosphate carboxylase large chain OS=Digitalis purpurea GN=rbcL PE=3 SV=2 | 4 | 0.672 | 0.159 | 0.003 | PS |
| 355 | SP://P28419 | atCg00490 | RBL_HAMMO Ribulose bisphosphate carboxylase large chain (Fragment) OS=Hamamelis mollis GN=rbcL PE=3 SV=1 | 4 | 0.672 | 0.159 | 0.003 | PS |
| 356 | SP://P28436 | atCg00490 | RBL_OXADI Ribulose bisphosphate carboxylase large chain (Fragment) OS=Oxalis dillenii GN=rbcL PE=3 SV=2 | 4 | 0.672 | 0.159 | 0.003 | PS |
| 357 | SP://P28454 | atCg00490 | RBL_SECDI Ribulose bisphosphate carboxylase large chain (Fragment) OS=Securidaca diversifolia GN=rbcL PE=3 SV=2 | 4 | 0.672 | 0.159 | 0.003 | PS |
| 358 | SP://P28459 | atCg00490 | RBL_VIBAC Ribulose bisphosphate carboxylase large chain OS=Viburnum acerifolium GN=rbcL PE=3 SV=1 | 4 | 0.672 | 0.159 | 0.003 | PS |
| 359 | SP://P48706 | atCg00490 | RBL_LACSA Ribulose bisphosphate carboxylase large chain OS=Lactuca sativa GN=rbcL PE=3 SV=2 | 4 | 0.672 | 0.159 | 0.003 | PS |
| 360 | SP://Q01873 | atCg00490 | RBL_LIQST Ribulose bisphosphate carboxylase large chain OS=Liquidambar styraciflua GN=rbcL PE=3 SV=1 | 4 | 0.672 | 0.159 | 0.003 | PS |
| 361 | SP://Q05800 | atCg00490 | RBL_NELLU Ribulose bisphosphate carboxylase large chain (Fragment) OS=Nelumbo lutea GN=rbcL PE=3 SV=1 | 4 | 0.672 | 0.159 | 0.003 | PS |
| 362 | SP://Q31655 | atCg00490 | RBL_AJUCH Ribulose bisphosphate carboxylase large chain (Fragment) OS=Ajuga chamaepitys GN=rbcL PE=3 SV=1 | 4 | 0.672 | 0.159 | 0.003 | PS |
| 363 | SP://Q33245 | atCg00490 | RBL_ULMAL Ribulose bisphosphate carboxylase large chain (Fragment) OS=Ulmus alata GN=rbcL PE=3 SV=1 | 4 | 0.672 | 0.159 | 0.003 | PS |
| 364 | SP://Q33438 | atCg00490 | RBL_ERYCG Ribulose bisphosphate carboxylase large chain (Fragment) OS=Erythrina crista_galli GN=rbcL PE=3 SV=1 | 4 | 0.672 | 0.159 | 0.003 | PS |
| 365 | SP://Q9T4F2 | atCg00490 | RBL_NEPOL Ribulose bisphosphate carboxylase large chain OS=Nephroselmis olivacea GN=rbcL_A PE=3 SV=1 | 2 | 0.668 | 0.200 | 0.015 | PS |
| 366 | SP://P12466 | atCg00490 | RBL_CHLVU Ribulose bisphosphate carboxylase large chain OS=Chlorella vulgaris GN=rbcL PE=3 SV=1 | 3 | 0.668 | 0.168 | 0.019 | PS |
| 367 | SP://P34915 | atCg00490 | RBL_PHYPA Ribulose bisphosphate carboxylase large chain OS=Physcomitrella patens subsp. patens GN=rbcL PE=1 SV=2 | 3 | 0.668 | 0.168 | 0.019 | PS |
| 368 | SP://Q06SI3 | atCg00490 | RBL_STIHE Ribulose bisphosphate carboxylase large chain OS=Stigeoclonium helveticum GN=rbcL PE=3 SV=1 | 3 | 0.668 | 0.168 | 0.019 | PS |

| | | | | | | | | |
|---|---|---|---|---|---|---|---|---|
| 369 | SP://Q33619 | atCg00490 | RBL_MONDI Ribulose bisphosphate carboxylase large chain (Fragment) OS=Monarda didyma GN=rbcL PE=3 SV=1 | 3 | 0.668 | 0.168 | 0.019 | PS |
| 370 | SP://Q3ZJ74 | atCg00490 | RBL_PSEAK Ribulose bisphosphate carboxylase large chain OS=Pseudendoclonium akinetum GN=rbcL PE=3 SV=2 | 3 | 0.668 | 0.168 | 0.019 | PS |
| 371 | SP://Q9SCB9 | at1g12840 | VatC_HORVU V_type proton atPase subunit C OS=Hordeum vulgare GN=VatC PE=1 SV=1 | 5 | 0.668 | 0.201 | 0.013 | transport |
| 372 | SP://Q7F9I1 | at5g50920 | CLPC1_ORYSJ Chaperone protein ClpC1_ chloroplastic OS=Oryza sativa subsp. japonica GN=CLPC1 PE=2 SV=2 | 8 | 0.667 | 0.197 | 0.035 | protein |
| 373 | SP://Q9SH76 | at2g07560 | PMA6_ARatH atPase 6_ plasma membrane_type OS=Arabidopsis thaliana GN=AHA6 PE=2 SV=1 | 8 | 0.667 | 0.116 | 0.000 | transport |
| 374 | SP://Q8W1L6 | at4g29010 | MFP_ORYSJ Peroxisomal fatty acid beta_oxidation multifunctional protein OS=Oryza sativa subsp. japonica GN=MFP | 6 | 0.666 | 0.115 | 0.001 | lipid metabolism |
| 375 | SP://P26518 | at1g13440 | G3PC_MAGLI Glyceraldehyde_3_phosphate dehydrogenase_ cytosolic OS=Magnolia liliiflora GN=GAPC PE=2 SV=1 | 5 | 0.665 | 0.117 | 0.000 | glycolysis |
| 376 | SP://P28428 | atCg00490 | RBL_JUSOD Ribulose bisphosphate carboxylase large chain (Fragment) OS=Justicia odora GN=rbcL PE=3 SV=1 | 4 | 0.665 | 0.161 | 0.020 | PS |
| 377 | SP://Q33600 | atCg00490 | RBL_LAVLA Ribulose bisphosphate carboxylase large chain (Fragment) OS=Lavandula lanata GN=rbcL PE=3 SV=1 | 4 | 0.665 | 0.161 | 0.020 | PS |
| 378 | SP://P28449 | atCg00490 | RBL_RUTFR Ribulose bisphosphate carboxylase large chain (Fragment) OS=Ruttya fruticosa GN=rbcL PE=3 SV=1 | 3 | 0.664 | 0.187 | 0.019 | PS |
| 379 | SP://P12628 | at5g25880 | MAOX_PHAVU NADP_dependent malic enzyme OS=Phaseolus vulgaris GN=ME1 PE=2 SV=1 | 5 | 0.664 | 0.051 | 0.000 | TCA |
| 380 | SP://P34937 | at3g55440 | TPIS_HORVU Triosephosphate isomerase_ cytosolic OS=Hordeum vulgare PE=1 SV=3 | 9 | 0.664 | 0.108 | 0.000 | glycolysis |
| 381 | SP://P34105 | at1g79750 | MAOX_POPTR NADP_dependent malic enzyme OS=Populus trichocarpa PE=2 SV=3 | 3 | 0.662 | 0.052 | 0.000 | TCA |
| 382 | SP://Q9AYT7 | at2g05830 | MTNA_HORVU Methylthioribose_1_phosphate isomerase OS=Hordeum vulgare GN=IDI2 PE=2 SV=1 | 3 | 0.662 | 0.180 | 0.029 | protein |
| 383 | SP://P27066 | atCg00490 | RBL_SOYBN Ribulose bisphosphate carboxylase large chain OS=Glycine max GN=rbcL PE=1 SV=3 | 4 | 0.659 | 0.162 | 0.002 | PS |
| 384 | SP://Q9LV11 | at5g62670 | PMA11_ARatH atPase 11_ plasma membrane_type OS=Arabidopsis thaliana GN=AHA11 PE=1 SV=1 | 7 | 0.659 | 0.151 | 0.001 | transport |
| 385 | SP://P83970 | at4g30190 | PMA1_WHEat Plasma membrane atPase OS=Triticum aestivum GN=ha1 PE=2 SV=1 | 24 | 0.657 | 0.072 | 0.000 | transport |
| 386 | SP://Q42908 | at1g09780 | PMGI_MESCR 2_3_bisphosphoglycerate_independent phosphoglycerate mutase OS=Mesembryanthemum crystallinum | 7 | 0.654 | 0.172 | 0.008 | glycolysis |
| 387 | SP://P42730 | at1g74310 | CLPB1_ARatH Chaperone protein ClpB1 OS=Arabidopsis thaliana GN=CLPB1 PE=1 SV=2 | 8 | 0.649 | 0.131 | 0.000 | stress |
| 388 | SP://Q6F2Y7 | at1g74310 | CLPB1_ORYSJ Chaperone protein ClpB1 OS=Oryza sativa subsp. japonica GN=CLPB1 PE=2 SV=1 | 15 | 0.649 | 0.079 | 0.000 | stress |
| 389 | SP://P26517 | at3g04120 | G3PC1_HORVU Glyceraldehyde_3_phosphate dehydrogenase 1_ cytosolic OS=Hordeum vulgare GN=GAPC PE=2 SV=1 | 13 | 0.648 | 0.077 | 0.000 | glycolysis |
| 390 | SP://Q1KVV0 | atCg00490 | RBL_ACUOB Ribulose bisphosphate carboxylase large chain OS=Acutodesmus obliquus GN=rbcL PE=3 SV=1 | 4 | 0.648 | 0.156 | 0.013 | PS |
| 391 | SP://Q9MUT6 | atCg00490 | RBL_MESVI Ribulose bisphosphate carboxylase large chain OS=Mesostigma viride GN=rbcL PE=3 SV=1 | 4 | 0.648 | 0.156 | 0.013 | PS |
| 392 | SP://P21820 | at3g55440 | TPIS_COPJA Triosephosphate isomerase_ cytosolic OS=Coptis japonica PE=2 SV=1 | 3 | 0.647 | 0.151 | 0.001 | glycolysis |
| 393 | SP://O24466 | at3g53610 | RAE1A_ARatH Ras_related protein RABE1a OS=Arabidopsis thaliana GN=RABE1A PE=1 SV=1 | 2 | 0.645 | 0.206 | 0.007 | signalling |
| 394 | SP://P28186 | at3g46060 | RAE1C_ARatH Ras_related protein RABE1c OS=Arabidopsis thaliana GN=RABE1C PE=1 SV=1 | 2 | 0.645 | 0.206 | 0.007 | signalling |
| 395 | SP://P31584 | at1g02130 | YPTV1_VOLCA GTP_binding protein yptV1 OS=Volvox carteri GN=YPTV1 PE=3 SV=1 | 2 | 0.645 | 0.206 | 0.007 | signalling |
| 396 | SP://Q39433 | at3g46060 | RB1BV_BETVU Ras_related protein RAB1BV OS=Beta vulgaris GN=RAB1BV PE=2 SV=1 | 2 | 0.645 | 0.206 | 0.007 | signalling |
| 397 | SP://Q9LZD4 | at5g03520 | RAE1D_ARatH Ras_related protein RABE1d OS=Arabidopsis thaliana GN=RABE1D PE=1 SV=1 | 2 | 0.645 | 0.206 | 0.007 | signalling |
| 398 | SP://Q9SF91 | at3g09900 | RAE1E_ARatH Ras_related protein RABE1e OS=Arabidopsis thaliana GN=RABE1E PE=1 SV=1 | 2 | 0.645 | 0.206 | 0.007 | signalling |
| 399 | SP://Q7SIC9 | at3g60750 | TKTC_MAIZE Transketolase_ chloroplastic OS=Zea mays PE=1 SV=1 | 5 | 0.644 | 0.134 | 0.000 | PS |
| 400 | SP://O23755 | at1g56070 | EF2_BETVU Elongation factor 2 OS=Beta vulgaris PE=2 SV=1 | 13 | 0.643 | 0.122 | 0.032 | protein |
| 401 | SP://P28188 | at1g02130 | RAD2A_ARatH Ras_related protein RABD2a OS=Arabidopsis thaliana GN=RABD2A PE=1 SV=3 | 3 | 0.643 | 0.204 | 0.019 | signalling |
| 402 | SP://Q9M2A0 | at3g42640 | PMA8_ARatH atPase 8_ plasma membrane_type OS=Arabidopsis thaliana GN=AHA8 PE=3 SV=1 | 3 | 0.640 | 0.212 | 0.013 | transport |

| | | | | | | | | |
|---|---|---|---|---|---|---|---|---|
| 403 | SP://A8W3D6 | atCg00490 | RBL_CUSEX Ribulose bisphosphate carboxylase large chain OS=Cuscuta exaltata GN=rbcL PE=3 SV=1 | 4 | 0.640 | 0.161 | 0.001 | PS |
| 404 | SP://P30401 | atCg00490 | RBL_CUSRE Ribulose bisphosphate carboxylase large chain OS=Cuscuta reflexa GN=rbcL PE=3 SV=1 | 4 | 0.640 | 0.161 | 0.001 | PS |
| 405 | SP://P69572 | atCg00490 | RBL_LUPAA Ribulose bisphosphate carboxylase large chain (Fragment) OS=Lupinus atlanticus GN=rbcL PE=3 SV=1 | 4 | 0.640 | 0.161 | 0.001 | PS |
| 406 | SP://P69573 | atCg00490 | RBL_LUPAB Ribulose bisphosphate carboxylase large chain (Fragment) OS=Lupinus albifrons GN=rbcL PE=3 SV=1 | 4 | 0.640 | 0.161 | 0.001 | PS |
| 407 | SP://P69574 | atCg00490 | RBL_LUPAE Ribulose bisphosphate carboxylase large chain (Fragment) OS=Lupinus albescens GN=rbcL PE=3 SV=1 | 4 | 0.640 | 0.161 | 0.001 | PS |
| 408 | SP://P69575 | atCg00490 | RBL_LUPAN Ribulose bisphosphate carboxylase large chain (Fragment) OS=Lupinus angustifolius GN=rbcL PE=3 SV=1 | 4 | 0.640 | 0.161 | 0.001 | PS |
| 409 | SP://P69576 | atCg00490 | RBL_LUPAR Ribulose bisphosphate carboxylase large chain (Fragment) OS=Lupinus arboreus GN=rbcL PE=3 SV=1 | 4 | 0.640 | 0.161 | 0.001 | PS |
| 410 | SP://P69579 | atCg00490 | RBL_LUPCO Ribulose bisphosphate carboxylase large chain (Fragment) OS=Lupinus cosentinii GN=rbcL PE=3 SV=1 | 4 | 0.640 | 0.161 | 0.001 | PS |
| 411 | SP://P92397 | atCg00490 | RBL_LUPAL Ribulose bisphosphate carboxylase large chain (Fragment) OS=Lupinus albus GN=rbcL PE=3 SV=1 | 4 | 0.640 | 0.161 | 0.001 | PS |
| 412 | SP://P92401 | atCg00490 | RBL_LUPDE Ribulose bisphosphate carboxylase large chain (Fragment) OS=Lupinus microcarpus var. densiflorus GN=rbcL | 4 | 0.640 | 0.161 | 0.001 | PS |
| 413 | SP://P92406 | atCg00490 | RBL_LUPMI Ribulose bisphosphate carboxylase large chain (Fragment) OS=Lupinus microcarpus GN=rbcL PE=3 SV=1 | 4 | 0.640 | 0.161 | 0.001 | PS |
| 414 | SP://P92407 | atCg00490 | RBL_LUPNA Ribulose bisphosphate carboxylase large chain (Fragment) OS=Lupinus nanus GN=rbcL PE=3 SV=1 | 4 | 0.640 | 0.161 | 0.001 | PS |
| 415 | SP://P52877 | at2g17630 | SERC_SPIOL Phosphoserine aminotransferase_ chloroplastic OS=Spinacia oleracea PE=2 SV=1 | 2 | 0.639 | 0.250 | 0.045 | amino acid metabolism |
| 416 | SP://Q9SU58 | at3g47950 | PMA4_ARatH atPase 4_ plasma membrane_type OS=Arabidopsis thaliana GN=AHA4 PE=2 SV=2 | 5 | 0.636 | 0.193 | 0.010 | transport |
| 417 | SP://P36863 | at4g17170 | YPTV4_VOLCA GTP_binding protein yptV4 OS=Volvox carteri GN=YPTV4 PE=3 SV=1 | 6 | 0.634 | 0.180 | 0.006 | signalling |
| 418 | SP://Q39570 | at4g17170 | YPTC4_CHLRE GTP_binding protein YPTC4 OS=Chlamydomonas reinhardtii GN=YPTC4 PE=3 SV=1 | 6 | 0.634 | 0.180 | 0.006 | signalling |
| 419 | SP://P92963 | at4g17170 | RAB1C_ARatH Ras_related protein RABB1c OS=Arabidopsis thaliana GN=RABB1C PE=1 SV=1 | 8 | 0.634 | 0.172 | 0.008 | signalling |
| 420 | SP://Q9SLX0 | at3g06720 | IMA1B_ORYSJ Importin subunit alpha_1b OS=Oryza sativa subsp. japonica GN=Os05g0155500 PE=1 SV=2 | 9 | 0.633 | 0.188 | 0.009 | protein |
| 421 | SP://O82191 | at2g19900 | MAOP1_ARatH NADP_dependent malic enzyme 1 OS=Arabidopsis thaliana GN=NADP_ME1 PE=1 SV=1 | 2 | 0.630 | 0.238 | 0.047 | TCA |
| 422 | SP://A2XAZ3 | at5g43940 | ADHX_ORYSI Alcohol dehydrogenase class_3 OS=Oryza sativa subsp. indica GN=ADHIII PE=3 SV=1 | 3 | 0.627 | 0.176 | 0.010 | misc |
| 423 | SP://Q0DWH1 | at5g43940 | ADHX_ORYSJ Alcohol dehydrogenase class_3 OS=Oryza sativa subsp. japonica GN=Os02g0815500 PE=2 SV=1 | 3 | 0.627 | 0.176 | 0.010 | misc |
| 424 | SP://Q7XPY2 | at4g30190 | PMA1_ORYSJ Plasma membrane atPase OS=Oryza sativa subsp. japonica GN=Os04g0656100 PE=2 SV=1 | 8 | 0.626 | 0.178 | 0.013 | transport |
| 425 | SP://Q7M290 | at3g42640 | PMA_AVESA Plasma membrane atPase (Fragments) OS=Avena sativa PE=1 SV=2 | 4 | 0.624 | 0.163 | 0.001 | transport |
| 426 | SP://Q38922 | at4g35860 | RAB1B_ARatH Ras_related protein RABB1b OS=Arabidopsis thaliana GN=RABB1B PE=2 SV=1 | 5 | 0.624 | 0.191 | 0.006 | signalling |
| 427 | SP://Q9ZRE2 | at3g11730 | RABD1_ARatH Ras_related protein RABD1 OS=Arabidopsis thaliana GN=RABD1 PE=1 SV=1 | 2 | 0.623 | 0.204 | 0.005 | signalling |
| 428 | SP://O04499 | at1g09780 | PMG1_ARatH 2_3_bisphosphoglycerate_independent phosphoglycerate mutase 1 OS=Arabidopsis thaliana GN=PGM1 | 6 | 0.621 | 0.175 | 0.020 | glycolysis |
| 429 | SP://Q9M9K1 | at3g08590 | PMG2_ARatH Probable 2_3_bisphosphoglycerate_independent phosphoglycerate mutase 2 OS=Arabidopsis thaliana | 7 | 0.620 | 0.152 | 0.004 | glycolysis |
| 430 | SP://O24246 | at1g09780 | PMGI_PRUDU 2_3_bisphosphoglycerate_independent phosphoglycerate mutase (Fragment) OS=Prunus dulcis PE=2 SV=1 | 5 | 0.619 | 0.179 | 0.015 | glycolysis |
| 431 | SP://Q67UX7 | at4g31750 | P2C10_ORYSJ Probable protein phosphatase 2C 10 OS=Oryza sativa subsp. japonica GN=Os02g0149800 PE=2 SV=1 | 3 | 0.615 | 0.178 | 0.005 | protein |
| 432 | SP://P42896 | at2g36530 | ENO_RICCO Enolase OS=Ricinus communis PE=2 SV=1 | 7 | 0.615 | 0.172 | 0.000 | glycolysis |
| 433 | SP://P30792 | at3g08590 | PMGI_MAIZE 2_3_bisphosphoglycerate_independent phosphoglycerate mutase OS=Zea mays PE=1 SV=1 | 8 | 0.605 | 0.181 | 0.017 | glycolysis |
| 434 | SP://Q2QLY4 | at5g17920 | METE2_ORYSJ 5_methyltetrahydropteroyltriglutamate__homocysteine methyltransferase 2 OS=Oryza sativa subsp. japonica | 16 | 0.604 | 0.071 | 0.000 | amino acid metabolism |
| 435 | SP://P93804 | at1g70730 | PGMC1_MAIZE Phosphoglucomutase_ cytoplasmic 1 OS=Zea mays PE=2 SV=2 | 2 | 0.604 | 0.347 | 0.039 | glycolysis |
| 436 | SP://P93805 | at1g70730 | PGMC2_MAIZE Phosphoglucomutase_ cytoplasmic 2 OS=Zea mays PE=2 SV=2 | 2 | 0.604 | 0.347 | 0.039 | glycolysis |

| | | | | | | | | |
|---|---|---|---|---|---|---|---|---|
| 437 | SP://P20973 | at5g06460 | UBE11_WHEat Ubiquitin_activating enzyme E1 1 OS=Triticum aestivum GN=UBA1 PE=1 SV=1 | 20 | 0.604 | 0.056 | 0.000 | protein |
| 438 | SP://Q2QLY5 | at5g17920 | METE1_ORYSJ 5_methyltetrahydropteroyltriglutamate__homocysteine methyltransferase 1 OS=Oryza sativa subsp. japonica | 18 | 0.603 | 0.071 | 0.000 | amino acid metabolism |
| 439 | SP://P31251 | at5g06460 | UBE12_WHEat Ubiquitin_activating enzyme E1 2 OS=Triticum aestivum GN=UBA2 PE=2 SV=1 | 19 | 0.599 | 0.062 | 0.000 | protein |
| 440 | SP://Q5VQ78 | at3g15980 | COB21_ORYSJ Coatomer subunit beta__1 OS=Oryza sativa subsp. japonica GN=Os06g0143900 PE=2 SV=1 | 6 | 0.595 | 0.176 | 0.006 | cell.vesicle transport |
| 441 | SP://O23561 | at4g17160 | RAB1A_ARatH Ras_related protein RABB1a OS=Arabidopsis thaliana GN=RABB1A PE=2 SV=1 | 2 | 0.595 | 0.281 | 0.012 | signalling |
| 442 | SP://Q9SNX2 | at1g70730 | PGMC_BROIN Phosphoglucomutase_ cytoplasmic OS=Bromus inermis GN=PGM1 PE=2 SV=1 | 11 | 0.594 | 0.153 | 0.004 | glycolysis |
| 443 | SP://P26520 | at1g13440 | G3PC_PETHY Glyceraldehyde_3_phosphate dehydrogenase_ cytosolic OS=Petunia hybrida GN=GAPC PE=2 SV=1 | 6 | 0.592 | 0.090 | 0.000 | glycolysis |
| 444 | SP://O03986 | at5g56000 | HS904_ARatH Heat shock protein 90_4 OS=Arabidopsis thaliana GN=HSP90_4 PE=1 SV=1 | 8 | 0.592 | 0.133 | 0.027 | stress |
| 445 | SP://P51818 | at5g56010 | HS903_ARatH Heat shock protein 90_3 OS=Arabidopsis thaliana GN=HSP90_3 PE=1 SV=2 | 8 | 0.592 | 0.133 | 0.027 | stress |
| 446 | SP://P55737 | at5g56030 | HS902_ARatH Heat shock protein 90_2 OS=Arabidopsis thaliana GN=HSP90_2 PE=1 SV=1 | 8 | 0.592 | 0.133 | 0.027 | stress |
| 447 | SP://O20250 | at2g45290 | TKTC_SPIOL Transketolase_ chloroplastic OS=Spinacia oleracea PE=1 SV=1 | 4 | 0.591 | 0.168 | 0.000 | PS |
| 448 | SP://Q8VZH2 | at4g33090 | APM1_ARatH Aminopeptidase M1 OS=Arabidopsis thaliana GN=APM1 PE=1 SV=1 | 2 | 0.591 | 0.177 | 0.003 | protein |
| 449 | SP://P93262 | at1g70730 | PGMC_MESCR Phosphoglucomutase_ cytoplasmic OS=Mesembryanthemum crystallinum GN=PGM1 PE=2 SV=1 | 2 | 0.589 | 0.308 | 0.013 | glycolysis |
| 450 | SP://Q9M4G4 | at1g70730 | PGMC_SOLTU Phosphoglucomutase_ cytoplasmic OS=Solanum tuberosum GN=PGM1 PE=2 SV=1 | 2 | 0.589 | 0.308 | 0.013 | glycolysis |
| 451 | SP://Q9SM60 | at1g70730 | PGMC_PEA Phosphoglucomutase_ cytoplasmic OS=Pisum sativum GN=PGM1 PE=2 SV=1 | 2 | 0.589 | 0.308 | 0.013 | glycolysis |
| 452 | SP://Q9ZSQ4 | at1g70730 | PGMC_POPTN Phosphoglucomutase_ cytoplasmic OS=Populus tremula GN=PGM1 PE=2 SV=1 | 2 | 0.589 | 0.308 | 0.013 | glycolysis |
| 453 | SP://Q06572 | at1g15690 | AVP_HORVU Pyrophosphate_energized vacuolar membrane proton pump OS=Hordeum vulgare PE=2 SV=2 | 6 | 0.589 | 0.135 | 0.000 | transport |
| 454 | SP://P21616 | at1g15690 | AVP_VIGRR Pyrophosphate_energized vacuolar membrane proton pump OS=Vigna radiata var. radiata PE=1 SV=4 | 5 | 0.589 | 0.140 | 0.000 | transport |
| 455 | SP://Q9LYG3 | at5g11670 | MAOP2_ARatH NADP_dependent malic enzyme 2 OS=Arabidopsis thaliana GN=NADP_ME2 PE=1 SV=1 | 3 | 0.587 | 0.221 | 0.036 | TCA |
| 456 | SP://A7M906 | atCg00490 | RBL_CUSGR Ribulose bisphosphate carboxylase large chain OS=Cuscuta gronovii GN=rbcL PE=3 SV=1 | 2 | 0.587 | 0.272 | 0.011 | PS |
| 457 | SP://A8W3J2 | atCg00490 | RBL_CUSOB Ribulose bisphosphate carboxylase large chain OS=Cuscuta obtusiflora GN=rbcL PE=3 SV=1 | 2 | 0.587 | 0.272 | 0.011 | PS |
| 458 | SP://P25830 | atCg00490 | RBL_CERGL Ribulose bisphosphate carboxylase large chain OS=Cerastium glomeratum GN=rbcL PE=3 SV=1 | 2 | 0.587 | 0.272 | 0.011 | PS |
| 459 | SP://P28388 | atCg00490 | RBL_CALPL Ribulose bisphosphate carboxylase large chain (Fragment) OS=Caltha palustris GN=rbcL PE=3 SV=1 | 2 | 0.587 | 0.272 | 0.011 | PS |
| 460 | SP://P28429 | atCg00490 | RBL_LOBSP Ribulose bisphosphate carboxylase large chain (Fragment) OS=Lobelia sp. GN=rbcL PE=3 SV=2 | 2 | 0.587 | 0.272 | 0.011 | PS |
| 461 | SP://P28460 | atCg00490 | RBL_VITAE Ribulose bisphosphate carboxylase large chain (Fragment) OS=Vitis aestivalis GN=rbcL PE=3 SV=1 | 2 | 0.587 | 0.272 | 0.011 | PS |
| 462 | SP://P36479 | atCg00490 | RBL_ASACA Ribulose bisphosphate carboxylase large chain (Fragment) OS=Asarum canadense GN=rbcL PE=3 SV=1 | 2 | 0.587 | 0.272 | 0.011 | PS |
| 463 | SP://P46820 | atCg00490 | RBL_STEME Ribulose bisphosphate carboxylase large chain OS=Stellaria media GN=rbcL PE=3 SV=1 | 2 | 0.587 | 0.272 | 0.011 | PS |
| 464 | SP://P56648 | atCg00490 | RBL_VITVI Ribulose bisphosphate carboxylase large chain OS=Vitis vinifera GN=rbcL PE=3 SV=2 | 2 | 0.587 | 0.272 | 0.011 | PS |
| 465 | SP://Q05554 | atCg00490 | RBL_ANTMA Ribulose bisphosphate carboxylase large chain (Fragment) OS=Antirrhinum majus GN=rbcL PE=3 SV=1 | 2 | 0.587 | 0.272 | 0.011 | PS |
| 466 | SP://Q05988 | atCg00490 | RBL_CONTR Ribulose bisphosphate carboxylase large chain (Fragment) OS=Convolvulus tricolor GN=rbcL PE=3 SV=1 | 2 | 0.587 | 0.272 | 0.011 | PS |
| 467 | SP://Q05992 | atCg00490 | RBL_POLRE Ribulose bisphosphate carboxylase large chain (Fragment) OS=Polemonium reptans GN=rbcL PE=3 SV=1 | 2 | 0.587 | 0.272 | 0.011 | PS |
| 468 | SP://Q43848 | at3g60750 | TKTC_SOLTU Transketolase_ chloroplastic OS=Solanum tuberosum PE=2 SV=1 | 3 | 0.587 | 0.193 | 0.002 | PS |
| 469 | SP://Q6Z6L4 | at4g33090 | APM1A_ORYSJ Aminopeptidase M1_A OS=Oryza sativa subsp. japonica GN=Os02g0218200 PE=2 SV=1 | 8 | 0.586 | 0.132 | 0.006 | protein |
| 470 | SP://P26519 | at3g04120 | G3PC_PETCR Glyceraldehyde_3_phosphate dehydrogenase_ cytosolic OS=Petroselinum crispum GN=GAPC PE=2 SV=1 | 3 | 0.585 | 0.250 | 0.025 | glycolysis |

| | | | | | | | | |
|---|---|---|---|---|---|---|---|---|
| 471 | SP://Q75HJ0 | at2g42520 | RH37_ORYSJ DEAD_box atP_dependent RNA helicase 37 OS=Oryza sativa subsp. japonica GN=PL10A PE=2 SV=1 | 3 | 0.584 | 0.232 | 0.017 | RNA |
| 472 | SP://Q6AVI0 | at3g42170 | RSLE2_ORYSJ Zinc finger BED domain_containing protein RICESLEEPER 2 OS=Oryza sativa subsp. | 3 | 0.583 | 0.228 | 0.020 | DNA |
| 473 | SP://Q9SU63 | at3g48000 | AL2B4_ARatH Aldehyde dehydrogenase family 2 member B4_ mitochondrial OS=Arabidopsis thaliana GN=ALDH2B4 | 2 | 0.582 | 0.334 | 0.029 | fermentation |
| 474 | SP://O49299 | at1g23190 | PGMC1_ARatH Probable phosphoglucomutase_ cytoplasmic 1 OS=Arabidopsis thaliana GN=at1g23190 PE=2 SV=2 | 3 | 0.582 | 0.304 | 0.029 | glycolysis |
| 475 | SP://Q9SGC1 | at1g70730 | PGMC2_ARatH Probable phosphoglucomutase_ cytoplasmic 2 OS=Arabidopsis thaliana GN=at1g70730 PE=2 SV=1 | 3 | 0.582 | 0.304 | 0.029 | glycolysis |
| 476 | SP://Q05737 | at1g02130 | YPTM2_MAIZE GTP_binding protein YPTM2 OS=Zea mays GN=YPTM2 PE=2 SV=1 | 3 | 0.581 | 0.154 | 0.001 | signalling |
| 477 | SP://Q39571 | at5g47200 | YPTC1_CHLRE GTP_binding protein YPTC1 OS=Chlamydomonas reinhardtii GN=YPTC1 PE=3 SV=1 | 3 | 0.581 | 0.154 | 0.001 | signalling |
| 478 | SP://Q9FPJ4 | at5g47200 | RAD2B_ARatH Ras_related protein RABD2b OS=Arabidopsis thaliana GN=RABD2B PE=2 SV=1 | 3 | 0.581 | 0.154 | 0.001 | signalling |
| 479 | SP://Q9SEH3 | at4g17530 | RAD2C_ARatH Ras_related protein RABD2c OS=Arabidopsis thaliana GN=RABD2C PE=2 SV=1 | 3 | 0.581 | 0.154 | 0.001 | signalling |
| 480 | SP://F4IW47 | at2g45290 | TKTC2_ARatH Transketolase_2_ chloroplastic OS=Arabidopsis thaliana GN=TKL_2 PE=1 SV=1 | 3 | 0.580 | 0.191 | 0.001 | PS |
| 481 | SP://P31414 | at1g15690 | AVP1_ARatH Pyrophosphate_energized vacuolar membrane proton pump 1 OS=Arabidopsis thaliana GN=AVP1 PE=1 SV=1 | 4 | 0.579 | 0.166 | 0.002 | transport |
| 482 | SP://Q940P8 | at5g20890 | TCPB_ARatH T_complex protein 1 subunit beta OS=Arabidopsis thaliana GN=CCT2 PE=1 SV=1 | 3 | 0.576 | 0.193 | 0.003 | protein |
| 483 | SP://P34924 | at1g13440 | G3PC_PINSY Glyceraldehyde_3_phosphate dehydrogenase_ cytosolic OS=Pinus sylvestris GN=GAPC PE=2 SV=1 | 3 | 0.575 | 0.286 | 0.042 | glycolysis |
| 484 | SP://Q40024 | at3g48170 | BADH_HORVU Betaine aldehyde dehydrogenase OS=Hordeum vulgare PE=2 SV=1 | 5 | 0.573 | 0.332 | 0.043 | secondary metabolism |
| 485 | SP://P51615 | at5g25880 | MAOX_VITVI NADP_dependent malic enzyme OS=Vitis vinifera PE=2 SV=1 | 4 | 0.571 | 0.192 | 0.019 | TCA |
| 486 | SP://O49845 | at3g43190 | SUS2_DAUCA Sucrose synthase isoform 2 OS=Daucus carota PE=2 SV=1 | 3 | 0.570 | 0.203 | 0.003 | major CHO metabolism |
| 487 | SP://P25858 | at3g04120 | G3PC1_ARatH Glyceraldehyde_3_phosphate dehydrogenase GAPC1_ cytosolic OS=Arabidopsis thaliana GN=GAPC1 | 7 | 0.570 | 0.101 | 0.000 | glycolysis |
| 488 | SP://Q8LAY8 | at5g10740 | P2C69_ARatH Probable protein phosphatase 2C 69 OS=Arabidopsis thaliana GN=at5g10740 PE=2 SV=1 | 2 | 0.569 | 0.246 | 0.013 | protein |
| 489 | SP://O81928 | at2g30490 | TCMO_CICAR Trans_cinnamate 4_monooxygenase OS=Cicer arietinum GN=CYP73A19 PE=2 SV=2 | 4 | 0.569 | 0.242 | 0.011 | secondary metabolism |
| 490 | SP://Q42797 | at2g30490 | TCMO_SOYBN Trans_cinnamate 4_monooxygenase OS=Glycine max GN=CYP73A11 PE=2 SV=1 | 4 | 0.569 | 0.242 | 0.011 | secondary metabolism |
| 491 | SP://Q43054 | at2g30490 | TCMO_POPKI Trans_cinnamate 4_monooxygenase OS=Populus kitakamiensis GN=CYP73A16 PE=2 SV=1 | 4 | 0.569 | 0.242 | 0.011 | secondary metabolism |
| 492 | SP://Q96423 | at2g30490 | TCMO_GLYEC Trans_cinnamate 4_monooxygenase OS=Glycyrrhiza echinata GN=CYP73A14 PE=2 SV=1 | 4 | 0.569 | 0.242 | 0.011 | secondary metabolism |
| 493 | SP://Q42677 | at2g45290 | TKT7_CRAPL Transketolase 7 OS=Craterostigma plantagineum GN=TKT7 PE=2 SV=1 | 2 | 0.568 | 0.232 | 0.007 | PS |
| 494 | SP://P40392 | at1g02130 | RIC1_ORYSJ Ras_related protein RIC1 OS=Oryza sativa subsp. japonica GN=RIC1 PE=2 SV=2 | 4 | 0.568 | 0.146 | 0.000 | signalling |
| 495 | SP://P49040 | at5g20830 | SUSY1_ARatH Sucrose synthase 1 OS=Arabidopsis thaliana GN=SUS1 PE=1 SV=3 | 3 | 0.567 | 0.218 | 0.014 | major CHO metabolism |
| 496 | SP://Q9LXL5 | at3g43190 | SUS4_ARatH Sucrose synthase 4 OS=Arabidopsis thaliana GN=SUS4 PE=1 SV=1 | 3 | 0.567 | 0.218 | 0.014 | major CHO metabolism |
| 497 | SP://P35493 | at1g09780 | PMGI_RICCO 2_3_bisphosphoglycerate_independent phosphoglycerate mutase OS=Ricinus communis PE=1 SV=2 | 6 | 0.565 | 0.143 | 0.000 | glycolysis |
| 498 | SP://P49035 | at3g43190 | SUS1_DAUCA Sucrose synthase isoform 1 OS=Daucus carota PE=2 SV=1 | 4 | 0.564 | 0.199 | 0.004 | major CHO metabolism |
| 499 | SP://Q6ZDY8 | at5g66760 | SDHA_ORYSJ Succinate dehydrogenase [ubiquinone] flavoprotein subunit_ mitochondrial OS=Oryza sativa subsp. | 7 | 0.563 | 0.155 | 0.001 | TCA |
| 500 | SP://Q0JMH0 | at5g17380 | HACL_ORYSJ 2_hydroxyacyl_CoA lyase OS=Oryza sativa subsp. japonica GN=Os01g0505400 PE=3 SV=3 | 5 | 0.563 | 0.109 | 0.000 | fermentation |
| 501 | SP://Q71VM4 | at3g06720 | IMA1A_ORYSJ Importin subunit alpha_1a OS=Oryza sativa subsp. japonica GN=Os01g0253300 PE=1 SV=2 | 5 | 0.563 | 0.223 | 0.008 | protein |
| 502 | SP://P93263 | at5g17920 | METE_MESCR 5_methyltetrahydropteroyltriglutamate__homocysteine methyltransferase OS=Mesembryanthemum crystallinum | 9 | 0.562 | 0.073 | 0.000 | amino acid metabolism |
| 503 | SP://P26521 | at1g13440 | G3PC_RANAC Glyceraldehyde_3_phosphate dehydrogenase_ cytosolic OS=Ranunculus acris GN=GAPC PE=2 SV=1 | 6 | 0.561 | 0.185 | 0.019 | glycolysis |

| | | | | | | | | |
|---|---|---|---|---|---|---|---|---|
| 504 | SP://P34783 | at1g13440 | G3P_atRNU Glyceraldehyde_3_phosphate dehydrogenase OS=atriplex nummularia PE=2 SV=1 | 6 | 0.561 | 0.114 | 0.000 | glycolysis |
| 505 | SP://O24312 | at2g30490 | TCMO_POPTM Trans_cinnamate 4_monooxygenase OS=Populus tremuloides GN=CYP73A13 PE=2 SV=1 | 3 | 0.561 | 0.275 | 0.023 | secondary metabolism |
| 506 | SP://P48522 | at2g30490 | TCMO_CatRO Trans_cinnamate 4_monooxygenase OS=Catharanthus roseus GN=CYP73A4 PE=2 SV=1 | 3 | 0.561 | 0.275 | 0.023 | secondary metabolism |
| 507 | SP://Q04468 | at2g30490 | TCMO_HELTU Trans_cinnamate 4_monooxygenase OS=Helianthus tuberosus GN=CYP73A1 PE=1 SV=1 | 3 | 0.561 | 0.275 | 0.023 | secondary metabolism |
| 508 | SP://Q43067 | at2g30490 | TCMO_PEA Trans_cinnamate 4_monooxygenase OS=Pisum sativum GN=CYP73A9 PE=2 SV=2 | 3 | 0.561 | 0.275 | 0.023 | secondary metabolism |
| 509 | SP://Q43240 | at2g30490 | TCMO_ZINVI Trans_cinnamate 4_monooxygenase OS=Zinnia violacea GN=CYP73A12 PE=2 SV=1 | 3 | 0.561 | 0.275 | 0.023 | secondary metabolism |
| 510 | SP://P08735 | at3g04120 | G3PC1_MAIZE Glyceraldehyde_3_phosphate dehydrogenase 1_ cytosolic OS=Zea mays GN=GAPC1 PE=2 SV=2 | 10 | 0.559 | 0.098 | 0.000 | glycolysis |
| 511 | SP://Q9FX54 | at1g13440 | G3PC2_ARatH Glyceraldehyde_3_phosphate dehydrogenase GAPC2_ cytosolic OS=Arabidopsis thaliana GN=GAPC2 | 6 | 0.557 | 0.107 | 0.000 | glycolysis |
| 512 | SP://P37115 | at2g30490 | TCMO_VIGRR Trans_cinnamate 4_monooxygenase OS=Vigna radiata var. radiata GN=CYP73A2 PE=1 SV=1 | 3 | 0.554 | 0.273 | 0.021 | secondary metabolism |
| 513 | SP://P10691 | at3g43190 | SUS1_SOLTU Sucrose synthase OS=Solanum tuberosum PE=2 SV=1 | 5 | 0.553 | 0.203 | 0.002 | major CHO metabolism |
| 514 | SP://P49037 | at3g43190 | SUSY_SOLLC Sucrose synthase OS=Solanum lycopersicum PE=2 SV=1 | 5 | 0.553 | 0.203 | 0.002 | major CHO metabolism |
| 515 | SP://O24581 | at5g42020 | BIP3_MAIZE Luminal_binding protein 3 OS=Zea mays GN=BIPE3 PE=2 SV=1 | 13 | 0.551 | 0.050 | 0.000 | stress |
| 516 | SP://P24067 | at5g42020 | BIP2_MAIZE Luminal_binding protein 2 OS=Zea mays GN=BIPE2 PE=1 SV=3 | 13 | 0.551 | 0.050 | 0.000 | stress |
| 517 | SP://Q9ZPX5 | at2g18450 | SDHA2_ARatH Succinate dehydrogenase [ubiquinone] flavoprotein subunit 2_ mitochondrial OS=Arabidopsis thaliana | 5 | 0.551 | 0.186 | 0.013 | TCA |
| 518 | SP://P35494 | at3g08590 | PMGI_TOBAC 2_3_bisphosphoglycerate_independent phosphoglycerate mutase OS=Nicotiana tabacum PE=2 SV=1 | 5 | 0.551 | 0.133 | 0.000 | glycolysis |
| 519 | SP://P49039 | at3g43190 | SUS2_SOLTU Sucrose synthase OS=Solanum tuberosum PE=3 SV=1 | 5 | 0.550 | 0.184 | 0.001 | major CHO metabolism |
| 520 | SP://O65026 | at3g43190 | SUSY_MEDSA Sucrose synthase OS=Medicago sativa PE=2 SV=1 | 4 | 0.550 | 0.198 | 0.004 | major CHO metabolism |
| 521 | SP://P13708 | at3g43190 | SUSY_SOYBN Sucrose synthase OS=Glycine max GN=SS PE=1 SV=2 | 5 | 0.550 | 0.189 | 0.002 | major CHO metabolism |
| 522 | SP://P31926 | at3g43190 | SUSY_VICFA Sucrose synthase OS=Vicia faba GN=SUCS PE=2 SV=1 | 5 | 0.550 | 0.189 | 0.002 | major CHO metabolism |
| 523 | SP://Q01390 | at3g43190 | SUSY_VIGRR Sucrose synthase OS=Vigna radiata var. radiata GN=SS1 PE=1 SV=1 | 5 | 0.550 | 0.189 | 0.002 | major CHO metabolism |
| 524 | SP://Q42662 | at5g17920 | METE_PLESU 5_methyltetrahydropteroyltriglutamate__homocysteine methyltransferase OS=Plectranthus scutellarioides | 12 | 0.550 | 0.072 | 0.000 | amino acid metabolism |
| 525 | SP://O04486 | at1g09630 | RAA2A_ARatH Ras_related protein RABA2a OS=Arabidopsis thaliana GN=RABA2A PE=2 SV=1 | 2 | 0.550 | 0.262 | 0.024 | signalling |
| 526 | SP://P40393 | at4g18800 | RIC2_ORYSJ Ras_related protein RIC2 OS=Oryza sativa subsp. japonica GN=RIC2 PE=2 SV=2 | 2 | 0.550 | 0.262 | 0.024 | signalling |
| 527 | SP://Q1PEX3 | at2g33870 | RAA1H_ARatH Ras_related protein RABA1h OS=Arabidopsis thaliana GN=RABA1H PE=2 SV=1 | 2 | 0.550 | 0.262 | 0.024 | signalling |
| 528 | SP://Q39222 | at1g16920 | RAA1B_ARatH Ras_related protein RABA1b OS=Arabidopsis thaliana GN=RABA1B PE=2 SV=1 | 2 | 0.550 | 0.262 | 0.024 | signalling |
| 529 | SP://Q39572 | at1g09630 | YPTC6_CHLRE Ras_related protein YPTC6 OS=Chlamydomonas reinhardtii GN=YPTC6 PE=3 SV=1 | 2 | 0.550 | 0.262 | 0.024 | signalling |
| 530 | SP://Q40523 | at1g07410 | RB11A_TOBAC Ras_related protein Rab11A OS=Nicotiana tabacum GN=RAB11A PE=2 SV=1 | 2 | 0.550 | 0.262 | 0.024 | signalling |
| 531 | SP://Q9FJH0 | at5g60860 | RAA1F_ARatH Ras_related protein RABA1f OS=Arabidopsis thaliana GN=RABA1F PE=2 SV=1 | 2 | 0.550 | 0.262 | 0.024 | signalling |
| 532 | SP://Q9FK68 | at5g45750 | RAA1C_ARatH Ras_related protein RABA1c OS=Arabidopsis thaliana GN=RABA1C PE=2 SV=1 | 2 | 0.550 | 0.262 | 0.024 | signalling |
| 533 | SP://Q9LK99 | at3g15060 | RAA1G_ARatH Ras_related protein RABA1g OS=Arabidopsis thaliana GN=RABA1G PE=2 SV=1 | 2 | 0.550 | 0.262 | 0.024 | signalling |
| 534 | SP://Q9SN35 | at4g18800 | RAA1D_ARatH Ras_related protein RABA1d OS=Arabidopsis thaliana GN=RABA1D PE=2 SV=1 | 2 | 0.550 | 0.262 | 0.024 | signalling |
| 535 | SP://Q09054 | at3g04120 | G3PC2_MAIZE Glyceraldehyde_3_phosphate dehydrogenase 2_ cytosolic OS=Zea mays GN=GAPC2 PE=2 SV=1 | 10 | 0.550 | 0.098 | 0.000 | glycolysis |
| 536 | SP://O04294 | at4g02150 | IMPA3_ARatH Importin subunit alpha_3 OS=Arabidopsis thaliana GN=IMPA3 PE=1 SV=2 | 2 | 0.546 | 0.346 | 0.021 | protein |
| 537 | SP://Q42699 | at5g17920 | METE_CatRO 5_methyltetrahydropteroyltriglutamate__homocysteine methyltransferase OS=Catharanthus roseus GN=METE | 9 | 0.546 | 0.106 | 0.000 | amino acid metabolism |

| | | | | | | | | |
|---|---|---|---|---|---|---|---|---|
| 538 | SP://Q41607 | at3g43190 | SUS2_TULGE Sucrose synthase 2 OS=Tulipa gesneriana PE=2 SV=1 | 8 | 0.545 | 0.166 | 0.001 | major CHO metabolism |
| 539 | SP://Q7FAH2 | at1g13440 | G3PC2_ORYSJ Glyceraldehyde_3_phosphate dehydrogenase 2_ cytosolic OS=Oryza sativa subsp. japonica GN=GAPC2 | 7 | 0.543 | 0.106 | 0.000 | glycolysis |
| 540 | SP://Q41595 | at1g13440 | G3PC_TAXBA Glyceraldehyde_3_phosphate dehydrogenase_ cytosolic OS=Taxus baccata PE=2 SV=1 | 3 | 0.543 | 0.248 | 0.002 | glycolysis |
| 541 | SP://Q10MQ2 | at4g33680 | DAPat_ORYSJ Probable LL_diaminopimelate aminotransferase_ chloroplastic OS=Oryza sativa subsp. japonica GN=AGD2 | 4 | 0.542 | 0.251 | 0.025 | amino acid metabolism |
| 542 | SP://P50246 | at4g13940 | SAHH_MEDSA Adenosylhomocysteinase OS=Medicago sativa GN=SAHH PE=2 SV=1 | 12 | 0.541 | 0.158 | 0.002 | amino acid metabolism |
| 543 | SP://P32112 | at4g13940 | SAHH_WHEat Adenosylhomocysteinase OS=Triticum aestivum GN=SAHH PE=2 SV=1 | 22 | 0.541 | 0.102 | 0.000 | amino acid metabolism |
| 544 | SP://O04916 | at2g05710 | ACOC_SOLTU Aconitate hydratase_ cytoplasmic (Fragment) OS=Solanum tuberosum PE=2 SV=1 | 3 | 0.540 | 0.267 | 0.014 | TCA |
| 545 | SP://Q00917 | at5g49190 | SUS2_ARatH Sucrose synthase 2 OS=Arabidopsis thaliana GN=SUS2 PE=2 SV=3 | 2 | 0.540 | 0.280 | 0.032 | major CHO metabolism |
| 546 | SP://P34922 | at3g04120 | G3PC_PEA Glyceraldehyde_3_phosphate dehydrogenase_ cytosolic OS=Pisum sativum GN=GAPC1 PE=2 SV=1 | 5 | 0.540 | 0.116 | 0.000 | glycolysis |
| 547 | SP://B6DZD1 | at3g13790 | 1FEH_AEGSP Fructan 1_exohydrolase OS=Aegilops speltoides GN=1_FEH PE=3 SV=1 | 8 | 0.539 | 0.109 | 0.000 | major CHO metabolism |
| 548 | SP://Q39043 | at5g42020 | MD37F_ARatH Mediator of RNA polymerase II transcription subunit 37f OS=Arabidopsis thaliana GN=MED37F PE=1 SV=2 | 8 | 0.538 | 0.084 | 0.000 | stress |
| 549 | SP://Q43009 | at3g43190 | SUS3_ORYSJ Sucrose synthase 3 OS=Oryza sativa subsp. japonica GN=SUS3 PE=1 SV=2 | 4 | 0.538 | 0.216 | 0.003 | major CHO metabolism |
| 550 | SP://P49118 | at5g42020 | BIP_SOLLC Luminal_binding protein OS=Solanum lycopersicum PE=2 SV=1 | 7 | 0.538 | 0.078 | 0.000 | stress |
| 551 | SP://Q94A28 | at4g26970 | ACO3M_ARatH Aconitate hydratase 3_ mitochondrial OS=Arabidopsis thaliana GN=ACO3 PE=2 SV=3 | 2 | 0.538 | 0.224 | 0.010 | TCA |
| 552 | SP://O82663 | at5g66760 | SDHA1_ARatH Succinate dehydrogenase [ubiquinone] flavoprotein subunit 1_ mitochondrial OS=Arabidopsis thaliana | 9 | 0.538 | 0.134 | 0.000 | TCA |
| 553 | SP://Q39659 | at3g06860 | MFPA_CUCSA Glyoxysomal fatty acid beta_oxidation multifunctional protein MFP_a OS=Cucumis sativus PE=1 SV=1 | 2 | 0.538 | 0.174 | 0.003 | lipid metabolism |
| 554 | SP://Q0J8A4 | at3g04120 | G3PC1_ORYSJ Glyceraldehyde_3_phosphate dehydrogenase 1_ cytosolic OS=Oryza sativa subsp. japonica GN=GAPC1 | 6 | 0.537 | 0.112 | 0.000 | glycolysis |
| 555 | SP://P34921 | at1g13440 | G3PC_DIACA Glyceraldehyde_3_phosphate dehydrogenase_ cytosolic OS=Dianthus caryophyllus GN=GAPC PE=3 SV=1 | 8 | 0.537 | 0.095 | 0.000 | glycolysis |
| 556 | SP://B6DXP5 | at3g13790 | 1FEH_LEYCH Fructan 1_exohydrolase OS=Leymus chinensis GN=1_FEH PE=2 SV=1 | 6 | 0.536 | 0.162 | 0.000 | major CHO metabolism |
| 557 | SP://B6DZC8 | at3g13790 | 1FEH3_WHEat Fructan 1_exohydrolase w3 OS=Triticum aestivum GN=1_FEHw3 PE=1 SV=1 | 6 | 0.535 | 0.114 | 0.000 | major CHO metabolism |
| 558 | SP://P09094 | at3g04120 | G3PC_TOBAC Glyceraldehyde_3_phosphate dehydrogenase_ cytosolic (Fragment) OS=Nicotiana tabacum GN=GAPC | 3 | 0.535 | 0.122 | 0.000 | glycolysis |
| 559 | SP://Q70at7 | at3g13790 | 1FEH_HORVU Fructan 1_exohydrolase OS=Hordeum vulgare GN=1_FEH PE=2 SV=1 | 4 | 0.532 | 0.125 | 0.000 | major CHO metabolism |
| 560 | SP://O23255 | at4g13940 | SAHH1_ARatH Adenosylhomocysteinase 1 OS=Arabidopsis thaliana GN=SAHH1 PE=1 SV=1 | 13 | 0.531 | 0.166 | 0.007 | amino acid metabolism |
| 561 | SP://P49103 | at4g17170 | RAB2A_MAIZE Ras_related protein Rab_2_A OS=Zea mays GN=RAB2A PE=2 SV=1 | 7 | 0.531 | 0.142 | 0.000 | signalling |
| 562 | SP://P49104 | at4g17170 | RAB2B_MAIZE Ras_related protein Rab_2_B OS=Zea mays GN=RAB2B PE=2 SV=1 | 8 | 0.530 | 0.141 | 0.000 | signalling |
| 563 | SP://P31924 | at3g43190 | SUS1_ORYSJ Sucrose synthase 1 OS=Oryza sativa subsp. japonica GN=SUS1 PE=1 SV=1 | 6 | 0.530 | 0.201 | 0.006 | major CHO metabolism |
| 564 | SP://P49036 | at3g43190 | SUS2_MAIZE Sucrose synthase 2 OS=Zea mays GN=SUS1 PE=1 SV=1 | 6 | 0.530 | 0.201 | 0.006 | major CHO metabolism |
| 565 | SP://O24301 | at4g02280 | SUS2_PEA Sucrose synthase 2 OS=Pisum sativum GN=SUS2 PE=2 SV=1 | 2 | 0.529 | 0.270 | 0.008 | major CHO metabolism |
| 566 | SP://Q42652 | at4g02280 | SUSY_BETVU Sucrose synthase (Fragment) OS=Beta vulgaris GN=SS1 PE=2 SV=1 | 2 | 0.529 | 0.270 | 0.008 | major CHO metabolism |
| 567 | SP://Q9M111 | at4g02280 | SUS3_ARatH Sucrose synthase 3 OS=Arabidopsis thaliana GN=SUS3 PE=1 SV=1 | 2 | 0.529 | 0.270 | 0.008 | major CHO metabolism |
| 568 | SP://P04796 | at3g04120 | G3PC_SINAL Glyceraldehyde_3_phosphate dehydrogenase_ cytosolic OS=Sinapis alba GN=GAPC PE=2 SV=2 | 6 | 0.529 | 0.100 | 0.000 | glycolysis |
| 569 | SP://P31923 | at3g43190 | SUS2_HORVU Sucrose synthase 2 OS=Hordeum vulgare GN=SS2 PE=1 SV=1 | 5 | 0.528 | 0.203 | 0.001 | major CHO metabolism |
| 570 | SP://P68172 | at4g13940 | SAHH_NICSY Adenosylhomocysteinase OS=Nicotiana sylvestris GN=SAHH PE=2 SV=1 | 12 | 0.528 | 0.178 | 0.011 | amino acid metabolism |
| 571 | SP://P93253 | at4g13940 | SAHH_MESCR Adenosylhomocysteinase OS=Mesembryanthemum crystallinum GN=SAHH PE=2 SV=1 | 12 | 0.528 | 0.178 | 0.011 | amino acid metabolism |

| | | | | | | | | |
|---|---|---|---|---|---|---|---|---|
| 572 | SP://Q03684 | at5g42020 | BIP4_TOBAC Luminal_binding protein 4 OS=Nicotiana tabacum GN=BIP4 PE=2 SV=1 | 7 | 0.527 | 0.094 | 0.000 | stress |
| 573 | SP://Q03685 | at5g42020 | BIP5_TOBAC Luminal_binding protein 5 OS=Nicotiana tabacum GN=BIP5 PE=2 SV=1 | 7 | 0.527 | 0.094 | 0.000 | stress |
| 574 | SP://Q9LKR3 | at5g28540 | MD37A_ARatH Mediator of RNA polymerase II transcription subunit 37a OS=Arabidopsis thaliana GN=MED37A PE=1 SV=1 | 7 | 0.527 | 0.094 | 0.000 | stress |
| 575 | SP://Q01781 | at4g13940 | SAHH_PETCR Adenosylhomocysteinase OS=Petroselinum crispum GN=SAHH PE=2 SV=2 | 9 | 0.527 | 0.204 | 0.010 | amino acid metabolism |
| 576 | SP://B8BM17 | at3g52990 | KPYC2_ORYSI Pyruvate kinase 2_ cytosolic OS=Oryza sativa subsp. indica GN=OsI_37456 PE=3 SV=1 | 9 | 0.527 | 0.103 | 0.000 | glycolysis |
| 577 | SP://B8BJ39 | at3g52990 | KPYC1_ORYSI Pyruvate kinase 1_ cytosolic OS=Oryza sativa subsp. indica GN=OsI_35105 PE=3 SV=1 | 10 | 0.526 | 0.102 | 0.000 | glycolysis |
| 578 | SP://Q9SIB9 | at2g05710 | ACO2M_ARatH Aconitate hydratase 2_ mitochondrial OS=Arabidopsis thaliana GN=ACO2 PE=1 SV=2 | 8 | 0.526 | 0.117 | 0.000 | TCA |
| 579 | SP://Q42434 | at5g42020 | BIP_SPIOL Luminal_binding protein OS=Spinacia oleracea GN=HSC70 PE=2 SV=1 | 6 | 0.525 | 0.093 | 0.000 | stress |
| 580 | SP://Q6XZ79 | at2g31390 | SCRK1_MAIZE Fructokinase_1 OS=Zea mays GN=FRK1 PE=1 SV=1 | 4 | 0.525 | 0.171 | 0.002 | major CHO metabolism |
| 581 | SP://Q9LNE3 | at1g06030 | SCRK2_ARatH Probable fructokinase_2 OS=Arabidopsis thaliana GN=at1g06030 PE=2 SV=1 | 4 | 0.525 | 0.171 | 0.002 | major CHO metabolism |
| 582 | SP://Q9LNE4 | at1g06020 | SCRK3_ARatH Probable fructokinase_3 OS=Arabidopsis thaliana GN=at1g06020 PE=2 SV=1 | 4 | 0.525 | 0.171 | 0.002 | major CHO metabolism |
| 583 | SP://Q9SID0 | at2g31390 | SCRK1_ARatH Probable fructokinase_1 OS=Arabidopsis thaliana GN=at2g31390 PE=2 SV=1 | 4 | 0.525 | 0.171 | 0.002 | major CHO metabolism |
| 584 | SP://O50008 | at5g17920 | METE1_ARatH 5_methyltetrahydropteroyltriglutamate__homocysteine methyltransferase 1 OS=Arabidopsis thaliana | 9 | 0.523 | 0.079 | 0.000 | amino acid metabolism |
| 585 | SP://Q9SDX3 | at5g17310 | UGPA_MUSAC UTP__glucose_1_phosphate uridylyltransferase OS=Musa acuminata GN=UGPA PE=2 SV=1 | 7 | 0.523 | 0.165 | 0.001 | glycolysis |
| 586 | SP://Q43772 | at5g17310 | UGPA_HORVU UTP__glucose_1_phosphate uridylyltransferase OS=Hordeum vulgare PE=2 SV=1 | 17 | 0.522 | 0.109 | 0.000 | glycolysis |
| 587 | SP://Q43247 | at1g13440 | G3PC3_MAIZE Glyceraldehyde_3_phosphate dehydrogenase 3_ cytosolic OS=Zea mays GN=GAPC3 PE=2 SV=1 | 8 | 0.522 | 0.095 | 0.000 | glycolysis |
| 588 | SP://Q6YZX6 | at2g05710 | ACOC_ORYSJ Putative aconitate hydratase_ cytoplasmic OS=Oryza sativa subsp. japonica GN=Os08g0191100 PE=3 SV=1 | 12 | 0.521 | 0.083 | 0.000 | TCA |
| 589 | SP://Q9SRV5 | at3g03780 | METE2_ARatH 5_methyltetrahydropteroyltriglutamate__homocysteine methyltransferase 2 OS=Arabidopsis thaliana | 9 | 0.519 | 0.082 | 0.000 | amino acid metabolism |
| 590 | SP://O23888 | at3g43600 | ALDO2_MAIZE Indole_3_acetaldehyde oxidase OS=Zea mays GN=AO2 PE=2 SV=1 | 2 | 0.519 | 0.373 | 0.041 | hormone metabolism |
| 591 | SP://D2IGW7 | at1g55120 | 1FEH_BROPI Fructan 1_exohydrolase OS=Bromus pictus GN=1_FEHa PE=1 SV=1 | 2 | 0.518 | 0.211 | 0.004 | major CHO metabolism |
| 592 | SP://P08477 | at3g04120 | G3PC2_HORVU Glyceraldehyde_3_phosphate dehydrogenase 2_ cytosolic (Fragment) OS=Hordeum vulgare GN=GAPC | 11 | 0.518 | 0.092 | 0.000 | glycolysis |
| 593 | SP://P50249 | at4g13940 | SAHH_PHASS Adenosylhomocysteinase OS=Phalaenopsis sp. GN=SAHH PE=2 SV=1 | 13 | 0.518 | 0.170 | 0.008 | amino acid metabolism |
| 594 | SP://P04712 | at3g43190 | SUS1_MAIZE Sucrose synthase 1 OS=Zea mays GN=SH_1 PE=2 SV=1 | 16 | 0.517 | 0.105 | 0.000 | major CHO metabolism |
| 595 | SP://P28408 | atCg00490 | RBL_DROLU Ribulose bisphosphate carboxylase large chain (Fragment) OS=Drosophyllum lusitanicum GN=rbcL PE=3 SV=2 | 3 | 0.517 | 0.249 | 0.007 | PS |
| 596 | SP://P28453 | atCg00490 | RBL_SCUBO Ribulose bisphosphate carboxylase large chain (Fragment) OS=Scutellaria bolanderi GN=rbcL PE=3 SV=1 | 3 | 0.517 | 0.249 | 0.007 | PS |
| 597 | SP://O64459 | at5g17310 | UGPA_PYRPY UTP__glucose_1_phosphate uridylyltransferase OS=Pyrus pyrifolia PE=2 SV=1 | 5 | 0.517 | 0.233 | 0.045 | glycolysis |
| 598 | SP://A2Z7B3 | at5g28840 | GME1_ORYSI GDP_mannose 3_5_epimerase 1 OS=Oryza sativa subsp. indica GN=OsI_032456 PE=2 SV=1 | 3 | 0.515 | 0.239 | 0.036 | redox |
| 599 | SP://A3C4S4 | at5g28840 | GME1_ORYSJ GDP_mannose 3_5_epimerase 1 OS=Oryza sativa subsp. japonica GN=GME_1 PE=1 SV=1 | 3 | 0.515 | 0.239 | 0.036 | redox |
| 600 | SP://Q6K5G8 | at3g04120 | G3PC3_ORYSJ Glyceraldehyde_3_phosphate dehydrogenase 3_ cytosolic OS=Oryza sativa subsp. japonica GN=GAPC3 | 7 | 0.515 | 0.099 | 0.000 | glycolysis |
| 601 | SP://P30298 | at3g43190 | SUS2_ORYSJ Sucrose synthase 2 OS=Oryza sativa subsp. japonica GN=SUS2 PE=1 SV=2 | 19 | 0.515 | 0.095 | 0.000 | major CHO metabolism |
| 602 | SP://P49210 | at1g33140 | RL9_ORYSJ 60S ribosomal protein L9 OS=Oryza sativa subsp. japonica GN=RPL9 PE=2 SV=3 | 2 | 0.515 | 0.393 | 0.009 | protein |
| 603 | SP://Q852M1 | at3g43600 | ALDO2_ORYSJ Probable aldehyde oxidase 2 OS=Oryza sativa subsp. japonica GN=Os03g0790900 PE=2 SV=1 | 3 | 0.514 | 0.339 | 0.032 | hormone metabolism |
| 604 | SP://Q852M2 | at3g43600 | ALDO3_ORYSJ Probable aldehyde oxidase 3 OS=Oryza sativa subsp. japonica GN=Os03g0790700 PE=3 SV=1 | 3 | 0.514 | 0.339 | 0.032 | hormone metabolism |
| 605 | SP://Q9LK36 | at3g23810 | SAHH2_ARatH Adenosylhomocysteinase 2 OS=Arabidopsis thaliana GN=SAHH2 PE=1 SV=1 | 11 | 0.514 | 0.209 | 0.028 | amino acid metabolism |

| | | | | | | | | |
|---|---|---|---|---|---|---|---|---|
| 606 | SP://Q9CAA0 | at1g79990 | COB21_ARatH Coatomer subunit beta__1 OS=Arabidopsis thaliana GN=at1g79990 PE=2 SV=2 | 2 | 0.514 | 0.316 | 0.026 | cell.vesicle transport |
| 607 | SP://P25861 | at3g04120 | G3PC_ANTMA Glyceraldehyde_3_phosphate dehydrogenase_ cytosolic OS=Antirrhinum majus GN=GAPC PE=2 SV=1 | 7 | 0.513 | 0.098 | 0.000 | glycolysis |
| 608 | SP://Q9LKG7 | at5g17310 | UGPA_ASTPN UTP__glucose_1_phosphate uridylyltransferase OS=Astragalus penduliflorus GN=UGP PE=2 SV=1 | 7 | 0.512 | 0.167 | 0.000 | glycolysis |
| 609 | SP://P57751 | at5g17310 | UGPA1_ARatH UTP__glucose_1_phosphate uridylyltransferase 1 OS=Arabidopsis thaliana GN=at5g17310 PE=2 SV=1 | 6 | 0.512 | 0.172 | 0.001 | glycolysis |
| 610 | SP://Q9M9P3 | at3g03250 | UGPA2_ARatH Probable UTP__glucose_1_phosphate uridylyltransferase 2 OS=Arabidopsis thaliana GN=at3g03250 PE=1 SV=1 | 6 | 0.512 | 0.172 | 0.001 | glycolysis |
| 611 | SP://P49608 | at2g05710 | ACOC_CUCMA Aconitate hydratase_ cytoplasmic OS=Cucurbita maxima PE=2 SV=1 | 9 | 0.512 | 0.125 | 0.001 | TCA |
| 612 | SP://Q41608 | at3g43190 | SUS1_TULGE Sucrose synthase 1 OS=Tulipa gesneriana PE=2 SV=1 | 6 | 0.511 | 0.213 | 0.004 | major CHO metabolism |
| 613 | SP://Q6ESI7 | at4g20850 | TPPII_ORYSJ Tripeptidyl_peptidase 2 OS=Oryza sativa subsp. japonica GN=TPP2 PE=2 SV=1 | 7 | 0.508 | 0.159 | 0.007 | protein |
| 614 | SP://P35007 | at3g23810 | SAHH_CatRO Adenosylhomocysteinase OS=Catharanthus roseus GN=SAHH PE=2 SV=1 | 10 | 0.507 | 0.204 | 0.009 | amino acid metabolism |
| 615 | SP://P08436 | at5g59970 | H4_VOLCA Histone H4 OS=Volvox carteri GN=H4_I PE=3 SV=2 | 2 | 0.504 | 0.373 | 0.039 | cell.organisation |
| 616 | SP://P50566 | at5g59970 | H4_CHLRE Histone H4 OS=Chlamydomonas reinhardtii GN=H4_I PE=3 SV=2 | 2 | 0.504 | 0.373 | 0.039 | cell.organisation |
| 617 | SP://P62785 | at5g59970 | H41_WHEat Histone H4 variant TH011 OS=Triticum aestivum PE=3 SV=2 | 2 | 0.504 | 0.373 | 0.039 | cell.organisation |
| 618 | SP://P62786 | at5g59970 | H42_WHEat Histone H4 variant TH091 OS=Triticum aestivum PE=3 SV=2 | 2 | 0.504 | 0.373 | 0.039 | cell.organisation |
| 619 | SP://Q41811 | at5g59970 | H43_MAIZE Histone H4.3 OS=Zea mays GN=H4 PE=1 SV=3 | 2 | 0.504 | 0.373 | 0.039 | cell.organisation |
| 620 | SP://Q71V09 | at5g59970 | H4_CAPAN Histone H4 OS=Capsicum annuum PE=3 SV=3 | 2 | 0.504 | 0.373 | 0.039 | cell.organisation |
| 621 | SP://Q42560 | at4g35830 | ACO1_ARatH Aconitate hydratase 1 OS=Arabidopsis thaliana GN=ACO1 PE=1 SV=2 | 9 | 0.504 | 0.107 | 0.000 | TCA |
| 622 | SP://P38546 | at5g55190 | RAN1_SOLLC GTP_binding nuclear protein Ran1 OS=Solanum lycopersicum GN=RAN1 PE=2 SV=1 | 3 | 0.502 | 0.466 | 0.023 | signalling |
| 623 | SP://P38547 | at5g55190 | RAN2_SOLLC GTP_binding nuclear protein Ran2 OS=Solanum lycopersicum GN=RAN2 PE=2 SV=1 | 3 | 0.502 | 0.466 | 0.023 | signalling |
| 624 | SP://P38548 | at5g55190 | RAN_VICFA GTP_binding nuclear protein Ran/TC4 OS=Vicia faba PE=2 SV=1 | 3 | 0.502 | 0.466 | 0.023 | signalling |
| 625 | SP://P41916 | at5g20010 | RAN1_ARatH GTP_binding nuclear protein Ran_1 OS=Arabidopsis thaliana GN=RAN1 PE=1 SV=1 | 3 | 0.502 | 0.466 | 0.023 | signalling |
| 626 | SP://P41917 | at5g20020 | RAN2_ARatH GTP_binding nuclear protein Ran_2 OS=Arabidopsis thaliana GN=RAN2 PE=1 SV=3 | 3 | 0.502 | 0.466 | 0.023 | signalling |
| 627 | SP://P41918 | at5g55190 | RANA1_TOBAC GTP_binding nuclear protein Ran_A1 OS=Nicotiana tabacum GN=RAN_A1 PE=2 SV=1 | 3 | 0.502 | 0.466 | 0.023 | signalling |
| 628 | SP://P41919 | at5g55190 | RANB1_TOBAC GTP_binding nuclear protein Ran_B1 OS=Nicotiana tabacum GN=RAN_B1 PE=2 SV=1 | 3 | 0.502 | 0.466 | 0.023 | signalling |
| 629 | SP://P54765 | at5g55190 | RAN1A_LOTJA GTP_binding nuclear protein Ran1A (Fragment) OS=Lotus japonicus GN=RAN1A PE=2 SV=1 | 3 | 0.502 | 0.466 | 0.023 | signalling |
| 630 | SP://P54766 | at5g55190 | RAN1B_LOTJA GTP_binding nuclear protein Ran1B (Fragment) OS=Lotus japonicus GN=RAN1B PE=2 SV=1 | 3 | 0.502 | 0.466 | 0.023 | signalling |
| 631 | SP://Q8H156 | at5g55190 | RAN3_ARatH GTP_binding nuclear protein Ran_3 OS=Arabidopsis thaliana GN=RAN3 PE=1 SV=2 | 3 | 0.502 | 0.466 | 0.023 | signalling |
| 632 | SP://B6DZD2 | at3g13790 | 1FEH_AEGTA Fructan 1_exohydrolase OS=Aegilops tauschii GN=1_FEH PE=3 SV=1 | 8 | 0.502 | 0.099 | 0.000 | major CHO metabolism |
| 633 | SP://Q84LA1 | at3g13790 | 1FEH2_WHEat Fructan 1_exohydrolase w2 OS=Triticum aestivum GN=1_FEHw2 PE=1 SV=1 | 8 | 0.502 | 0.099 | 0.000 | major CHO metabolism |
| 634 | SP://O49513 | at4g18430 | RAA1E_ARatH Ras_related protein RABA1e OS=Arabidopsis thaliana GN=RABA1E PE=2 SV=1 | 3 | 0.502 | 0.228 | 0.010 | signalling |
| 635 | SP://Q39434 | at1g07410 | RB2BV_BETVU Ras_related protein Rab2BV OS=Beta vulgaris GN=RAB2BV PE=2 SV=1 | 3 | 0.502 | 0.228 | 0.010 | signalling |
| 636 | SP://Q40193 | at1g07410 | RB11C_LOTJA Ras_related protein Rab11C OS=Lotus japonicus GN=RAB11C PE=2 SV=1 | 3 | 0.502 | 0.228 | 0.010 | signalling |
| 637 | SP://Q40521 | at5g60860 | RB11B_TOBAC Ras_related protein Rab11B OS=Nicotiana tabacum GN=RAB11B PE=2 SV=1 | 3 | 0.502 | 0.228 | 0.010 | signalling |
| 638 | SP://Q96283 | at3g46830 | RAA2C_ARatH Ras_related protein RABA2c OS=Arabidopsis thaliana GN=RABA2C PE=2 SV=4 | 3 | 0.502 | 0.228 | 0.010 | signalling |
| 639 | SP://Q9FIF9 | at5g59150 | RAA2D_ARatH Ras_related protein RABA2d OS=Arabidopsis thaliana GN=RABA2D PE=2 SV=1 | 3 | 0.502 | 0.228 | 0.010 | signalling |

| | | | | | | | | |
|---|---|---|---|---|---|---|---|---|
| 640 | SP://Q9LNW1 | at1g07410 | RAA2B_ARatH Ras_related protein RABA2b OS=Arabidopsis thaliana GN=RABA2B PE=2 SV=2 | 3 | 0.502 | 0.228 | 0.010 | signalling |
| 641 | SP://B6DZD0 | at3g13790 | 1FEH_TRIUA Fructan 1_exohydrolase OS=Triticum urartu GN=1_FEH PE=3 SV=1 | 9 | 0.502 | 0.098 | 0.000 | major CHO metabolism |
| 642 | SP://Q84PN8 | at3g13790 | 1FEH1_WHEat Fructan 1_exohydrolase w1 OS=Triticum aestivum GN=1_FEHw1 PE=1 SV=1 | 9 | 0.502 | 0.098 | 0.000 | major CHO metabolism |
| 643 | SP://O23657 | at1g43890 | RABC1_ARatH Ras_related protein RABC1 OS=Arabidopsis thaliana GN=RABC1 PE=1 SV=1 | 3 | 0.501 | 0.274 | 0.020 | signalling |
| 644 | SP://P49034 | at3g43190 | SUSY_ALNGL Sucrose synthase OS=Alnus glutinosa GN=SUS1 PE=2 SV=1 | 3 | 0.500 | 0.323 | 0.013 | major CHO metabolism |
| 645 | SP://Q9FWY7 | at1g02690 | IMPA6_ARatH Importin subunit alpha_6 OS=Arabidopsis thaliana GN=IMPA6 PE=2 SV=1 | 3 | 0.499 | 0.283 | 0.009 | protein |
| 646 | SP://Q9SA73 | at1g30580 | OLA1_ARatH Obg_like atPase 1 OS=Arabidopsis thaliana GN=YchF1 PE=1 SV=1 | 2 | 0.499 | 0.329 | 0.003 | not assigned |
| 647 | SP://Q43207 | at3g25230 | FKB70_WHEat 70 kDa peptidyl_prolyl isomerase OS=Triticum aestivum GN=FKBP70 PE=1 SV=1 | 4 | 0.497 | 0.314 | 0.012 | protein |
| 648 | SP://Q2R1V8 | at5g28840 | GME2_ORYSJ GDP_mannose 3_5_epimerase 2 OS=Oryza sativa subsp. japonica GN=GME_2 PE=2 SV=2 | 2 | 0.489 | 0.293 | 0.046 | redox |
| 649 | SP://Q93VR3 | at5g28840 | GME_ARatH GDP_mannose 3_5_epimerase OS=Arabidopsis thaliana GN=at5g28840 PE=1 SV=1 | 2 | 0.489 | 0.293 | 0.046 | redox |
| 650 | SP://A2WSI7 | at5g55190 | RAN1_ORYSI GTP_binding nuclear protein Ran_1 OS=Oryza sativa subsp. indica GN=RAN1 PE=2 SV=2 | 2 | 0.488 | 0.583 | 0.041 | signalling |
| 651 | SP://Q42971 | at2g36530 | ENO_ORYSJ Enolase OS=Oryza sativa subsp. japonica GN=ENO1 PE=1 SV=2 | 18 | 0.487 | 0.088 | 0.000 | glycolysis |
| 652 | SP://P17878 | at1g13440 | G3PC_MESCR Glyceraldehyde_3_phosphate dehydrogenase_ cytosolic OS=Mesembryanthemum crystallinum GN=GAPC | 4 | 0.486 | 0.160 | 0.000 | glycolysis |
| 653 | SP://P37829 | at3g59480 | SCRK_SOLTU Fructokinase OS=Solanum tuberosum PE=2 SV=1 | 2 | 0.485 | 0.177 | 0.000 | major CHO metabolism |
| 654 | SP://Q42896 | at3g59480 | SCRK2_SOLLC Fructokinase_2 OS=Solanum lycopersicum GN=FRK2 PE=2 SV=2 | 2 | 0.485 | 0.177 | 0.000 | major CHO metabolism |
| 655 | SP://Q7XJ81 | at3g59480 | SCRK2_SOLHA Fructokinase_2 OS=Solanum habrochaites GN=FRK2 PE=2 SV=1 | 2 | 0.485 | 0.177 | 0.000 | major CHO metabolism |
| 656 | SP://Q9M1B9 | at3g59480 | SCRK4_ARatH Probable fructokinase_4 OS=Arabidopsis thaliana GN=at3g59480 PE=2 SV=1 | 2 | 0.485 | 0.177 | 0.000 | major CHO metabolism |
| 657 | SP://P85085 | atCg00490 | RBL_VITSX Ribulose bisphosphate carboxylase large chain (Fragments) OS=Vitis sp. GN=rbcL PE=1 SV=1 | 2 | 0.485 | 0.388 | 0.011 | PS |
| 658 | SP://Q9SF16 | at3g11830 | TCPH_ARatH T_complex protein 1 subunit eta OS=Arabidopsis thaliana GN=CCT7 PE=1 SV=1 | 4 | 0.484 | 0.200 | 0.002 | protein |
| 659 | SP://P26300 | at2g36530 | ENO_SOLLC Enolase OS=Solanum lycopersicum GN=PGH1 PE=2 SV=1 | 7 | 0.482 | 0.093 | 0.000 | glycolysis |
| 660 | SP://P25696 | at2g36530 | ENO2_ARatH Bifunctional enolase 2/transcriptional activator OS=Arabidopsis thaliana GN=ENO2 PE=1 SV=1 | 8 | 0.481 | 0.089 | 0.000 | glycolysis |
| 661 | SP://P42895 | at2g36530 | ENO2_MAIZE Enolase 2 OS=Zea mays GN=ENO2 PE=2 SV=1 | 16 | 0.479 | 0.069 | 0.000 | glycolysis |
| 662 | SP://Q6XJG8 | at4g28470 | PSD2B_ARatH 26S proteasome non_atPase regulatory subunit 2 homolog B OS=Arabidopsis thaliana GN=RPN1B PE=1 SV=1 | 4 | 0.475 | 0.272 | 0.000 | protein |
| 663 | SP://Q43321 | at2g36530 | ENO_ALNGL Enolase OS=Alnus glutinosa GN=PGH1 PE=2 SV=1 | 6 | 0.474 | 0.101 | 0.000 | glycolysis |
| 664 | SP://Q9LEI9 | at2g36530 | ENO2_HEVBR Enolase 2 OS=Hevea brasiliensis GN=ENO2 PE=1 SV=1 | 6 | 0.473 | 0.107 | 0.000 | glycolysis |
| 665 | SP://P52184 | at2g19770 | PROF1_HORVU Profilin_1 OS=Hordeum vulgare GN=PRO1 PE=2 SV=1 | 3 | 0.473 | 0.357 | 0.003 | cell.organisation |
| 666 | SP://Q42669 | at4g35830 | ACOC_CUCMC Aconitate hydratase (Fragment) OS=Cucumis melo var. conomon GN=ACO PE=2 SV=1 | 4 | 0.473 | 0.184 | 0.012 | TCA |
| 667 | SP://Q0JGZ6 | at2g31390 | SCRK1_ORYSJ Fructokinase_1 OS=Oryza sativa subsp. japonica GN=FRK1 PE=1 SV=2 | 5 | 0.473 | 0.136 | 0.000 | major CHO metabolism |
| 668 | SP://Q9LEJ0 | at2g36530 | ENO1_HEVBR Enolase 1 OS=Hevea brasiliensis GN=ENO1 PE=1 SV=1 | 6 | 0.472 | 0.112 | 0.000 | glycolysis |
| 669 | SP://Q7GD79 | at5g55190 | RAN2_ORYSJ GTP_binding nuclear protein Ran_2 OS=Oryza sativa subsp. japonica GN=RAN2 PE=2 SV=1 | 4 | 0.471 | 0.403 | 0.013 | signalling |
| 670 | SP://P25777 | at5g43060 | ORYB_ORYSJ Oryzain beta chain OS=Oryza sativa subsp. japonica GN=Os04g0670200 PE=1 SV=2 | 3 | 0.469 | 0.196 | 0.020 | protein |
| 671 | SP://P34742 | at4g16260 | E13A_HORVU Glucan endo_1_3_beta_glucosidase GI OS=Hordeum vulgare PE=1 SV=2 | 2 | 0.469 | 0.376 | 0.020 | misc |
| 672 | SP://Q43130 | at2g36530 | ENO_MESCR Enolase OS=Mesembryanthemum crystallinum GN=PGH1 PE=2 SV=1 | 7 | 0.467 | 0.100 | 0.000 | glycolysis |
| 673 | SP://Q9SIV2 | at2g20580 | PSD2A_ARatH 26S proteasome non_atPase regulatory subunit 2 homolog A OS=Arabidopsis thaliana GN=RPN1A PE=1 SV=2 | 5 | 0.463 | 0.257 | 0.000 | protein |

| | | | | | | | | |
|---|---|---|---|---|---|---|---|---|
| 674 | SP://Q8LF48 | at1g04710 | THIK1_ARatH 3_ketoacyl_CoA thiolase 1_ peroxisomal OS=Arabidopsis thaliana GN=Kat1 PE=2 SV=2 | 2 | 0.463 | 0.230 | 0.000 | amino acid metabolism |
| 675 | SP://P26301 | at2g36530 | ENO1_MAIZE Enolase 1 OS=Zea mays GN=ENO1 PE=2 SV=1 | 11 | 0.462 | 0.096 | 0.000 | glycolysis |
| 676 | SP://Q7F4F8 | at3g01280 | VDAC3_ORYSJ Mitochondrial outer membrane protein porin 3 OS=Oryza sativa subsp. japonica GN=VDAC3 PE=2 SV=1 | 2 | 0.461 | 0.413 | 0.025 | transport |
| 677 | SP://A2WXV8 | at2g31390 | SCRK1_ORYSI Fructokinase_1 OS=Oryza sativa subsp. indica GN=FRK1 PE=1 SV=1 | 4 | 0.461 | 0.154 | 0.000 | major CHO metabolism |
| 678 | SP://Q2R480 | at5g41670 | 6PGD2_ORYSJ 6_phosphogluconate dehydrogenase_ decarboxylating 2_ chloroplastic OS=Oryza sativa subsp. japonica | 3 | 0.458 | 0.321 | 0.015 | OPP |
| 679 | SP://Q9LI00 | at3g02360 | 6PGD1_ORYSJ 6_phosphogluconate dehydrogenase_ decarboxylating 1 OS=Oryza sativa subsp. japonica GN=G6PGH1 | 8 | 0.458 | 0.115 | 0.000 | OPP |
| 680 | SP://P49232 | at4g29350 | PROF1_WHEat Profilin_1 OS=Triticum aestivum GN=PRO1 PE=2 SV=2 | 4 | 0.452 | 0.353 | 0.005 | cell.organisation |
| 681 | SP://P49233 | at2g19770 | PROF2_WHEat Profilin_2 OS=Triticum aestivum GN=PRO2 PE=2 SV=2 | 4 | 0.452 | 0.353 | 0.005 | cell.organisation |
| 682 | SP://Q0WNZ5 | at5g20980 | METE3_ARatH 5_methyltetrahydropteroyltriglutamate__homocysteine methyltransferase 3_ chloroplastic OS=Arabidopsis thaliana | 5 | 0.448 | 0.151 | 0.001 | amino acid metabolism |
| 683 | SP://P49234 | at2g19770 | PROF3_WHEat Profilin_3 OS=Triticum aestivum GN=PRO3 PE=2 SV=2 | 3 | 0.440 | 0.406 | 0.013 | cell.organisation |
| 684 | SP://Q56WD9 | at2g33150 | THIK2_ARatH 3_ketoacyl_CoA thiolase 2_ peroxisomal OS=Arabidopsis thaliana GN=PED1 PE=1 SV=2 | 2 | 0.439 | 0.297 | 0.004 | amino acid metabolism |
| 685 | SP://Q7G192 | at3g43600 | ALDO2_ARatH Indole_3_acetaldehyde oxidase OS=Arabidopsis thaliana GN=AAO2 PE=1 SV=2 | 2 | 0.439 | 0.502 | 0.041 | hormone metabolism |
| 686 | SP://Q9FFR3 | at5g41670 | 6PGD2_ARatH 6_phosphogluconate dehydrogenase_ decarboxylating 2_ chloroplastic OS=Arabidopsis thaliana GN=at5g41670 | 2 | 0.436 | 0.312 | 0.003 | OPP |
| 687 | SP://Q9SH69 | at1g64190 | 6PGD1_ARatH 6_phosphogluconate dehydrogenase_ decarboxylating 1_ chloroplastic OS=Arabidopsis thaliana GN=at1g64190 | 2 | 0.436 | 0.312 | 0.003 | OPP |
| 688 | SP://Q94KU2 | at5g41670 | 6PGD2_SPIOL 6_phosphogluconate dehydrogenase_ decarboxylating 2_ chloroplastic OS=Spinacia oleracea GN=pgdP | 3 | 0.436 | 0.292 | 0.004 | OPP |
| 689 | SP://Q9SJH7 | at2g42790 | CISY3_ARatH Citrate synthase 3_ peroxisomal OS=Arabidopsis thaliana GN=CSY3 PE=2 SV=1 | 3 | 0.433 | 0.276 | 0.005 | gluconeogenese |
| 690 | SP://P34899 | at4g37930 | GLYM_PEA Serine hydroxymethyltransferase_ mitochondrial OS=Pisum sativum PE=1 SV=1 | 2 | 0.433 | 0.359 | 0.016 | C1-metabolism |
| 691 | SP://Q94KU1 | at3g02360 | 6PGD1_SPIOL 6_phosphogluconate dehydrogenase_ decarboxylating 1 OS=Spinacia oleracea GN=pgdC PE=1 SV=1 | 4 | 0.428 | 0.255 | 0.002 | OPP |
| 692 | SP://O22607 | at2g19520 | MSI4_ARatH WD_40 repeat_containing protein MSI4 OS=Arabidopsis thaliana GN=MSI4 PE=1 SV=3 | 4 | 0.427 | 0.266 | 0.003 | development |
| 693 | SP://Q96321 | at3g06720 | IMPA1_ARatH Importin subunit alpha_1 OS=Arabidopsis thaliana GN=IMPA1 PE=1 SV=2 | 2 | 0.426 | 0.412 | 0.037 | protein |
| 694 | SP://O65735 | at3g52930 | ALF_CICAR Fructose_bisphosphate aldolase_ cytoplasmic isozyme OS=Cicer arietinum GN=ALDC PE=2 SV=1 | 3 | 0.419 | 0.401 | 0.006 | glycolysis |
| 695 | SP://P46257 | at3g52930 | ALF2_PEA Fructose_bisphosphate aldolase_ cytoplasmic isozyme 2 OS=Pisum sativum PE=3 SV=1 | 3 | 0.419 | 0.401 | 0.006 | glycolysis |
| 696 | SP://Q9XI98 | at1g49300 | RAG3E_ARatH Ras_related protein RABG3e OS=Arabidopsis thaliana GN=RABG3E PE=2 SV=1 | 4 | 0.418 | 0.265 | 0.009 | signalling |
| 697 | SP://Q9SZX3 | at4g24830 | ASSY_ARatH Argininosuccinate synthase_ chloroplastic OS=Arabidopsis thaliana GN=at4g24830 PE=1 SV=3 | 2 | 0.417 | 0.138 | 0.000 | amino acid metabolism |
| 698 | SP://Q9FWA3 | at3g02360 | 6GPD3_ARatH 6_phosphogluconate dehydrogenase_ decarboxylating 3 OS=Arabidopsis thaliana GN=at3g02360 PE=2 SV=1 | 5 | 0.415 | 0.246 | 0.004 | OPP |
| 699 | SP://Q07346 | at5g17330 | DCE_PETHY Glutamate decarboxylase OS=Petunia hybrida GN=GAD PE=1 SV=1 | 4 | 0.414 | 0.232 | 0.022 | amino acid metabolism |
| 700 | SP://P50218 | at1g65930 | IDHC_TOBAC Isocitrate dehydrogenase [NADP] OS=Nicotiana tabacum PE=2 SV=1 | 4 | 0.413 | 0.208 | 0.002 | TCA |
| 701 | SP://O24562 | at3g19450 | CADH_MAIZE Probable cinnamyl alcohol dehydrogenase OS=Zea mays GN=CAD PE=2 SV=1 | 3 | 0.411 | 0.284 | 0.042 | secondary metabolism |
| 702 | SP://O82056 | at3g19450 | CADH_SACOF Probable cinnamyl alcohol dehydrogenase OS=Saccharum officinarum GN=CAD PE=2 SV=1 | 2 | 0.411 | 0.302 | 0.023 | secondary metabolism |
| 703 | SP://Q8H103 | at4g24620 | G6PIP_ARatH Glucose_6_phosphate isomerase 1_ chloroplastic OS=Arabidopsis thaliana GN=PGI1 PE=1 SV=1 | 5 | 0.410 | 0.223 | 0.000 | glycolysis |
| 704 | SP://Q42521 | at5g17330 | DCE1_ARatH Glutamate decarboxylase 1 OS=Arabidopsis thaliana GN=GAD1 PE=1 SV=2 | 4 | 0.410 | 0.229 | 0.007 | amino acid metabolism |
| 705 | SP://Q9ZPS3 | at2g02010 | DCE4_ARatH Glutamate decarboxylase 4 OS=Arabidopsis thaliana GN=GAD4 PE=1 SV=1 | 4 | 0.410 | 0.229 | 0.007 | amino acid metabolism |
| 706 | SP://P50217 | at1g65930 | IDHC_SOLTU Isocitrate dehydrogenase [NADP] OS=Solanum tuberosum GN=ICDH_1 PE=2 SV=1 | 5 | 0.409 | 0.205 | 0.003 | TCA |

| | | | | | | | | |
|---|---|---|---|---|---|---|---|---|
| 707 | SP://Q9ZPS4 | at2g02000 | DCE3_ARatH Glutamate decarboxylase 3 OS=Arabidopsis thaliana GN=GAD3 PE=2 SV=1 | 2 | 0.408 | 0.234 | 0.002 | amino acid metabolism |
| 708 | SP://P28185 | at1g06400 | RAA1A_ARatH Ras_related protein RABA1a OS=Arabidopsis thaliana GN=RABA1A PE=1 SV=1 | 2 | 0.408 | 0.314 | 0.036 | signalling |
| 709 | SP://Q01111 | at1g06400 | YPT3_NICPL Ras_related protein YPT3 OS=Nicotiana plumbaginifolia GN=YPT3 PE=2 SV=1 | 2 | 0.408 | 0.314 | 0.036 | signalling |
| 710 | SP://Q40195 | at1g06400 | RB11E_LOTJA Ras_related protein Rab11E OS=Lotus japonicus GN=RAB11E PE=2 SV=1 | 2 | 0.408 | 0.314 | 0.036 | signalling |
| 711 | SP://P0DH99 | at5g60390 | EF1A1_ARatH Elongation factor 1_alpha 1 OS=Arabidopsis thaliana GN=A1 PE=1 SV=1 | 10 | 0.406 | 0.140 | 0.000 | protein |
| 712 | SP://Q9SLK0 | at1g54340 | ICDHX_ARatH Peroxisomal isocitrate dehydrogenase [NADP] OS=Arabidopsis thaliana GN=ICDH PE=1 SV=1 | 4 | 0.405 | 0.151 | 0.001 | TCA |
| 713 | SP://O23627 | at1g29880 | SYGM1_ARatH Glycine__tRNA ligase_ mitochondrial 1 OS=Arabidopsis thaliana GN=at1g29880 PE=1 SV=1 | 3 | 0.405 | 0.205 | 0.012 | protein |
| 714 | SP://Q06197 | at1g65930 | IDHC_SOYBN Isocitrate dehydrogenase [NADP] OS=Glycine max GN=IDH1 PE=2 SV=2 | 6 | 0.405 | 0.203 | 0.006 | TCA |
| 715 | SP://Q9SRZ6 | at1g65930 | ICDHC_ARatH Cytosolic isocitrate dehydrogenase [NADP] OS=Arabidopsis thaliana GN=CICDH PE=2 SV=1 | 6 | 0.405 | 0.203 | 0.006 | TCA |
| 716 | SP://P29521 | at5g60390 | EF1A1_DAUCA Elongation factor 1_alpha OS=Daucus carota PE=1 SV=1 | 9 | 0.405 | 0.142 | 0.000 | protein |
| 717 | SP://Q40194 | at1g06400 | RB11D_LOTJA Ras_related protein Rab11D OS=Lotus japonicus GN=RAB11D PE=2 SV=1 | 3 | 0.403 | 0.299 | 0.014 | signalling |
| 718 | SP://Q03033 | at5g60390 | EF1A_WHEat Elongation factor 1_alpha OS=Triticum aestivum GN=TEF1 PE=2 SV=1 | 14 | 0.402 | 0.117 | 0.000 | protein |
| 719 | SP://P42862 | at5g42740 | G6PIA_ORYSJ Glucose_6_phosphate isomerase_ cytosolic A OS=Oryza sativa subsp. japonica GN=Os03g0776000 PE=2 SV=2 | 3 | 0.401 | 0.182 | 0.000 | glycolysis |
| 720 | SP://P42863 | at5g42740 | G6PIB_ORYSJ Glucose_6_phosphate isomerase_ cytosolic B OS=Oryza sativa subsp. japonica GN=Os06g0256500 PE=2 SV=2 | 3 | 0.401 | 0.182 | 0.000 | glycolysis |
| 721 | SP://P49105 | at5g42740 | G6PI_MAIZE Glucose_6_phosphate isomerase_ cytosolic OS=Zea mays GN=PHI1 PE=2 SV=1 | 4 | 0.400 | 0.181 | 0.000 | glycolysis |
| 722 | SP://Q40345 | at1g65930 | IDHP_MEDSA Isocitrate dehydrogenase [NADP]_ chloroplastic (Fragment) OS=Medicago sativa PE=2 SV=1 | 7 | 0.398 | 0.134 | 0.000 | TCA |
| 723 | SP://Q6K9N6 | at2g20420 | SUCB_ORYSJ Succinyl_CoA ligase [ADP_forming] subunit beta_ mitochondrial OS=Oryza sativa subsp. japonica | 6 | 0.395 | 0.106 | 0.000 | TCA |
| 724 | SP://P28011 | at5g11520 | Aat1_MEDSA Aspartate aminotransferase 1 OS=Medicago sativa GN=Aat_1 PE=2 SV=2 | 2 | 0.389 | 0.175 | 0.001 | amino acid metabolism |
| 725 | SP://P31022 | at3g18820 | RAB7_PEA Ras_related protein Rab7 OS=Pisum sativum PE=2 SV=1 | 5 | 0.388 | 0.224 | 0.004 | signalling |
| 726 | SP://Q40787 | at3g18820 | RAB7_CENCI Ras_related protein Rab7 OS=Cenchrus ciliaris PE=2 SV=1 | 5 | 0.388 | 0.224 | 0.004 | signalling |
| 727 | SP://Q9LS94 | at3g18820 | RAG3F_ARatH Ras_related protein RABG3f OS=Arabidopsis thaliana GN=RABG3F PE=2 SV=1 | 5 | 0.388 | 0.224 | 0.004 | signalling |
| 728 | SP://P08440 | at2g36460 | ALF_MAIZE Fructose_bisphosphate aldolase_ cytoplasmic isozyme OS=Zea mays PE=2 SV=1 | 4 | 0.387 | 0.208 | 0.000 | glycolysis |
| 729 | SP://P36183 | at4g24190 | ENPL_HORVU Endoplasmin homolog OS=Hordeum vulgare PE=2 SV=1 | 20 | 0.386 | 0.106 | 0.000 | stress |
| 730 | SP://P93267 | at3g16100 | RAB7_MESCR Ras_related protein Rab7A OS=Mesembryanthemum crystallinum PE=2 SV=1 | 4 | 0.386 | 0.232 | 0.001 | signalling |
| 731 | SP://P17784 | at2g36460 | ALF_ORYSJ Fructose_bisphosphate aldolase cytoplasmic isozyme OS=Oryza sativa subsp. japonica GN=FBA PE=1 SV=2 | 5 | 0.385 | 0.201 | 0.000 | glycolysis |
| 732 | SP://Q05326 | at4g33070 | PDC2_MAIZE Pyruvate decarboxylase 2 (Fragment) OS=Zea mays GN=PDC2 PE=2 SV=1 | 2 | 0.384 | 0.217 | 0.002 | fermentation |
| 733 | SP://Q05327 | at4g33070 | PDC3_MAIZE Pyruvate decarboxylase 3 (Fragment) OS=Zea mays GN=PDC3 PE=2 SV=1 | 2 | 0.384 | 0.217 | 0.002 | fermentation |
| 734 | SP://Q8GYB8 | at1g76690 | OPR2_ARatH 12_oxophytodienoate reductase 2 OS=Arabidopsis thaliana GN=OPR2 PE=1 SV=2 | 2 | 0.383 | 0.545 | 0.043 | hormone metabolism |
| 735 | SP://P25698 | at5g60390 | EF1A_SOYBN Elongation factor 1_alpha OS=Glycine max GN=TEFS1 PE=3 SV=2 | 8 | 0.381 | 0.177 | 0.000 | protein |
| 736 | SP://O64937 | at5g60390 | EF1A_ORYSJ Elongation factor 1_alpha OS=Oryza sativa subsp. japonica GN=REFA1 PE=2 SV=2 | 10 | 0.381 | 0.125 | 0.000 | protein |
| 737 | SP://Q9FFT4 | at5g54960 | PDC2_ARatH Pyruvate decarboxylase 2 OS=Arabidopsis thaliana GN=PDC2 PE=2 SV=1 | 4 | 0.378 | 0.167 | 0.000 | fermentation |
| 738 | SP://Q9FJA6 | at5g35530 | RS33_ARatH 40S ribosomal protein S3_3 OS=Arabidopsis thaliana GN=RPS3C PE=1 SV=1 | 2 | 0.376 | 0.399 | 0.049 | protein |
| 739 | SP://Q9M339 | at3g53870 | RS32_ARatH 40S ribosomal protein S3_2 OS=Arabidopsis thaliana GN=RPS3B PE=1 SV=1 | 2 | 0.376 | 0.399 | 0.049 | protein |
| 740 | SP://Q9SIP7 | at2g31610 | RS31_ARatH 40S ribosomal protein S3_1 OS=Arabidopsis thaliana GN=RPS3A PE=1 SV=1 | 2 | 0.376 | 0.399 | 0.049 | protein |

| | | | | | | | | |
|---|---|---|---|---|---|---|---|---|
| 741 | SP://A2ZBW5 | at1g19910 | VatL_ORYSI V_type proton atPase 16 kDa proteolipid subunit OS=Oryza sativa subsp. indica GN=VatP_P1 PE=2 SV=1 | 4 | 0.376 | 0.229 | 0.043 | transport |
| 742 | SP://O22552 | at4g34720 | VatL_VIGRR V_type proton atPase 16 kDa proteolipid subunit OS=Vigna radiata var. radiata PE=2 SV=1 | 4 | 0.376 | 0.229 | 0.043 | transport |
| 743 | SP://O24011 | at4g34720 | VatL_SOLLC V_type proton atPase 16 kDa proteolipid subunit OS=Solanum lycopersicum PE=2 SV=1 | 4 | 0.376 | 0.229 | 0.043 | transport |
| 744 | SP://P0DH92 | at4g34720 | VatL1_ARatH V_type proton atPase subunit c1 OS=Arabidopsis thaliana GN=VHA_c1 PE=2 SV=1 | 4 | 0.376 | 0.229 | 0.043 | transport |
| 745 | SP://P23957 | at1g19910 | VatL_AVESA V_type proton atPase 16 kDa proteolipid subunit OS=Avena sativa GN=VatP_P1 PE=2 SV=1 | 4 | 0.376 | 0.229 | 0.043 | transport |
| 746 | SP://P59228 | at1g19910 | VatL2_ARatH V_type proton atPase subunit c2 OS=Arabidopsis thaliana GN=VHA_c2 PE=2 SV=1 | 4 | 0.376 | 0.229 | 0.043 | transport |
| 747 | SP://P59229 | at1g75630 | VatL4_ARatH V_type proton atPase subunit c4 OS=Arabidopsis thaliana GN=VHA_c4 PE=2 SV=1 | 4 | 0.376 | 0.229 | 0.043 | transport |
| 748 | SP://P68162 | at1g19910 | VatL_BETVU V_type proton atPase 16 kDa proteolipid subunit OS=Beta vulgaris GN=VMAC1 PE=2 SV=1 | 4 | 0.376 | 0.229 | 0.043 | transport |
| 749 | SP://Q40585 | at1g19910 | VatL_TOBAC V_type proton atPase 16 kDa proteolipid subunit OS=Nicotiana tabacum PE=2 SV=1 | 4 | 0.376 | 0.229 | 0.043 | transport |
| 750 | SP://Q43434 | at1g19910 | VatL_GOSHI V_type proton atPase 16 kDa proteolipid subunit OS=Gossypium hirsutum GN=CVA16_2 PE=2 SV=1 | 4 | 0.376 | 0.229 | 0.043 | transport |
| 751 | SP://Q96473 | at1g19910 | VatL_KALDA V_type proton atPase 16 kDa proteolipid subunit OS=Kalanchoe daigremontiana PE=2 SV=1 | 4 | 0.376 | 0.229 | 0.043 | transport |
| 752 | SP://P51851 | at4g33070 | PDC2_PEA Pyruvate decarboxylase 2 (Fragment) OS=Pisum sativum GN=PDC2 PE=2 SV=1 | 3 | 0.374 | 0.210 | 0.002 | fermentation |
| 753 | SP://Q9AV81 | at2g33340 | PRP19_ORYSJ Pre_mRNA_processing factor 19 OS=Oryza sativa subsp. japonica GN=PRP19 PE=2 SV=1 | 2 | 0.373 | 0.217 | 0.000 | RNA |
| 754 | SP://P35016 | at4g24190 | ENPL_CatRO Endoplasmin homolog OS=Catharanthus roseus GN=HSP90 PE=2 SV=1 | 6 | 0.368 | 0.246 | 0.000 | stress |
| 755 | SP://Q9STX5 | at4g24190 | ENPL_ARatH Endoplasmin homolog OS=Arabidopsis thaliana GN=HSP90_7 PE=1 SV=1 | 6 | 0.368 | 0.246 | 0.000 | stress |
| 756 | SP://P29305 | at3g02520 | 1433A_HORVU 14_3_3_like protein A OS=Hordeum vulgare PE=2 SV=1 | 2 | 0.368 | 0.319 | 0.009 | signalling |
| 757 | SP://Q06967 | at3g02520 | 14336_ORYSJ 14_3_3_like protein GF14_F OS=Oryza sativa subsp. japonica GN=GF14F PE=1 SV=2 | 2 | 0.368 | 0.319 | 0.009 | signalling |
| 758 | SP://Q40034 | at5g60390 | EF1A2_HORVU Elongation factor 1_alpha OS=Hordeum vulgare GN=BLT63 PE=1 SV=1 | 12 | 0.367 | 0.144 | 0.000 | protein |
| 759 | SP://Q41803 | at5g60390 | EF1A_MAIZE Elongation factor 1_alpha OS=Zea mays GN=EF1A PE=3 SV=1 | 12 | 0.367 | 0.120 | 0.000 | protein |
| 760 | SP://P28734 | at5g11520 | AatC_DAUCA Aspartate aminotransferase_ cytoplasmic OS=Daucus carota PE=2 SV=1 | 5 | 0.366 | 0.135 | 0.000 | amino acid metabolism |
| 761 | SP://O04157 | at1g22740 | RAG3B_ARatH Ras_related protein RABG3b OS=Arabidopsis thaliana GN=RABG3B PE=1 SV=1 | 2 | 0.363 | 0.313 | 0.003 | signalling |
| 762 | SP://P14219 | at1g77120 | ADH1_PENAM Alcohol dehydrogenase 1 OS=Pennisetum americanum GN=ADH1 PE=2 SV=1 | 11 | 0.361 | 0.074 | 0.000 | fermentation |
| 763 | SP://Q84QK0 | at1g76690 | OPR1_ORYSJ 12_oxophytodienoate reductase 1 OS=Oryza sativa subsp. japonica GN=OPR1 PE=2 SV=1 | 2 | 0.360 | 0.545 | 0.002 | hormone metabolism |
| 764 | SP://A2Y5L9 | at5g01320 | PDC1_ORYSI Pyruvate decarboxylase 1 OS=Oryza sativa subsp. indica GN=PDC1 PE=2 SV=1 | 4 | 0.359 | 0.138 | 0.000 | fermentation |
| 765 | SP://Q0DHF6 | at5g01320 | PDC1_ORYSJ Pyruvate decarboxylase 1 OS=Oryza sativa subsp. japonica GN=PDC1 PE=2 SV=1 | 4 | 0.359 | 0.138 | 0.000 | fermentation |
| 766 | SP://P34823 | at5g60390 | EF1A2_DAUCA Elongation factor 1_alpha OS=Daucus carota PE=2 SV=1 | 10 | 0.353 | 0.144 | 0.000 | protein |
| 767 | SP://O23968 | at4g11600 | GPX4_HELAN Probable phospholipid hydroperoxide glutathione peroxidase OS=Helianthus annuus GN=GPXHA_2 PE=2 SV=1 | 2 | 0.353 | 0.314 | 0.000 | redox |
| 768 | SP://O49169 | at5g60390 | EF1A_MANES Elongation factor 1_alpha OS=Manihot esculenta GN=EF1 PE=3 SV=1 | 10 | 0.353 | 0.144 | 0.000 | protein |
| 769 | SP://P37833 | at5g11520 | AatC_ORYSJ Aspartate aminotransferase_ cytoplasmic OS=Oryza sativa subsp. japonica GN=Os01g0760600 PE=2 SV=1 | 10 | 0.352 | 0.092 | 0.000 | amino acid metabolism |
| 770 | SP://P46256 | at4g26530 | ALF1_PEA Fructose_bisphosphate aldolase_ cytoplasmic isozyme 1 OS=Pisum sativum PE=2 SV=1 | 2 | 0.351 | 0.427 | 0.001 | PS |
| 771 | SP://O24534 | at5g60390 | EF1A_VICFA Elongation factor 1_alpha OS=Vicia faba PE=2 SV=1 | 9 | 0.351 | 0.151 | 0.000 | protein |
| 772 | SP://O82647 | at4g33070 | PDC1_ARatH Pyruvate decarboxylase 1 OS=Arabidopsis thaliana GN=PDC1 PE=2 SV=1 | 3 | 0.350 | 0.160 | 0.000 | fermentation |
| 773 | SP://Q01K11 | at3g22200 | GatP1_ORYSI Gamma_aminobutyrate transaminase 1_ mitochondrial OS=Oryza sativa subsp. indica GN=OsI_17385 PE=3 SV=1 | 4 | 0.350 | 0.217 | 0.000 | amino acid metabolism |
| 774 | SP://Q7XN11 | at3g22200 | GatP1_ORYSJ Gamma_aminobutyrate transaminase 1_ mitochondrial OS=Oryza sativa subsp. japonica GN=OSL2 PE=1 SV=2 | 4 | 0.350 | 0.217 | 0.000 | amino acid metabolism |

| | | | | | | | | |
|---|---|---|---|---|---|---|---|---|
| 775 | SP://A2YQ76 | at4g33070 | PDC3_ORYSI Pyruvate decarboxylase 3 OS=Oryza sativa subsp. indica GN=PDC3 PE=3 SV=2 | 4 | 0.346 | 0.157 | 0.000 | fermentation |
| 776 | SP://Q0D3D2 | at4g33070 | PDC3_ORYSJ Pyruvate decarboxylase 3 OS=Oryza sativa subsp. japonica GN=PDC3 PE=2 SV=1 | 4 | 0.346 | 0.157 | 0.000 | fermentation |
| 777 | SP://P28516 | at4g33070 | PDC1_MAIZE Pyruvate decarboxylase 1 OS=Zea mays GN=PDC1 PE=2 SV=1 | 4 | 0.346 | 0.157 | 0.000 | fermentation |
| 778 | SP://P25141 | at1g77120 | ADH1_PETHY Alcohol dehydrogenase 1 OS=Petunia hybrida GN=ADH1 PE=3 SV=1 | 2 | 0.343 | 0.076 | 0.000 | fermentation |
| 779 | SP://P49299 | at2g42790 | CYSZ_CUCMA Citrate synthase_ glyoxysomal OS=Cucurbita maxima PE=1 SV=1 | 3 | 0.340 | 0.317 | 0.008 | gluconeogenese |
| 780 | SP://O24461 | at1g52280 | RAB7_PRUAR Ras_related protein Rab7 OS=Prunus armeniaca PE=2 SV=1 | 4 | 0.340 | 0.231 | 0.001 | signalling |
| 781 | SP://Q9C820 | at1g52280 | RAG3D_ARatH Ras_related protein RABG3d OS=Arabidopsis thaliana GN=RABG3D PE=2 SV=1 | 4 | 0.340 | 0.231 | 0.001 | signalling |
| 782 | SP://Q9LW76 | at3g16100 | RAG3C_ARatH Ras_related protein RABG3c OS=Arabidopsis thaliana GN=RABG3C PE=2 SV=1 | 4 | 0.340 | 0.231 | 0.001 | signalling |
| 783 | SP://Q9XER8 | at1g52280 | RAB7_GOSHI Ras_related protein Rab7 OS=Gossypium hirsutum GN=RAB7 PE=2 SV=1 | 4 | 0.340 | 0.231 | 0.001 | signalling |
| 784 | SP://P17786 | at5g60390 | EF1A_SOLLC Elongation factor 1_alpha OS=Solanum lycopersicum PE=2 SV=1 | 10 | 0.339 | 0.147 | 0.000 | protein |
| 785 | SP://P43643 | at5g60390 | EF1A_TOBAC Elongation factor 1_alpha OS=Nicotiana tabacum PE=2 SV=1 | 11 | 0.338 | 0.137 | 0.000 | protein |
| 786 | SP://P05336 | at1g77120 | ADH1_HORVU Alcohol dehydrogenase 1 OS=Hordeum vulgare GN=ADH1 PE=2 SV=1 | 10 | 0.338 | 0.063 | 0.000 | fermentation |
| 787 | SP://O82662 | at2g20420 | SUCB_ARatH Succinyl_CoA ligase [ADP_forming] subunit beta_ mitochondrial OS=Arabidopsis thaliana GN=at2g20420 | 3 | 0.335 | 0.172 | 0.013 | TCA |
| 788 | SP://Q41011 | at5g60390 | EF1A_PEA Elongation factor 1_alpha OS=Pisum sativum PE=2 SV=1 | 6 | 0.334 | 0.167 | 0.001 | protein |
| 789 | SP://Q9M039 | at5g01330 | PDC3_ARatH Pyruvate decarboxylase 3 OS=Arabidopsis thaliana GN=PDC3 PE=2 SV=1 | 2 | 0.333 | 0.177 | 0.000 | fermentation |
| 790 | SP://Q9M040 | at5g01320 | PDC4_ARatH Pyruvate decarboxylase 4 OS=Arabidopsis thaliana GN=PDC4 PE=2 SV=1 | 2 | 0.333 | 0.177 | 0.000 | fermentation |
| 791 | SP://A2XFI3 | at5g01320 | PDC2_ORYSI Pyruvate decarboxylase 2 OS=Oryza sativa subsp. indica GN=PDC2 PE=2 SV=2 | 9 | 0.333 | 0.102 | 0.000 | fermentation |
| 792 | SP://Q10MW3 | at5g01320 | PDC2_ORYSJ Pyruvate decarboxylase 2 OS=Oryza sativa subsp. japonica GN=PDC2 PE=2 SV=1 | 9 | 0.333 | 0.102 | 0.000 | fermentation |
| 793 | SP://P45851 | at1g18980 | OXO2_HORVU Oxalate oxidase 2 OS=Hordeum vulgare PE=2 SV=1 | 5 | 0.330 | 0.366 | 0.043 | stress |
| 794 | SP://Q2R8Z5 | at1g77120 | ADH1_ORYSJ Alcohol dehydrogenase 1 OS=Oryza sativa subsp. japonica GN=ADH1 PE=2 SV=2 | 8 | 0.324 | 0.067 | 0.000 | fermentation |
| 795 | SP://Q07264 | at1g77120 | ADH1_ZEALU Alcohol dehydrogenase 1 (Fragment) OS=Zea luxurians GN=ADH1 PE=3 SV=2 | 7 | 0.323 | 0.112 | 0.000 | fermentation |
| 796 | SP://O64464 | at3g60820 | PSB1_ORYSJ Proteasome subunit beta type_1 OS=Oryza sativa subsp. japonica GN=PBF1 PE=2 SV=1 | 4 | 0.322 | 0.281 | 0.005 | protein |
| 797 | SP://Q0DG35 | at5g53460 | GLT2_ORYSJ Glutamate synthase 2 [NADH]_ chloroplastic OS=Oryza sativa subsp. japonica GN=Os05g0555600 PE=2 SV=2 | 15 | 0.312 | 0.076 | 0.000 | N-metabolism |
| 798 | SP://P26759 | at1g18980 | GER3_WHEat Oxalate oxidase GF_3.8 OS=Triticum aestivum PE=1 SV=1 | 6 | 0.312 | 0.344 | 0.029 | stress |
| 799 | SP://Q40648 | at1g04690 | KCAB_ORYSJ Probable voltage_gated potassium channel subunit beta OS=Oryza sativa subsp. japonica GN=KOB1 PE=1 SV=2 | 2 | 0.312 | 0.177 | 0.000 | transport |
| 800 | SP://P51850 | at5g54960 | PDC1_PEA Pyruvate decarboxylase 1 OS=Pisum sativum GN=PDC1 PE=2 SV=1 | 2 | 0.305 | 0.205 | 0.001 | fermentation |
| 801 | SP://P51846 | at4g33070 | PDC2_TOBAC Pyruvate decarboxylase 2 OS=Nicotiana tabacum GN=PDC2 PE=2 SV=1 | 3 | 0.304 | 0.147 | 0.000 | fermentation |
| 802 | SP://P34824 | at5g60390 | EF1A1_HORVU Elongation factor 1_alpha OS=Hordeum vulgare PE=1 SV=1 | 9 | 0.304 | 0.157 | 0.000 | protein |
| 803 | SP://Q0JKD0 | at5g53460 | GLT1_ORYSJ Glutamate synthase 1 [NADH]_ chloroplastic OS=Oryza sativa subsp. japonica GN=Os01g0681900 PE=2 SV=1 | 26 | 0.289 | 0.049 | 0.000 | N-metabolism |
| 804 | SP://P00333 | at1g77120 | ADH1_MAIZE Alcohol dehydrogenase 1 OS=Zea mays GN=ADH1 PE=2 SV=1 | 8 | 0.287 | 0.083 | 0.000 | fermentation |
| 805 | SP://P48977 | at1g77120 | ADH_MALDO Alcohol dehydrogenase OS=Malus domestica GN=ADH PE=2 SV=1 | 2 | 0.286 | 0.101 | 0.000 | fermentation |
| 806 | SP://Q9LXS6 | at3g58750 | CISY2_ARatH Citrate synthase 2_ peroxisomal OS=Arabidopsis thaliana GN=CSY2 PE=2 SV=1 | 3 | 0.285 | 0.186 | 0.001 | gluconeogenese |
| 807 | SP://Q9LV03 | at5g53460 | GLUT1_ARatH Glutamate synthase 1 [NADH]_ chloroplastic OS=Arabidopsis thaliana GN=GLT1 PE=2 SV=2 | 8 | 0.284 | 0.078 | 0.000 | N-metabolism |
| 808 | SP://P06525 | at1g77120 | ADH1_ARatH Alcohol dehydrogenase class_P OS=Arabidopsis thaliana GN=ADH1 PE=1 SV=2 | 3 | 0.283 | 0.094 | 0.000 | fermentation |

| | | | | | | | | |
|---|---|---|---|---|---|---|---|---|
| 809 | SP://P12886 | at1g77120 | ADH1_PEA Alcohol dehydrogenase 1 OS=Pisum sativum PE=3 SV=1 | 3 | 0.283 | 0.094 | 0.000 | fermentation |
| 810 | SP://P13603 | at1g77120 | ADH1_TRIRP Alcohol dehydrogenase 1 OS=Trifolium repens GN=ADH1 PE=2 SV=1 | 3 | 0.283 | 0.094 | 0.000 | fermentation |
| 811 | SP://P45850 | at1g18970 | OXO1_HORVU Oxalate oxidase 1 OS=Hordeum vulgare PE=1 SV=1 | 3 | 0.280 | 0.416 | 0.027 | stress |
| 812 | SP://P10847 | at1g77120 | ADH2_HORVU Alcohol dehydrogenase 2 OS=Hordeum vulgare GN=ADH2 PE=3 SV=1 | 7 | 0.266 | 0.081 | 0.000 | fermentation |
| 813 | SP://Q03460 | at5g53460 | GLSN_MEDSA Glutamate synthase [NADH]_ amyloplastic OS=Medicago sativa PE=1 SV=1 | 6 | 0.265 | 0.102 | 0.000 | N-metabolism |
| 814 | SP://P52894 | at1g72330 | ALA2_HORVU Alanine aminotransferase 2 OS=Hordeum vulgare PE=1 SV=1 | 7 | 0.262 | 0.077 | 0.000 | amino acid metabolism |
| 815 | SP://P10848 | at1g77120 | ADH3_HORVU Alcohol dehydrogenase 3 OS=Hordeum vulgare GN=ADH3 PE=3 SV=1 | 11 | 0.255 | 0.055 | 0.000 | fermentation |
| 816 | SP://P04707 | at1g77120 | ADH2_MAIZE Alcohol dehydrogenase 2 OS=Zea mays GN=ADH2 PE=2 SV=1 | 6 | 0.251 | 0.069 | 0.000 | fermentation |
| 817 | SP://P14673 | at1g77120 | ADH1_SOLTU Alcohol dehydrogenase 1 OS=Solanum tuberosum GN=ADH1 PE=2 SV=1 | 2 | 0.249 | 0.199 | 0.000 | fermentation |
| 818 | SP://P28032 | at1g77120 | ADH2_SOLLC Alcohol dehydrogenase 2 OS=Solanum lycopersicum GN=ADH2 PE=2 SV=2 | 2 | 0.249 | 0.199 | 0.000 | fermentation |
| 819 | SP://P14674 | at1g77120 | ADH2_SOLTU Alcohol dehydrogenase 2 OS=Solanum tuberosum GN=ADH2 PE=2 SV=1 | 4 | 0.246 | 0.146 | 0.000 | fermentation |
| 820 | SP://P14675 | at1g77120 | ADH3_SOLTU Alcohol dehydrogenase 3 OS=Solanum tuberosum GN=ADH3 PE=2 SV=1 | 4 | 0.246 | 0.146 | 0.000 | fermentation |
| 821 | SP://Q0ITW7 | at1g77120 | ADH2_ORYSJ Alcohol dehydrogenase 2 OS=Oryza sativa subsp. japonica GN=ADH2 PE=2 SV=2 | 8 | 0.246 | 0.065 | 0.000 | fermentation |
| 822 | SP://P07803 | at2g16060 | HBL_TRETO Non_symbiotic hemoglobin OS=Trema tomentosa GN=GLB1 PE=1 SV=1 | 2 | 0.208 | 0.237 | 0.000 | redox |
| 823 | SP://Q42831 | at2g16060 | HBL_HORVU Non_symbiotic hemoglobin OS=Hordeum vulgare GN=HB PE=1 SV=1 | 3 | 0.205 | 0.213 | 0.000 | redox |
| 824 | SP://P81406 | at2g24270 | GAPN_PEA NADP_dependent glyceraldehyde_3_phosphate dehydrogenase OS=Pisum sativum GN=GAPN PE=1 SV=1 | 2 | 0.181 | 0.263 | 0.003 | glycolysis |
| 825 | SP://P93338 | at2g24270 | GAPN_NICPL NADP_dependent glyceraldehyde_3_phosphate dehydrogenase OS=Nicotiana plumbaginifolia GN=GAPN | 3 | 0.164 | 0.232 | 0.000 | glycolysis |
| 826 | SP://Q43272 | at2g24270 | GAPN_MAIZE NADP_dependent glyceraldehyde_3_phosphate dehydrogenase OS=Zea mays GN=GPN1 PE=2 SV=1 | 3 | 0.164 | 0.232 | 0.000 | glycolysis |
| 827 | SP://Q8LK61 | at2g24270 | GAPN_WHEat NADP_dependent glyceraldehyde_3_phosphate dehydrogenase OS=Triticum aestivum GN=GAPN PE=1 SV=2 | 3 | 0.164 | 0.232 | 0.000 | glycolysis |
| 828 | SP://P0C0M2 | at4g37390 | GH32_ORYSJ Probable indole_3_acetic acid_amido synthetase GH3.2 OS=Oryza sativa subsp. japonica GN=GH3.2 PE=2 SV=1 | 3 | 0.111 | 0.414 | 0.000 | hormone metabolism |

[a] Protein ID is according to the green plant database in UniProt; [b] MP means matched peptides (cutoff value: <2); [c] Ratio is calculated using treatments divide control; [d] Functional category is obtained from MapMan bin code.

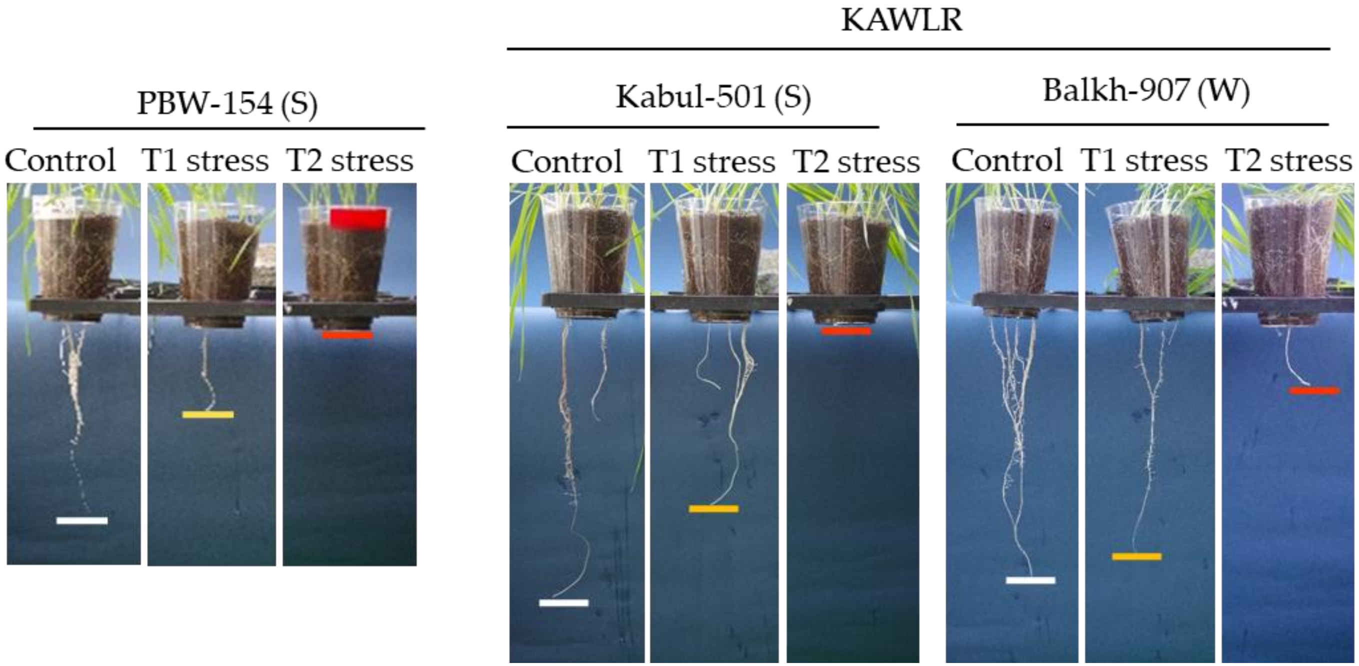


**Figure S1.** Photographs of one spring-type modern check (PBW-154), one spring-type KAWLR (Kabul-501) and one winter-type KAWLR (Balkh-907) showing variations in root emersion through the bottom hole of upper cup after 2 weeks of treatment initiation. KAWLR, Kihara Afghan wheat landrace; S, spring-type; W, winter-type.

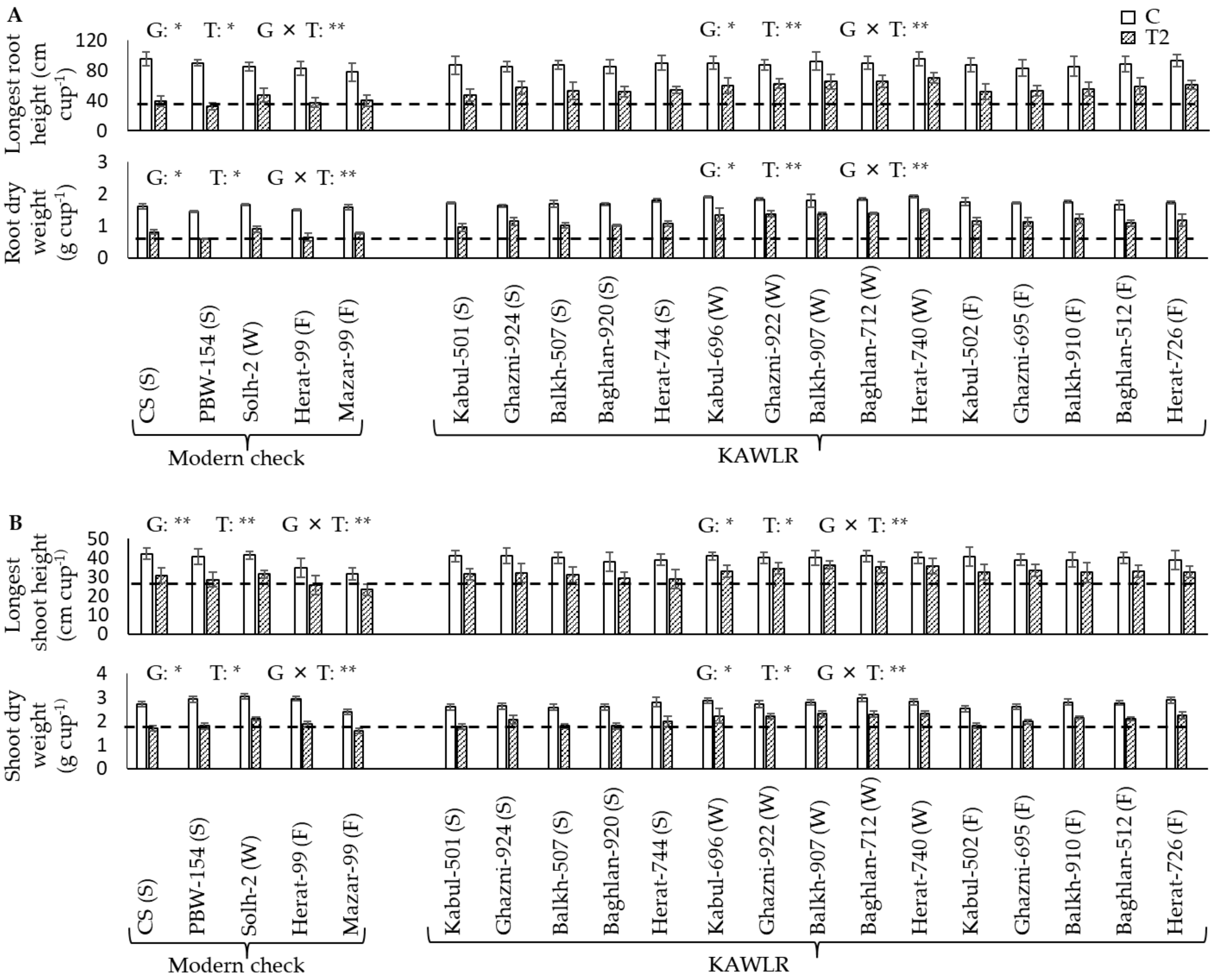


**Figure S2.** Effects of T2 stress condition on root and shoot traits of wheat genotypes and growth habits. **(A)** Longest root length (*above graph*) and root dry weight (*below graph*). **(B)** Longest shoot length (*above graph*) and shoot dry weight (below graph). Values are means ± SE (n = 4). Single and double asterisks indicate significant differences at $p > 0.05$, $p > 0.01$ levels by analysis of variance (ANOVA), respectively. S, spring-type; W, winter-type; F, facultative-type; G, genotypes; T, treatments.

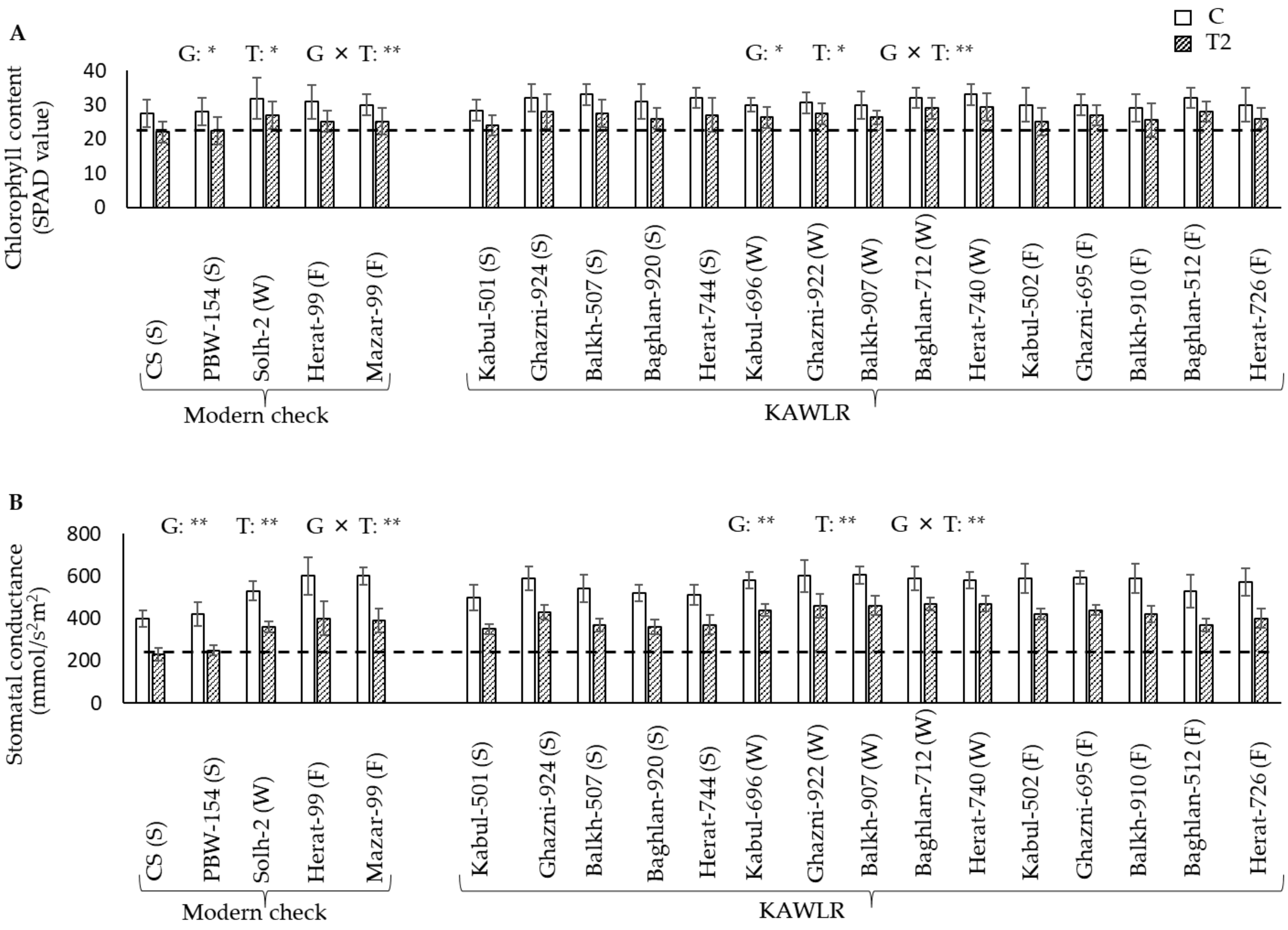


**Figure S3.** Effects of T2 stress condition on chlorophyll content **(A)** and stomatal conductance **(B)** of wheat genotypes and growth habits. Values are means ± SE (n=10). * $p > 0.05$, ** $p > 0.01$. G, genotypes; T, treatments.

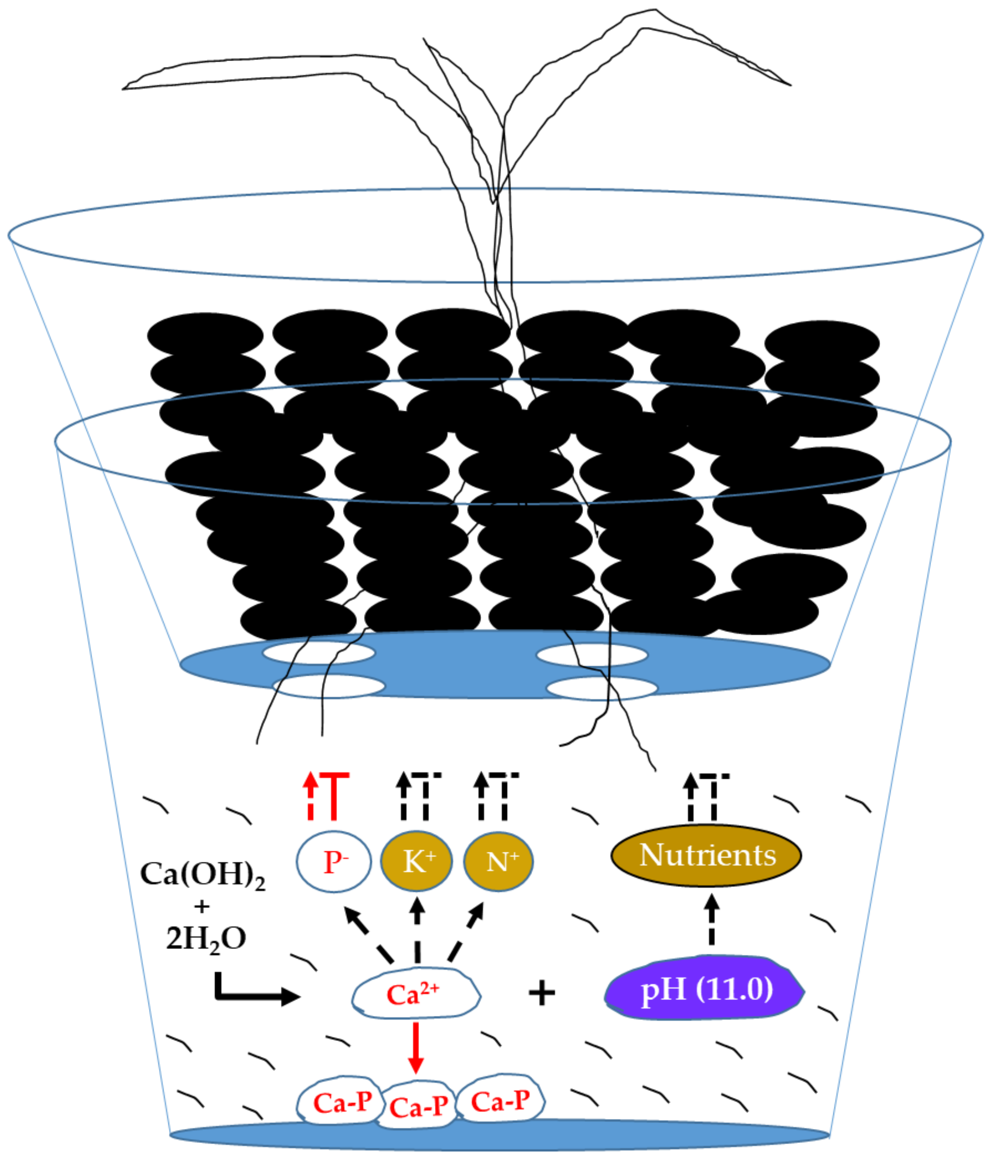


**Figure S4.** Design flaws of the T2 double cup experiment showing nutritional deficient state that can be caused by combined high Ca and high pH. For further details see Discussion section.

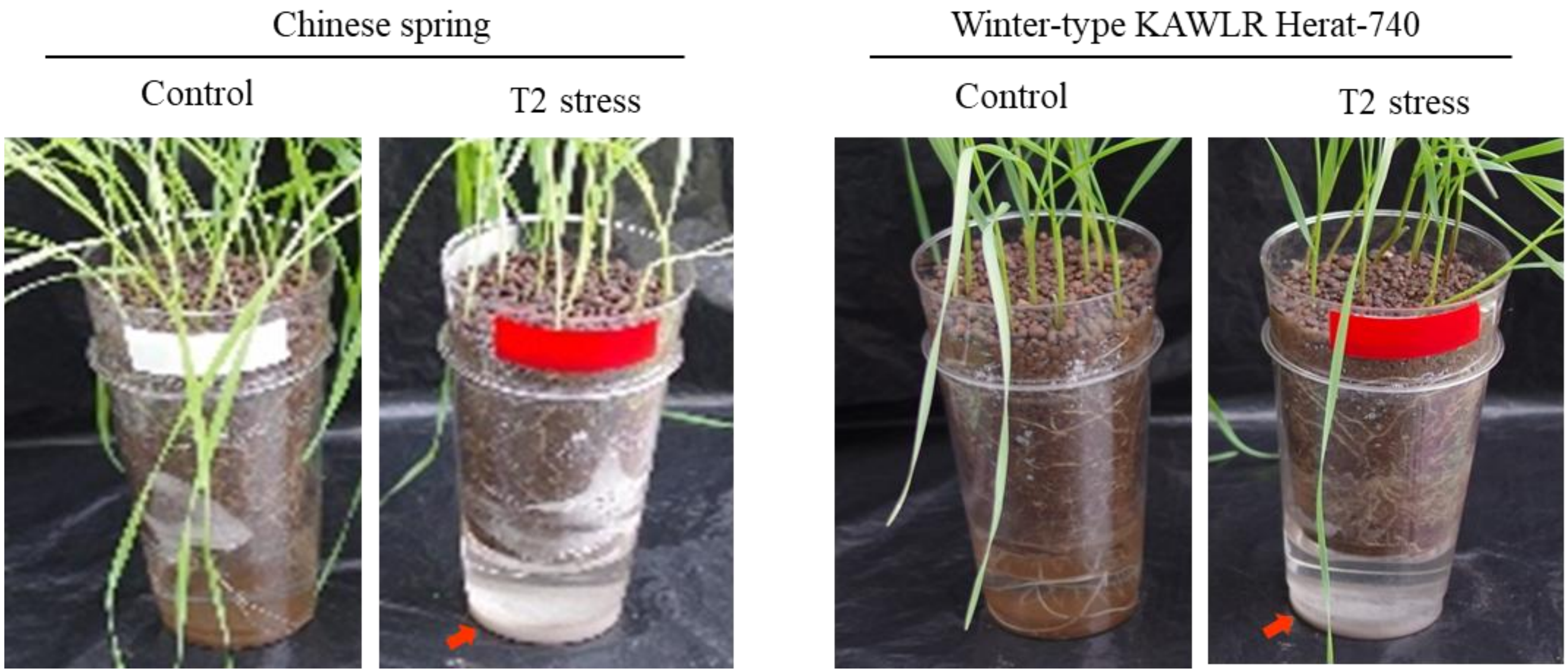


**Figure S5.** Images on whitish sedimentation of control vs T2 stress in Chinese spring and winter-type KAWLR genotype Herat-740 after one week of treatment initiation.
